# Global population genetic structure and demographic trajectories of the black soldier fly, *Hermetia illucens*

**DOI:** 10.1186/s12915-021-01029-w

**Published:** 2021-05-05

**Authors:** Cengiz Kaya, Tomas N. Generalovic, Gunilla Ståhls, Martin Hauser, Ana C. Samayoa, Carlos G. Nunes-Silva, Heather Roxburgh, Jens Wohlfahrt, Ebenezer A. Ewusie, Marc Kenis, Yupa Hanboonsong, Jesus Orozco, Nancy Carrejo, Satoshi Nakamura, Laura Gasco, Santos Rojo, Chrysantus M. Tanga, Rudolf Meier, Clint Rhode, Christine J. Picard, Chris D. Jiggins, Florian Leiber, Jeffery K. Tomberlin, Martin Hasselmann, Wolf U. Blanckenhorn, Martin Kapun, Christoph Sandrock

**Affiliations:** 1 Department of Livestock Sciences, Research Institute of Organic Agriculture (FiBL), Frick, Switzerland; 2 Department of Evolutionary Biology and Environmental Sciences, University of Zurich, Zurich, Switzerland; 3 Department of Zoology, University of Cambridge, Cambridge, UK; 4 Zoology unit, Finnish Museum of Natural History, Helsinki, Finland; 5 California Department of Food and Agriculture, Plant Pest Diagnostics Branch, Sacramento, USA; 6 Department of Entomology, National Chung Hsing University, Taichung, Taiwan; 7 Department of Genetics and Biotechnology Graduate Program, Federal University of Amazonas, Manaus, Brazil; 8 Biological and Environmental Sciences, University of Stirling, Stirling, UK; 9 Biotechnology and Nuclear Agriculture Research Institute, Ghana Atomic Energy Commission, Accra, Ghana; 10 CABI, Delémont, Switzerland; 11 Department of Entomology, Khon Kaen University, Khon Kaen, Thailand; 12 Department of Agricultural Sciences and Production, Zamorano University, Zamorano, Honduras; 13 Department of Biology, Universidad del Valle, Santiago de Cali, Colombia; 14 Crop, Livestock and Environmental Division, Japan International Research Center for Agricultural Sciences (JIRCAS), Tsukuba, Japan; 15 Department of Agricultural, Forest and Food Sciences, University of Turin, Turin, Italy; 16 Department of Environmental Sciences and Natural Resources, University of Alicante, Alicante, Spain; 17 International Centre of Insect Physiology and Ecology (icipe), Nairobi, Kenya; 18 Department of Biological Sciences, National University of Singapore, Singapore, Singapore; 19 Department of Genetics, Stellenbosch University, Stellenbosch, Republic of South Africa; 20 Department of Biology, Indiana University - Purdue University Indianapolis, Indianapolis, USA; 21 Department of Entomology, Texas A&M University, College Station, USA; 22 Department of Livestock Population Genomics, University of Hohenheim, Stuttgart, Germany; 23 Department of Cell and Developmental Biology, Medical University of Vienna, Vienna, Austria

**Keywords:** Allelic richness, Approximate Bayesian computation, Diptera, Founder effect, Genetic differentiation, Genetic drift, Invasive species, Isolation by distance, Serial introductions, Stratiomyidae

## Abstract

**Background:**

The black soldier fly (*Hermetia illucens*) is the most promising insect candidate for nutrient-recycling through bioconversion of organic waste into biomass, thereby improving sustainability of protein supplies for animal feed and facilitating transition to a circular economy. Contrary to conventional livestock, genetic resources of farmed insects remain poorly characterised. We present the first comprehensive population genetic characterisation of *H. illucens*. Based on 15 novel microsatellite markers, we genotyped and analysed 2862 individuals from 150 wild and captive populations originating from 57 countries on seven subcontinents.

**Results:**

We identified 16 well-distinguished genetic clusters indicating substantial global population structure. The data revealed genetic hotspots in central South America and successive northwards range expansions within the indigenous ranges of the Americas. Colonisations and naturalisations of largely unique genetic profiles occurred on all non-native continents, either preceded by demographically independent founder events from various single sources or involving admixture scenarios. A decisive primarily admixed Polynesian bridgehead population serially colonised the entire Australasian region and its secondarily admixed descendants successively mediated invasions into Africa and Europe. Conversely, captive populations from several continents traced back to a single North American origin and exhibit considerably reduced genetic diversity, although some farmed strains carry distinct genetic signatures. We highlight genetic footprints characteristic of progressing domestication due to increasing socio-economic importance of *H. illucens*, and ongoing introgression between domesticated strains globally traded for large-scale farming and wild populations in some regions.

**Conclusions:**

We document the dynamic population genetic history of a cosmopolitan dipteran of South American origin shaped by striking geographic patterns. These reflect both ancient dispersal routes, and stochastic and heterogeneous anthropogenic introductions during the last century leading to pronounced diversification of worldwide structure of *H. illucens*. Upon the recent advent of its agronomic commercialisation, however, current human-mediated translocations of the black soldier fly largely involve genetically highly uniform domesticated strains, which meanwhile threaten the genetic integrity of differentiated unique local resources through introgression. Our in-depth reconstruction of the contemporary and historical demographic trajectories of *H. illucens* emphasises benchmarking potential for applied future research on this emerging model of the prospering insect-livestock sector.

**Supplementary Information:**

The online version contains supplementary material available at 10.1186/s12915-021-01029-w.

## Background

Insects are considered one of the most promising agricultural resources to address the socio-economic challenges of a continuously growing human population due to their dual sustainability advantage [1–3]. On the one hand, increasing amounts of organic waste from agricultural food chains, livestock production, and households cause severe ecological footprints [4–6]. On the other hand, conventional protein supplies for livestock and aquaculture feed are becoming increasingly unsustainable due to land and water competition with primary food production, thereby reinforcing global environmental impacts and destabilisation of ecosystems [7–9]. Substituting soybean- and fishmeal-based protein components in animal feed with insect biomass, produced from efficient bioconversion of agricultural waste, provides a mitigation strategy and facilitates sustainable nutrient-recycling [10–13].

The black soldier fly (BSF), *Hermetia illucens* (L. 1758; Diptera: Stratiomyidae), is a particularly promising candidate and considered the ‘crown jewel’ of the fast-growing insect-farming industry [14]. BSF larvae are voracious feeders of a broad variety of organic matter of both plant and animal origin [15–18]. Remarkable feeding efficiencies and the ability to upcycle nutrient-poor substrates into protein-rich insect biomass are prime characteristics of commercial interest [19–21]. Given their valuable nutrient profiles [22, 23], BSF larvae are highly suited for partially replacing soybean and fishmeal in diets for poultry [24, 25], swine [26, 27] and aquaculture species [28, 29]. Moreover, their high-fat contents could serve as a source for biodiesel [30, 31]. Accordingly, academic research interests in this insect have rapidly increased, which has resulted in more than a thousand scientific publications over the last 5 years, paralleled by the advent of an insect ‘mini-livestock’ production industry across the globe.

In contrast to conventional livestock, genetic resources of farmed insects remain poorly characterised [32]. Phenotypic performance variation among BSF populations has only been addressed by Zhou et al. [33], although genetic distances between the studied populations were not reported. Recent evidence suggests variation among global studies for any given life-history trait, such as larval performance or body composition profiles, could be the result of underlying genetic differences between populations [34]. However, a comprehensive analysis of nuclear genetic variation within and among worldwide BSF populations, which is urgently needed to understand global population structure and its phenotypic correlates, is lacking. In order to assist anticipated efforts for advanced BSF breeding based on the recently published genomic resources [35, 36], it is imperative to generate a comprehensive inventory of the global population genetic architecture and geographic structure of BSF and decipher its evolutionary history.

In this context, historic documentation of organism distributions represents a crucial basis. The BSF is considered cosmopolitan across tropical, subtropical and temperate regions [37–39], and is therefore the most widely distributed stratiomyid in the world. It is purported to be indigenous to the Americas, where BSF now occur from Argentina to Canada [38–40]. A more explicit origin of the species within South America and a potentially more recent colonisation of North America have remained speculative [39]. Beyond the Americas, earliest documentations from Australasia date back to 1930 on Hawaii [41], and then during the 1930s and 1950s across other Pacific islands, eastern Australia, Southeast Asia, and New Zealand ([37–40] and references therein). The oldest African records date back to 1914 (South Africa) and 1945 (Liberia) [42], whereas documentation in other African countries has been accumulating only since the late 1950s [38, 42]. In Europe, BSF was first reported from Malta in 1926 [39], since the 1950s from France, Italy, and Spain, and only since the late 1980s from temperate European regions ([38–40, 42] and references therein). Augmented academic and economic interest arose only recently, however, largely building upon the pioneering research carried out in the USA during the 1990s [43, 44]. Due to the ubiquitous distribution of one BSF strain that was originally used in the USA (J.K. Tomberlin, personal communication), captive populations used for farming and research across continents most likely build on a narrow genetic basis.

Contrary to ancient natural dispersal and presumably highly stochastic, unintended anthropogenic introductions of BSF into non-native areas that occurred in recent history, the presently increased global farming activity is likely to result in more extensive translocations of genetically uniform captive populations, possibly reinforcing one-directional admixture worldwide. A recent investigation of the global phylogeography of BSF based on mitochondrial COI marker sequences [45] detected substantial global sequence divergence of up to 4.9%, and concluded that puzzling haplotype prevalence in various regions of the world could reflect recurrent recent introductions of widely farmed BSF strains of shared ancestry across continents. However, the single-locus maternally inherited COI mitochondrial marker is comparatively evolutionary conserved, which impedes resolution at the population level and does not allow distinguishing selection from demographic signals [46]. Hence, there is an urgent need for adequate nuclear genetic markers that are readily applicable and with high resolution for documenting the amount and distribution of nuclear genetic diversity worldwide, allocating samples to distinct genetic clusters and inferring the evolutionary forces that have shaped the natural distribution and global population structure of BSF. Considering putatively ongoing influences of captive populations on local wild populations via intraspecific hybridisation, a thorough survey to identify distinct genetic resources and their relationships is pivotal. This could set the stage for uncovering genetic adaptations across native and newly colonised geographic regions within an evolutionary ecology framework of a highly invasive species, which would moreover facilitate the future identification of genetic correlations to performance trait variation useful for the mass production of optimised strains.

This study aimed to ascertain the global population genetic structure and elucidate the contemporary demographic history of *H. illucens*. A comprehensive sample of 2862 individuals from 150 wild and captive populations collected in 57 countries on seven subcontinents were genotyped based on 15 newly developed polymorphic microsatellites. We used this large-scale dataset for population genetics analyses to characterise genetic diversity and to identify distinct genetic clusters of BSF worldwide. We further applied coalescence-based simulations for demographic inference with Approximate Bayesian Computation (ABC). These complementary analyses allowed the following questions to be addressed: (1) Can genetic hotspots reveal the geographic origin of the species? (2) Do population genetic patterns allow the reconstruction of native range expansions and the demographic trajectories of historic dispersal routes into non-native areas? and (3) To what extent do population genetic characteristics of indigenous and naturalised wild populations differ from captive populations in modern mass production facilities in various regions worldwide? We moreover hypothesise that more than two decades of captive breeding and common global trade of a well-described North American BSF laboratory population has left a detectable genetic footprint accompanying domestication. Based on that conjecture, we further aimed to investigate possible human-mediated impacts of domesticated BSF strains increasingly used for farming on present biogeographic population genetic patterns of this cosmopolitan insect, with special emphasis on the direction and the extent of local genetic introgression.

## Results

### Genetic markers and sample characteristics

All 2862 individuals represented unique multilocus genotypes (MLGs; Table S1, Additional file 1), indicating that the markers developed (Table S2, Additional file 2) provide a robust and informative tool for BSF population genetics (Figure S1, Additional file 2). The mean number of alleles per locus was 24.5 (± 8.3), and locus-specific characteristics are summarised in Table 1. On average, populations comprised 19.1 individuals (ranging between 5 and 50) that harboured 61.73 ± 15.47 alleles across loci and, when rarefied to five diploid individuals, exhibited a mean allelic richness of 3.04 ± 0.49 per locus (Table S3, Additional file 2). Locus-specific deviations from Hardy-Weinberg equilibrium (HWE) within populations were detected at low levels (Table 1) and summed up to merely 3.5% of all comparisons across 150 populations (Figure S2, Additional file 2). Significant homozygote excess across loci within populations was detected for 54 populations (Table S3, Additional file 2). Indications for null alleles were significant for the majority of loci; however, the absence of null allele homozygotes throughout the entire data set suggests potential null alleles rarely segregate at substantial frequencies. Of 15,750 tests for linkage disequilibrium (LD) among locus pairs across populations, 113 were significant, indicating independent marker segregation. A BLAST search against the *H. illucens* chromosomal assembly GCA_905115235.1 [36] confirmed reasonable coverage of the genome by the novel markers: five chromosomes (all but the smaller chromosomes 6 and 7) are covered, and multiple loci (up to five) reside on chromosomes 2, 3 and 4, with average pairwise distances of 45.5, 32.7 and 54.0 Mbp, respectively, between them.

**Table 1:** Microsatellite-specific characteristics across 150 black soldier fly populations

Locus	***N***_**A**_	***F***_**ST**_	***F***_**IS**_	***H***_**O**_	***H***_**E**_	HWE_**Dev**_	***A***_**R**_	***A***_**U**_
Hi_1-1	29	**0.235**	**0.084**	0.599	0.648	4	3.664	0.035
Hi_1-2	36	**0.235**	**0.032**	0.607	0.621	3	3.595	0.194
Hi_1-3	31	**0.214**	0.043	0.434	0.449	3	2.423	0.194
Hi_1-4	30	**0.168**	**0.086**	0.537	0.587	7	3.078	0.367
Hi_1-5	17	**0.248**	**0.125**	0.501	0.570	5	2.988	0.059
Hi_2-1	12	**0.293**	− 0.018	0.395	0.382	0	2.346	0.083
Hi_2-2	25	**0.202**	**0.080**	0.644	0.690	8	3.840	0.200
Hi_2-3	19	**0.290**	**0.109**	0.550	0.607	4	3.193	0.000
Hi_2-4	31	**0.246**	**0.082**	0.584	0.631	3	3.494	0.000
Hi_2-5	8	**0.259**	− 0.026	0.414	0.403	2	2.219	0.125
Hi_3-1	30	**0.279**	**0.118**	0.333	0.369	3	2.328	0.167
Hi_3-2	28	**0.211**	**0.070**	0.599	0.643	6	3.475	0.107
Hi_3-3	24	**0.179**	**0.118**	0.570	0.647	13	3.553	0.083
Hi_3-4	32	**0.298**	**0.176**	0.474	0.566	11	3.141	0.063
Hi_3-5	16	**0.248**	**0.130**	0.538	0.607	7	3.191	0.000
*overall*	*368*	***0.239***	***0.085***	*0.519*	*0.561*	*79*	*3.102*	*0.122*

*N*_*A*_ number of alleles, *F*_*ST*_ fixation index, *F*_*IS*_ inbreeding coefficient, *H*_*O*_*/H*_*E*_ observed and expected heterozygosity, *HWE*_*Dev*_ number of significant deviations from Hardy-Weinberg equilibrium across all 150 populations, *A*_*R*_ mean allelic richness per population (rarefied to five diploid individuals), *A*_*U*_ proportion of unique alleles (detected only once). Significant *F*-statistics are highlighted in bold

### Pronounced population structure is shaped by geographic origin and wild versus captive provenance

**Fig. 1.**
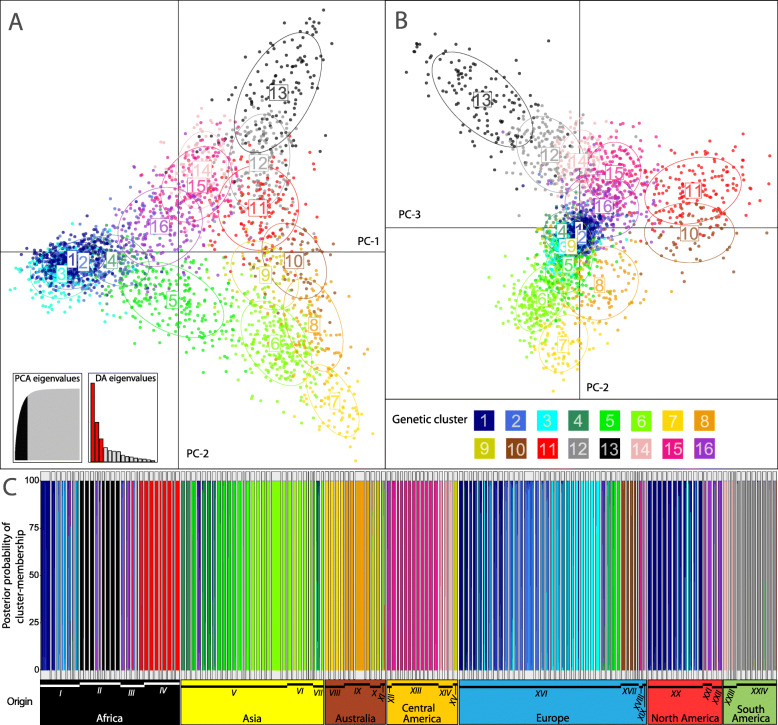
Global population genetic patterns of *Hermetia illucens.* Discriminant analysis of principal components depicting all 2862 multilocus genotypes assigned to 16 distinct genetic clusters, independent of the original populations sampled (see also Table 2, Table S6, Additional file 1). **a** Axes 1 and 2 and **b** axes 2 and 3. Dots show individuals and numbered labels denote cluster means, both arbitrarily coloured as per the key below panel **b**. **c** Posterior probabilities of membership to inferred genetic clusters (*K*=16) for all individuals (stacked bar plots) arranged as vertical bands within their original populations (Table S3, Additional file 2), indicating admixture proportions with cluster colours as defined in panels **a** and **b**. Populations are framed by grey boxes whose widths reflect the number of individuals displayed. The bottom part of panel **c** shows population grouping according to subcontinental origins using colours that differ from those used for genetic clusters. Population groups labelled with roman numbers refer to major geographic regions and provenances: I: entire Africa—captive; II: west—wild; III: central-east—wild; IV: south-east—wild; V: entire Asia—captive; VI: south-east continental—wild; VII: south-east insular—wild; VIII: west—wild & captive; IX: east—captive; X: southern Polynesia—wild & captive; XI: northern Polynesia—captive; XII: north—wild; XIII: central regions—wild & captive; XIV: south—wild; XV: Caribbean—wild; XVI: entire Europe—captive; XVII: west/central—wild; XVIII: south—wild; XIX: south-east—wild; XX: entire North America—captive; XXI: west—wild; XXII: south-east—wild; XXIII: north-west—wild & captive; XXIV: central-east—wild & captive.

**Fig. 2.**
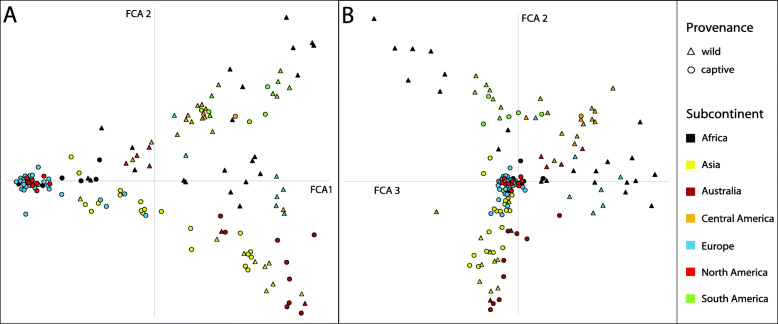
Factorial correspondence analysis (FCA) of 150 *Hermetia illucens* populations. Population ‘barycentres’ labelled according to provenance status and subcontinent of origin are projected in multidimensional space based on FCA axes 1 and 2 (panel **a**) and axes 2 and 3 (panel **b**), which together explain 22.2% of the total variance. Individual-based multivariate ordination according to population provenance nested within subcontinent is shown as a complementary analysis in Figure S4, Additional file 2.

**Fig. 3.**
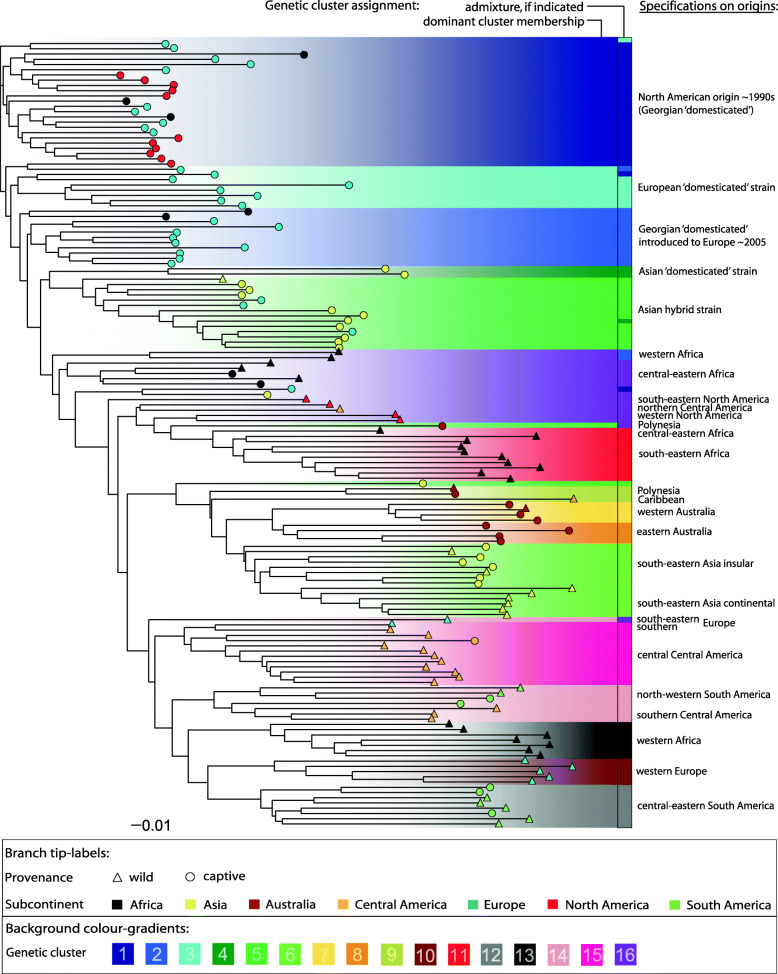
Dendrogram of Cavalli-Sforza and Edwards chord distances of 150 *Hermetia illucens* populations. Population cluster allocations (based on the majority of assigned individuals) are gradient-highlighted in the background according to Fig. 1. For some highly admixed populations, the second-most abundant clusters are indicated in a separate column. Labels at the branch-tips represent population provenances and subcontinental origins as defined in Fig. 2. Additional information on major geographic regions of origin is specified for wild populations and selected strains (see Table 2)

We characterised general patterns of population differentiation and applied complementary approaches to identify key factors shaping BSF global population genetic structure. Maximum-likelihood (ML)-based cluster analyses and model selection using KIC goodness-of-fit statistics supported *K* = 16 as the optimal number of distinct genetic clusters, whose global structure, derived from discriminant analysis of principal components (DAPC), is shown in Fig. 1a,b (see also Figure S3 A-D, Additional file 2). Minimal gene flow between clusters across the majority of populations is supported by corresponding admixture analysis of individual MLGs (Fig. 1c). Characteristics of the populations allocated to the 16 distinct clusters (Table 2) revealed that genetic clustering largely reflects geography and provenance (i.e. wild vs. captive origin) within but also across regions, including specific breeding-mediated signatures of some farmed BSF strains (see also Tables S3-S6, Additional file 2). Factorial correspondence analysis (FCA) accounting for subcontinental origin (see “Methods”) and provenance at the whole population level (complementary to MLG cluster assignment) stressed these two key drivers of global population structure: the first axis separates a majority of closely related captive populations of broad geographic origin from all the rest (Fig. 2a), while the second and third axes centre this captive group and highlight geographic structure largely independent of population provenance (Fig. 2b). We further quantified pairwise population genetic differentiation ranging from *F*_ST_ = 0 to 0.626 (> 99% of the 11,175 pairwise tests were significant; *p* < 0.0001) to document substantial global genetic structure with an overall *F*_ST_ = 0.239 (Figure S5, Additional file 2). A combined view of ML-inferred cluster assignments mapped on a neighbour-joining tree constructed from population pairwise Cavalli-Sforza chord distances (*D*_CH_) is shown in Fig. 3.

**Table 2:** Characteristics of black soldier fly populations assigned to distinct genetic clusters

Cluster (***K*** = 16)	No. Pops	Geographic origin, provenance status, indicated admixture and traceable breeding history
1	25	Captive North American populations plus recent introductions thereof in Europe and Africa (exclusively captive)
2	15	Captive European and African populations (derived from captive North American populations introduced around 2005) plus introgressed wild African populations
3	7	Captive European populations (recent breeding programme: captive North American origin)
4	3	Captive Asian populations (recent breeding programme: captive North American origin introgressed by wild Asian populations)
5	14	Captive and wild Asian and captive European populations (hybrids between wild Asian and captive North American populations)
6	15	Wild and captive Asian populations plus admixed captive Australian-Polynesian populations
7	4	Wild and captive Australian populations (west)
8	4	Captive Australian populations (east)
9	3	Wild and captive Australian-Polynesian and wild Central American (Caribbean) populations
10	5	Wild European populations (west)
11	10	Wild African populations (south-east)
12	8	Wild and captive South American populations (central-east)
13	7	Wild African populations (west)
14	8	Wild and captive South American (north-west) and wild Central American (south) plus wild European (south-east) populations
15	12	Wild and captive Central America (central regions) and wild European (south) populations
16	10	Wild North American (west, south-east), wild Central American (north), wild and captive African (central-east) populations plus admixed wild European (south-east), captive Asian and captive Australian-Polynesian populations

Numbers of populations assigned to each of the 16 clusters (Fig. 1) based on the majority of individuals, including the influence of admixture (if detected), relevant information on geographic origin, occurrence in the wild and/or captivity (provenance), and breeding history if traceable (see Fig. 3, Table S4, Additional file 2). For genetic diversity and pairwise differentiation of inferred genetic clusters, independent of sampling populations, see Table S6, Additional file 2

The complex overall population structure captured by the complementary approaches (Figs. 1, 2 and 3) is mediated by overarching factors of geographic distribution and population provenance. To further disentangle their relative influences, we progressively partitioned the dataset according to subcontinental origin and provenance and applied linear mixed effect models on rarefied allelic richness (*A*_*R*_), analysis of molecular variance (AMOVA; Table S7, Additional file 2), and isolation by distance (IBD) based on Mantel tests.

On a global scale, *A*_*R*_ of wild populations was significantly higher than for captive samples (*p* < 0.001; Table S8, Additional file 2). Separate AMOVAs of wild and captive populations across geographic regions indicated that captive populations are more structured across subcontinents than wild populations. Conversely, wild populations exhibited more pronounced structure between populations within subcontinents, with less variation between individuals within populations (Table 3a). Globally, IBD was slightly stronger for captive populations (*r* = 0.339 vs. *r* = 0.317; Table S9, Additional file 2). A breakdown of these associations for both (pooled) provenances (*r* = 0.214) clearly indicates genetic mismatches of wild and captive populations in some regions, as also reflected by the FCA (Fig. 2) and genetic distance networks (Fig. 3, Figure S5, Additional file 2). We then evaluated whether captive provenance aligns or contrasts with the geographic population structure of wild populations within subcontinents. On all subcontinents, apart from the Americas, IBD across wild and captive populations was lower compared to only wild populations (Table S9, Additional file 2): while IBD differed only slightly in Africa (*r* = 0.411 vs. *r* = 0.497), the difference was much stronger across Australasia (*r* = 0.236 vs. *r* = 0.413), and substantial in Europe (*r* = 0.019 vs. *r* = 0.389).

**Table 3:** Analyses of molecular variance according to successive hierarchical grouping

a)		Proportion of variance (%)							
	Source of variance	Wild			Captive		Combined provenances		
	Between subcontinents	**7.41**			**10.18**		**7.79**		
	Between populations within subcontinents	**17.16**			**13.10**		**17.07**		
	Between individuals within populations	**5.12**			**7.32**		**6.38**		
	Within individuals	**70.32**			**69.40**		**68.76**		
b)		Proportion of variance (%)							
	Source of variance	Africa	Asia	Australia	Central America	Europe	North America	South America	All regions
	Between provenances	**9.72**	**5.36**	− 1.18	− 0.66	**16.66**	**10.43**	− 0.09	**4.28**
	Between populations within provenances	**16.97**	**18.01**	**18.78**	**15.42**	**12.48**	**6.83**	**13.57**	**21.31**
	Between individuals within populations	**3.50**	**9.53**	**7.31**	**6.70**	**5.47**	**6.52**	**10.55**	**6.32**
	Within individuals	**69.81**	**67.11**	**75.09**	**78.53**	**65.39**	**76.22**	**75.98**	**68.09**

Genetic variance explained according to (a) subcontinental origin based on separate analyses for wild, captive and combined provenances; (b) population provenance (wild vs. captive) based on separate analyses for each subcontinent, as well as all regions. Significant variance components are highlighted in bold. See also Table S7, Additional file 2

To infer provenance-mediated structure within each subcontinent, we performed separate AMOVAs (Table 3b) and assessed the effects of provenance nested within subcontinents on *A*_*R*_ (Table S8, Additional file 2). We neither found structure (Table 3b) nor significant differences in diversity (Table S8, Additional file 2) between captive and wild populations in South and Central America and Australia, respectively. This suggests that in these geographic regions captive populations exclusively derive from local wild gene pools and that there is frequent gene flow between provenances and hence limited genetic signatures of fly farming practices on local captive populations (Fig. 3, Figure S5, Additional file 2). Conversely, pronounced structure with respect to provenance observed in Europe, North America and Africa and to a lesser extent Asia (Table 3b) suggests that BSF strains used for farming in these regions were mostly derived from a common genetically distinct and exclusively captive origin rather than local wild populations (Figs. 2 and 3, Figure S5, Additional file 2). In Africa and Asia, it appears that both local wild populations and recently introduced strains are used for farming, with occasional admixture between them (Fig. 1, Table 2). For instance, two genetically distinct clades across Asian BSF farms (only one of which matches regional wild populations; Fig. 3, Figure S5, Additional file 2) reflect an intermediate variance structure according to provenance within Asia (Table 3b). While North American wild populations harboured significantly more alleles than captive strains, the reverse was found in Asia (*p* < 0.001; Tables S5 and S8, Additional file 2).

### Population genetic characteristics and colonisation patterns of wild BSF on different subcontinents

**Fig. 4.**
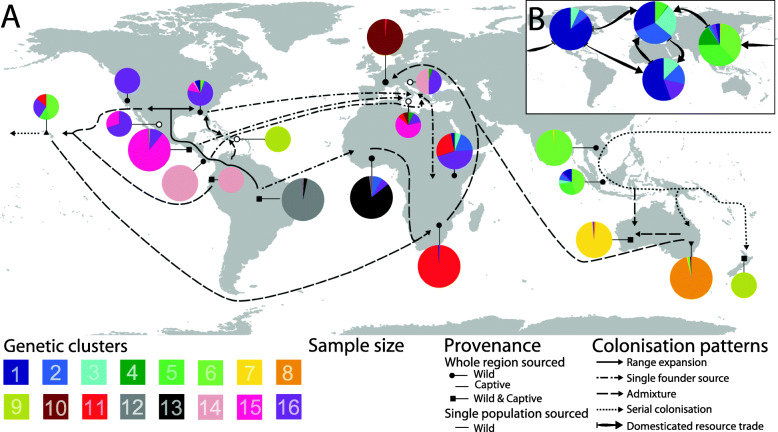
Inferred global distribution dynamics of *Hermetia illucens* genetic clusters. Pie charts represent proportions of individuals sampled in major geographic regions and assigned to genetic clusters according to Fig. 1 (see also Table 2 and Table S6, Additional file 2). Pie diameters correspond to sample sizes and pies comprising multiple samples reflect whole regions (see Table S10, Additional file 2, for more detail). **a** Map depicting biogeographically informative wild and/or captive populations as indicated according to the symbols plotted on starting points referring to major geographic regions. Colonisation routes, as inferred from ABC analyses (Figure S6, Tables S11-13, Additional file 2), are indicated by schematically simplified trajectories. Indigenous range expansions are shown by solid arrows and dispersal to non-native regions are differentiated to show founder events from single sources (dashed-dotted arrows), admixture between demographically independent introductions (dashed arrows), and serial colonisations (dotted arrows). Origins mostly refer to whole regions rather than specific locations. **b** Map depicting exclusively captive population pools from entire continents of North America, Europe, Africa and Asia, and inferred major trading directions of domesticated strains and their introgressants of clusters 1–5 (see Table S10, Additional file 2, and main text for more detail)

To better understand the demographic history of worldwide BSF populations, we investigated geographic characteristics of their population structure and relationships between wild populations within and across subcontinents. We considered captive populations as biogeographically informative whenever they appeared to be wild-derived from local native or naturalised populations: i.e. captive populations from South and Central America, Australia and Asian captive populations assigned to cluster 6 (Figs. 1 and 3, Figure S5, Additional file 2). An overview of the patterns detailed below is provided in Fig. 4, which highlights global cluster occurrences according to geography and provenance as well as putative range expansions and anthropogenic introductions.

#### Americas

Wild populations from North, Central and South America exhibited higher *A*_*R*_ than wild populations from other continents (*p* < 0.01; Table S8, Additional file 2) but did not significantly differ from each other, albeit wild populations from North America harboured substantially fewer alleles (Table S5, Additional file 2). Considering both provenances, South American populations harboured the most private alleles and the highest allelic diversity worldwide, with twelve of 150 samples (7% of all individuals) comprising one third of the globally occurring population-private alleles and 65% of the overall allelic variation (Tables S3 and S5, Additional file 2). Cluster analysis placed central-eastern South American populations in the most diverse cluster 12, while north-western South American populations group with the southern-most Central America populations in the separate cluster 14 (Figs. 3 and 4, Table S6, Additional file 2). Central American populations north of Costa Rica form the well differentiated cluster 15, which is displaced in northern Mexico by the less diverse cluster 16 of North American wild populations (Table 2, Figs. 1 and 4, Table S6, Additional file 2). Central American mainland populations were thus assigned to the three distinct genetic clusters 14, 15 and 16 (Figs. 1 and 3, Table 2), which altogether exhibited far stronger IBD (*r* = 0.476) than South American populations (*r* = 0.149; Table S9, Additional file 2). These patterns indicate a striking north-south gradient of genetic variation in America (Figs. 2 and 4). To test whether this was the result of a historic range expansion, we compared various demographic scenarios with approximate Bayesian computation (ABC) based on coalescent simulations. The best-supported model considers central-east South American cluster 12 as ancestral, and the South American cluster 14 as the source that colonised Central America and gave rise to Central American cluster 15, from which North American populations of cluster 16 most recently derived (posterior probability [*P*] = 0.85; Figure S6-A, Tables S11-13, Additional file 2). The Caribbean sample was distinct from all other American populations (cluster 9 in Figs. 1 and 4, Table 2), and additional ABC-based analyses indicated admixture primarily between populations from south-eastern North America and central-eastern South America (*P* = 0.46; Figure S6-C, Tables S11-13, Additional file 2).

#### Australasia

Asian and Australian naturalised populations are closely related and did not differ in *A*_*R*_, but harboured significantly fewer alleles than American wild populations (*p* < 0.01; Tables S3, S5 and S8, Additional file 2), from which they are distinct (Fig. 2). Naturalised Asian populations formed cluster 6, while eastern and western Australian populations grouped in cluster 8 and the genetically least diverse but highly distinct cluster 7, respectively (Figs. 1 and 4, Table 2, Table S6, Additional file 2). Importantly, separate IBD patterns across Asian and Australian populations remained unchanged when pooled in a joint Australasian group (Table S9, Additional file 2). Individuals from Hawaii were allocated to diverse clusters, while New Zealand populations grouped with Caribbean samples in cluster 9. These Polynesian samples were altogether among the least differentiated compared to populations from other subcontinents (Figs. 3 and 4, Figure S5, Additional file 2). ABC analysis indicated that northern Polynesia is the most likely origin of the colonisation of the entire Australasian region (*P* = 0.77; Figure S6-D, Tables S11-13, Additional file 2). From there, serial colonisations reached out first to Southeast Asia and successively, via the Pacific islands, to eastern Australia. Our analyses indicate western Australian populations originated from admixture between Asian and eastern Australian naturalised lineages (Figure S6-D, Tables S11-13, Additional file 2). ABC analyses further showed that the original colonisation of Polynesia can be best explained by admixture between north-western South American and western North American populations (*P* = 0.50; Figure S6-E, Tables S11-13, Additional file 2).

#### Africa

Allelic richness *A*_*R*_ of African wild populations (Table S8, Additional file 2) was significantly lower than in South and Central America (*p* < 0.05), but not different from North American wild populations. Only wild populations from central-east Africa and two wild populations from west Africa grouped close to captive African populations and North American wild populations (clusters 2 and 16, respectively, Table 2, Figs. 1 and 3). Cluster 13 exclusively comprised wild populations from west Africa (Table 2, Fig. 4). This group was one of the most genetically distinct and least differentiated from South American populations (Figs. 1 and 3, Table S6, Figure S5, Additional file 2). All wild populations from south-east Africa formed cluster 11 (Table 2, Figs. 1 and 4), which appeared the least differentiated from Australasian wild populations of all African wild populations. Indeed, ABC analysis revealed that south-east African populations most likely originated from admixture between west African populations and a subsequent introduction from Polynesia (*P* = 0.50; Figure S6-F, Tables S11-13, Additional file 2). Other demographic models indicate that cluster 11 neither directly descended nor experienced admixture from central-east African populations of cluster 16.

#### Europe

European wild populations exhibited significantly reduced *A*_*R*_ compared to wild populations from the Americas (*p* < 0.001; Table S8, Additional file 2). Cluster analysis assigned all western European wild populations to cluster 10 (Table 2, Fig. 4), related to south-east African and eastern Australian populations (Fig. 1). This pattern was also supported by FCA (Fig. 2), while *D*_*CH*_ grouped all western European wild populations with west African populations (Fig. 3). In line with these results, ABC analyses indicate admixture between south-east African cluster 11 and eastern Australian cluster 8 as the best scenario for the origin of western European populations (*P* = 0.75; Figure S6-G, Tables S11-13, Additional file 2). Consistent clustering with wild populations from the Americas was found in the Mediterranean: the southern European wild population largely grouped with cluster 15, while individuals of the south-eastern European wild population were assigned to cluster 14 or cluster 16 (Table 2, Figs. 1, 3 and 4).

### Origin and population genetic patterns of globally predominant captive populations

The majority of captive populations from North America, Europe, Africa and Asia grouped in clusters 1–4, which exhibited shallow but distinct structure and similar levels of *A*_*R*_ across subcontinents (Table 2, Figs. 1 and 3, Table S6 and S8, Additional file 2). Populations characterised by considerable individual assignments to any cluster of this group also tended to show strong admixture between these (i.e. comparatively lower within-population proportions of single cluster assignment, Fig. 1c), suggesting common exchanges across farms worldwide, as supported by cross-locus inbreeding coefficients *F*_IS_. Populations of clusters 1–4 did not significantly differ from global wild populations, and both groups showed significantly lower *F*_IS_ than captive populations that were locally derived from wild populations of other clusters (Table S14, Additional file 2). Strikingly, however, most of the deviations from linkage equilibrium were detected in captive populations assigned to clusters 1–4, which often exhibited characteristic LD across nine markers for seven of the 105 locus pairs (five to 14 populations per locus pair). Linked pairs of loci were exclusively on the same chromosome, and at large distances of up to 14.9, 48.4 and 20.8 Mbp for chromosomes 2, 3 and 4, respectively. Ratios of the variance components for LD between (*D*_ST_) and within (*D*_IS_) populations [47] across all pairs of loci were significantly lower for populations assigned to clusters 1–4 than for wild populations worldwide, but not compared to wild-derived captive populations from other clusters (Table S15, Additional file 2). Specifically considering the seven pairs of loci in high LD, clusters 1–4 demonstrated significantly lower *D*_ST_/*D*_IS_ ratios than both wild and captive populations in other clusters, but no significant differences were detected between the two latter groups. By contrast, no significant differences among the three groups were found across the remaining 98 pairs of loci.

Captive populations predominating across North America, Europe, Africa and (to a lesser extent) Asia were closely related but markedly distinct from the geographically closest wild populations (Fig. 2, Figure S5, Additional file 2). The closest match of these clusters 1–4 with North American cluster 16 (Fig. 1, Table S6, Additional file 2), particularly wild populations from south-eastern USA (Fig. 3), is in accordance with a priori expectations regarding the geographic origin of globally most widespread captive populations. ABC analyses favoured south-eastern North America as the direct source of North American captive populations in the significantly best-explaining model (*P* = 0.47; Figure S6-B, Tables S11-13, Additional file 2). The latter form a genetically uniform group (Fig. 2) and strongly reduced genetic variance (Table 3b, Tables S3 and S5, Additional file 2) rule out substantial gene exchange with regional wild populations in the recent past. North American captive populations were jointly assigned to cluster 1 (Fig. 1, Table 2), together with several captive populations from Europe and Africa which appear to have been sourced from North American captive populations in recent years, as mostly confirmed by the sample providers. Cluster 2 subsumes mostly European and a few African captive populations (Fig. 1, Table 2). Personal communication with sample providers allowed us to trace cluster 2 back to an earlier introduction of North American captive populations to Europe around 2005, and moreover revealed that populations assigned to exclusively captive clusters 3 and 4 stem from two more recent breeding programmes initiated independently in Europe and Asia (Fig. 1, Table 2).

### Introgression between widely farmed strains and local wild populations

A better understanding of the frequency, extent and directionality of introgression between globally structured wild populations and genetically highly uniform and distinct captive strains is key to evaluate the impact of increasing global trade and large-scale BSF farming on indigenous and naturalised populations. Further, the characterisation of genetic origins of cross-bred captive strains could support future breeding efforts and traceability.

In this context, two west African wild populations assigned to admixed clusters 2 and 16 (Figs. 1 and 3), rather than cluster 13 as expected, were identified as F_1_ hybrids and backcrosses with parental groups represented by west African wild populations and European captive populations (cluster 2) reportedly translocated to a nearby BSF facility 2 years prior (Figure S7, Additional file 2).

Similarly, ancestry coefficients revealed that wild and captive populations from central-east Africa that were allocated to the North American cluster 16 were hybrids between parental groups originating from south-east African (cluster 11) and regionally abundant captive populations (clusters 1–3) (Figure S8, Additional file 2), with extensive backcross re-assignments documenting vast admixture across provenances in this region.

Lastly, hybrids between Asian naturalised populations (cluster 6) and captive populations of North American origin (clusters 1–3) were identified in Asian clusters 4 and 5 (Table 2, Figure S8, Additional file 2). While cluster 5 was inferred to be evenly admixed, including backcrosses in both directions, cluster 4 appears only marginally introgressed, with very limited genetic signatures of Asian wild populations.

Several wild populations sampled across considerable distances in Africa demonstrate introgression from modern BSF farms and research facilities (Figs. 1c and 3), indicating that hybrid populations have established repeatedly in nature in this region (Fig. 4). By contrast, all but one of the hybrid populations in Asia were captive, suggesting that more recently introduced farmed BSF have caused limited, merely local introgression into Asian wild populations. Instead, the close relatedness among Asian hybrid populations (Figs. 2 and 3) points to very few independent recent hybridisation events, followed by frequent subsequent transfers of up to millions of individuals each across Asian and European farms (the latter being confirmed by sample providers).

Interestingly, recent human-mediated admixture between distantly related clusters contributed to increased genetic variance between individuals within populations, as exemplified for Asian populations (Table 3b), rather than the expected increase of variance within individuals that was typically found in American wild populations. These patterns coincide with disproportionally strong deviations from HWE (Table S3, Additional file 2) across hybrid populations between captive North American and naturalised Asian or (to a lesser extent) African origins.

## Discussion

Our comprehensive population genetic study of wild and captive populations of BSF on a global scale using highly discriminating microsatellites permitted a fundamental genetic characterisation of this commercially important insect. Our data provided novel insights into (1) the geographic distribution of genetic variation and population structure of wild and captive populations; (2) the origin of specific colonisations and general patterns of range expansions across the world, including basic delimitations of historic vs. contemporary events; and (3) the genetic relationships between wild and captive populations, which exhibit genetic footprints of domestication, and local gene flow between the two.

### Indigenous genetic hotspots, reconstruction of worldwide dispersal routes, and admixture as a trigger of rapid non-native range expansion

Worldwide patterns of nuclear genetic diversity are consistent with the previously presumed origin of BSF in the Americas [39, 45]. In accordance with the centre of species diversity of the genus *Hermetia* [40], South America was identified as a BSF genetic hotspot and the cradle of a complex, presumably ancient dispersal history across the Americas, and more recently around the world (Fig. 4). We provide evidence that central-east South American BSF are ancestral, while Central America was colonised from already derived source populations from north-western South America west of the Andes. Spatial bottlenecks and climate shifts may induce drift and adaptive processes, respectively [48]. Both could be mirrored in the pronounced population structure characterising the Central American range expansion of BSF - by the isthmus of Panama and more arid regions in northern Mexico, before their dispersal into North America (Figs. 3 and 4), which was last colonised within the Americas. These inferences are corroborated by the highly characteristic mitochondrial genetic structure of North American BSF [45]. Nevertheless, south-east North American wild populations were least differentiated in conjunction with signals of admixture in Caribbean individuals (Figs. 3 and 4, Figure S5, Additional file 2), suggesting occasional gene flow from South America into south-east North America via the Caribbean islands.

Reduced genetic diversity in Asia and Australia indicates BSF is not native to these regions. Characteristic genetic signatures and IBD patterns of naturalised BSF populations across the entire Australasian region suggest a single successful colonisation event followed by successive range expansion, resulting in a unique population structure (Fig. 1, Table 2). Polynesian populations compellingly trace back to a primary admixture event between distantly related lineages from the American West Coast (Figs. 3 and 4). The inferred colonisation route of Australasia via serial introductions coupled with moderate genetic drift (Fig. 4) conforms to BSF documentation records [39] and identifies admixed Polynesian founders as bridgehead populations [49–52]. Western Australia, which was most recently colonised, harbours the most differentiated genetic cluster (Figs. 1 and 4, Table S6, Additional file 2). Its origin from secondary contact between previously split Southeast Asian and eastern Australian lineages can explain several private alleles that might have ‘surfed’ at lacing edges of the Australasian range expansion [48, 50].

We infer that Africa was colonised via three independent demographic events in different parts of the continent (Fig. 4). Consistent with the phylogeographic signal of the mitochondrial COI marker [45], our nuclear genetic data indicate an exclusive South American origin of west African cluster 13. The south-east African cluster 11 showed admixed ancestry between west African and Polynesian origins (Figs. 1 and 4), which was unexpected as first reports of BSF in South Africa are older than those from Hawaii. This implies potential documentation gaps regarding an earlier colonisation across Pacific islands, and/or a change in the originally colonised areas of pure cluster 13, which today is found only in west African refuges and whose current genetic profile could have been shaped in the course of geographical shifts. Patterns observed in central-eastern Africa reflect progressing introgression between south-east African wild populations and more recently introduced captive populations that are used in several large-scale farming facilities across the continent. However, an additional independent introduction of North American wild populations of cluster 16 to central-eastern Africa cannot be ruled out (Figs. 3 and 4) and is indeed supported by shared mitochondrial haplotypes between wild samples from Kenya as well as Oklahoma and Florida, USA [45].

European wild populations are all highly distinct from European captive populations and appear to stem from at least three independent introductions from the Americas plus a fourth lineage that was the result of a remarkable admixture event. Clusters 15 and 14 from Central and South America, which dominated southern and south-eastern European populations, respectively, were otherwise not detected outside their indigenous ranges and are thus both considered unique introductions (Fig. 4). Both Mediterranean populations also featured assignments to cluster 16, which suggests gene flow among them, and implies a third colonisation either from North American wild populations directly, via potential ongoing range expansions from central-east Africa into the Mediterranean, or vice versa (Fig. 4). Moreover, despite their geographic proximity, ABC analyses indicate that neither Mediterranean population appears to have given rise to wild populations of western Europe (cluster 10 in Figs. 1 and 4). Admixed ancestry of the latter between two demographically distinct descendants of the primarily admixed Polynesian bridgehead agrees with BSF documentations from central Europe, which are younger than those from the Mediterranean as well as those from eastern Australia and south-east Africa.

Our data conclude that BSF became naturalised virtually everywhere outside its native range in the Americas. Inadvertent anthropogenic introductions from the Americas before the 19th century via historic shipping routes (e.g. in organic cargo or waste) would be plausible for all non-native regions. Not only in the Mediterranean, where our finding of a Central American origin supports previous speculations on local BSF occurrence by the 16th century [53], but also across Polynesia and West Africa. Nevertheless, assuming the earliest verified documentation dating back to the 1920s–1950s in Africa, Europe, Asia and Australia reflect the true onsets of wider successful colonisations, our data imply that BSF accomplished cosmopolitan range expansions during the last century at the same rate as *Harmonia axyridis*, *Drosophila suzukii*, *Aedes albopictus* or *Anoplophora glabripennis* [51, 54–57]. In several contemporary examples of invasive insects, it has been demonstrated that decisive bridgehead effects involving intraspecific admixture were the trigger for rapid large-scale range expansions across non-native areas, irrespective of occasional older, locally more restricted single-source colonisations [50–52, 54, 55, 58]. Admixture between differentiated lineages is supposed to increase genetic variation and generate novel genotype combinations for selection to act on [49, 59, 60]. Naturalised populations in south-east Africa and western Europe both independently trace back to multiple consecutive admixture events, with common origins in the primarily admixed bridgehead that previously initiated the Australasian invasion-hub (Fig. 4). Thus, our finding that the largest non-native areas were invaded by admixed populations, while only a limited number of the BSF colonisations traced back to single founder events deriving from native American sources directly, such as west and central-east African and Mediterranean populations, represents a compelling example in favour of this hypothesis. Our observation coincides with previous evidence that two distantly related COI haplotypes prevail across entire Australasia, which are neither abundant in indigenous ranges nor present in common captive populations outside this region [45]. Yet, one of them is indeed uniquely shared among wild populations from western Europe and south-east Africa [45].

After initial establishment (with or without admixture), human activity could have mediated numerous unintended secondary translocations within non-native continents that may have accelerated range expansions even prior to the recent advent of BSF farming. However, long-distance dispersal of initial colonisers did not disturb pronounced IBD patterns of naturalised populations at large and medium geographic scales (Table S9, Additional file 2), nor did independent transcontinental introductions break up the apparent genetic distinctiveness of founder populations by generating detectable substructure patches of diversity [48], with the exception of the serial colonisation of Australasia as a whole (Figs. 3 and 4). Therefore, the lack of noteworthy wider geographic substructure beyond IBD within each of these non-native regions indicates that unique admixture events upon independent introductions preceded successful range expansions, which were not substantially affected by genetic signatures of potential subsequent demographic effects [61]. Both genetic drift and ecological adaptation (see below) may have contributed to highly distinct genetic profiles of BSF across Australasia, south-east Africa and western Europe, and most likely occurred during lag phases and prior to rapid invasions [50, 62, 63]. Thus, BSF dispersal in most non-native areas likely followed a fast and continuous ‘wave-of-advance’ scenario [46, 48]. This may reflect a species-specific dispersal strategy that was most likely initiated by humans but subsequently only passively promoted by them through widespread availability of suitable breeding habitats for this opportunistic synanthropic fly [61]. Surprisingly, little is known about natural BSF dispersal, but a presumed bivoltine lifecycle in most climates combined with high fecundity may predispose BSF for quick dispersal, even to areas characterised by only seasonally suitable habitats.

### Demography and genetic signatures of domesticated BSF

Clusters 1–5 comprise the majority of captive populations used for commercial operations and academic research worldwide. The complementary analyses detailed above indicate they represent direct descendants (or introgressants) originating from a single source in south-eastern North America that were spread successively across other continents, notably Europe, Africa and Asia. This conclusion is supported by a recent phylogeographic study based on mitochondrial COI markers [45]. This founding stock most likely goes back to an establishment of approximately 20,000 wild-collected pupae in the course of some early work in 1998 in Alma, Georgia, USA [44, J.K. Tomberlin, personal communication] (i.e. almost 200 generations ago, considering 8–9 generations per year under suitable captive conditions).

North American captive populations of cluster 1 (Fig. 1, Table 2) harbour low microsatellite allelic diversity (Table S5, Additional file 2), indicating that their genetic signatures were shaped by a pronounced founder effect, coupled with strong artificial selection in isolation (e.g. [64]). An early division of this captive North American gene pool, represented by cluster 2, features more diverse mitochondrial haplotypes also found in North American wild populations [45]. This may be owing to introgression from wild populations in Europe (or Africa) shortly after its introduction there around 2005, and/or stronger drift across North American captive populations during the last decade. Interestingly, the opposite extremes of two more recent breeding approaches generated only modest, albeit detectable, genetic signatures (Fig. 1, Table 2): cluster 3, derived from a European breeding programme, should have experienced even more pronounced drift, while cluster 4 demonstrably involved outcrossing with Asian wild populations prior to subsequent selection. Nevertheless, similar genetic footprints of the original North American strain are seen worldwide.

Strikingly elevated levels of LD of seven specific locus pairs that are characteristic of the closely related clusters 1–4 further support their shared demographic origin [65]. Such genetic signatures often result from domestication processes and breed formation [66, 67]. However, it remains difficult to distinguish population contractions from selection due to similar genetic patterns [68, 69]. At first glance, the specific associations of our microsatellites suggest long-range physical linkage due to a severe bottleneck, which may only slowly decay via recombination in isolation [65]. Nevertheless, descendants of cluster 1 that experienced different introgression fates on different continents (i.e. clusters 2 and 4) still prominently expressed these specific LD patterns, whereas they were absent across other populations, although *D*-statistics suggested that wild-derived captive populations assigned to other clusters may have been similarly affected by genome-wide demographic effects. Thus, the maintenance (or restoration) of genetic signatures characteristic of the original North American strain potentially reflects common selective pressures in modern BSF farming that counter the decay of inter-chromosomal long-distance LD [70]. We therefore propose that the specific genetic footprints of clusters 1–4 were not only shaped by demographic history but may also reflect past and ongoing selective adaptation to artificial breeding in different genomic regions and are indicative of domestication in BSF. However, further research with high-resolution genome-wide data is necessary to quantitatively test this hypothesis.

Moreover, despite substantially decreased genetic diversity compared to native wild populations, severe inbreeding of the domesticated populations was not indicated. The maintenance of moderate genetic diversity in domesticated strains may indicate past [46, 71] and/or ongoing admixture [72]. Assuming current admixture takes place primarily among captive populations (Fig. 1c), its influence on increasing diversity may be limited compared to its impact on counterbalancing diversifying breed formation. Yet, the absence of severe genetic bottlenecks across BSF farms utilising domesticated strains, even after isolation for more than a decade (as per several captive populations investigated), may also point at mechanisms stabilising genetic diversity in artificial BSF regimes, as shown for other domesticated animals and insects [69, 73, 74]. Recurrent outcrossing to counteract inbreeding does therefore not appear necessary when rearing BSF populations continuously in captivity at sufficiently large effective population sizes.

### Impact and limitations of introgression between wild and domesticated BSF in an evolutionary ecology context

Gene flow between provenances, or the lack thereof despite opportunities, allows interesting insights into the direct competitiveness of domesticated strains and wild populations in artificial settings and natural habitats. Most populations showing introgression between domesticated and wild origins were kept in captivity in non-native regions, particularly in Asia and Africa (Fig. 4, Figures. S7 and S8, Additional file 2). This suggests that certain traits of local wild populations may be superior [63] in a regional BSF farming context and thus facilitate directional gene flow into recently introduced domesticated strains. Conversely and counter to expectations [52], nuclear gene flow into local wild populations was not widespread in non-native areas, where domesticated strains of North American origin are extensively used for farming (notably Africa, Asia, and Europe). Although the locally strong extent of introgression across central-east Africa and in different settings in west Africa and Southeast Asia indicates that domesticated BSF strains are not necessarily maladapted to field conditions per se (e.g. [59, 75]), most regionally naturalised populations still seem quite resilient. This can hardly be explained by estimated effective population sizes *N*_e_, which varied across populations but were well comparable between provenances in all regions and even tended to be lower for wild populations outside South and Central America (Tables S3 and S5, Additional file 2), and probably only to a limited extent by precautionary measures to prevent escapes with regard to ubiquitous semi-open farms [76]. Instead, biological mechanisms might limit introgression from recently introduced domesticated strains into local wild populations. Various factors may be operating whose causes and consequences deserve future investigation. Disproportionally strong deviations from HWE in BSF hybrid populations from Asia and Europe (Table S3, Additional file 2) may suggest a tendency towards assortative mating (as shown in other insects, e.g. [77]), or a form of outbreeding depression via breakdown of beneficial epistatic interactions [78, 79] that could have particularly evolved in domesticated strains. Alternatively, manipulative maternally inherited endosymbiont bacteria may maintain cryptic population substructure, if penetrance was weak or variable [80, 81]. Such mechanisms could explain both the absence of admixture between two distinct clusters within a single wild population in the putative contact zone in south-eastern Europe (Fig. 1c and 4), and a notable mito-nuclear discordance of captive Asian hybrid populations despite the lack of fundamental genetic incompatibilities, as implied by comparison with a previous mitochondrial phylogeography [45]. Another factor may relate to the ability to diapause during unfavourable periods (e.g. winter or dry seasons) [82–84]. Diapause in BSF has been investigated only superficially [85], but it is likely that wild populations of cooler regions (e.g. Europe) have evolved vital adaptions to seasonally adverse cold periods [86–88].

As in other insects [89, 90], ecological selection likely shaped, and continues to shape, the evolutionary trajectories of BSF, not only in their indigenous ranges in the Americas. Phenotypically plastic species, such as BSF, are particularly well equipped for invasive range expansions, potentially resulting in changes in genetic variances of colonising populations that ultimately confer to local genetic adaptations in adventive environments via increased heritability of fitness-related traits [52, 59, 62, 79, 91]. Presumably, several phenotypic responses to environmental factors have a genetic basis in BSF, such as in traits like larval body composition or life-history characteristics [33, 34]. This might similarly apply to putative co-evolution of BSF with their associated microbes [92, 93], as known from other insect systems [94, 95]. Uniquely different genetic signatures in virtually all colonised non-native areas, including both diverse admixed origins and single-source descendants in more specific allopatric refuges, likely reflect adaptation of biotypes to novel regional ecological niches [59]. This further sheds a uniquely nuanced light on the interplay between putative adaptive evolution of generally increased invasiveness conferred to primarily admixed bridgehead populations and differential ecological selection on their secondarily admixed descendants in newly colonised regions [58]. Advantageous alleles in any regionally naturalised BSF population that are superior to the alleles present in common domesticated strains may improve breeding efforts for local small-holder BSF farming [96]. Beyond that, BSF populations exhibiting unique traits conducive to particular large-scale BSF farming conditions or purposes worldwide may be locally present but remain unexplored.

At present, many ecologically viable local BSF populations appear hitherto ignored by worldwide BSF farms. The widely encountered genetic uniformity across captive populations on several continents may reflect that domesticated strains largely outcompete other, non-adapted BSF genotypes in artificial farming environments. Alternatively, it may simply reflect a convenience-mediated, generally misguided tendency to overly focus on genetically uniform populations in research surrounding commercial biological applications [75, 97]. Reinforced introgression from domesticated strains may emerge more frequently around BSF farms and research facilities [76], thereby disrupting local genetic (co)adaptations of wild populations and thus posing a threat to native but also unique naturalised populations. An anthopogenically induced establishment of more competitive introgressed domesticated strains in natural habitats could further affect the propensity of invasiveness to new as well as already populated ranges [51, 52, 98]. While safeguarding natural BSF genetic resources outside indigenous ranges may not represent a conservational concern at first sight, the loss of either native or naturalised locally unique genetic integrity through introgression, as documented in other systems [99], would immediately jeopardise explorations for their future commercial use.

## Conclusions

Besides its major role in increasing agronomic sustainability globally, the BSF model has huge potential to provide significant conceptual advances in understanding the interplay between genetic differentiation, organismal dispersal and formation of geographic lineages. This species appears highly amenable for investigating rapid divergence and local adaptation in the framework of ecological genetics of invasive insects. Future research on how the previously unrecognised ample genetic variation affects life-history, physiological and behavioural traits will further our understanding of causal mechanisms underlying domestication processes in animals. Fast and reliable assessment of genetic differences beyond the primarily used commercial BSF strains will become increasingly important. Based on the characterisation of all genetic clusters and their origins presented here, and in light of rapidly developing genomic resources [35, 36], we highlight that the genetic make-up of BSF populations used for commercial purposes or academic research will need to be taken into account in future research. The presented microsatellite markers provide a simple, robust and cost-efficient molecular tool kit, which allows for comparative integration into future samples relative to our comprehensive global dataset. This benchmark of the worldwide BSF population genetic inventory will foster guided surveys of wild and mass-reared captive BSF populations, investigations of gene-by-environment interactions, explorations of phenotypic trait architectures and future breeding efforts to harness the potential of this insect for tackling socio-economic challenges. If BSF farming indeed becomes an increasingly important and expanding economic endeavour, akin to conventional livestock, more differentiated strategies for the use and breeding of BSF populations are recommended. Awareness of the vast genetic diversity of BSF, its substantially structured global genetic architecture, and the availability of a variety of unique regional gene pools around the globe represents an advanced basis for future developments.

## Methods

### Marker development, fragment amplification and analysis

The development of novel microsatellite markers was commissioned to *ecogenics* (Balgach, Switzerland) and based on a single female from the breeding population at the Research Institute of Organic Agriculture (FiBL), Switzerland, which was reared isolated in captivity for about one decade. The library was analysed on an Illumina MiSeq platform using the Nano 2 × 250 v2 format. Microsatellite inserts with a tetra- or a trinucleotide motif of at least 6 repeat units or a dinucleotide motif of at least 10 repeat units were available in 2856 assembled contigs or singlets. Suitable primer design was possible for 2228 microsatellite candidates. Based on an initial screening of specimens deriving from few selected populations supposedly reflecting the species’ global distribution range, fifteen di- and trinucleotide microsatellite candidates, which were polymorphic and amplified without null alleles, were selected for optimising our multiplex PCR protocols (Table S2, Additional file 2).

Genomic DNA was extracted from adult thorax muscle or from larval heads including one or two (depending on size) larval segments. Tissue was ground in 250 μL of 5% Chelex solution (50–100 mesh; Sigma Aldrich, St. Louis, USA), incubated at 65 °C for 15 min, boiled at 98 °C for 10 min and then centrifuged at 13,000 rpm for 3 min before transferring the supernatant. Three multiplexed PCRs with five markers each were developed, considering dye combinations based on fragment size ranges (Table S2, Additional file 2) and using the same cycling conditions: after an initial denaturation of 15 min at 95 °C, 35 cycles of 30 s at 94 °C, 90 s at 56 °C and 60 s at 72 °C were performed and completed by a final extension step at 72 °C for 30 min. Multiplex PCRs were carried out on GeneAmp PCR System 9700 and Bio Rad S1000 thermal cyclers in 5 μL total reaction volume. Single reactions contained 1× PCR-Buffer (10× Qiagen, Hilden, Germany), 0.2 mM of each dNTP, 0.05 U μL^−1^ HotStarTaq (Qiagen), 1 μL (approximately 3–5 ng, based on Qubit analyses) genomic DNA and 0.2–0.9 μM of both forward and reverse primers for each marker. Total primer concentrations as well as specific ratios of labelled and unlabelled primers (Microsynth, Balgach, Switzerland) were adjusted according to relative marker-specific amplification effectiveness within optimised multiplex reactions as specified in Table S2, Additional file 2. Fragment sizes were determined on an ABI 3730 automated sequencer in relation to an internal size standard (GeneScan-500 LIZ, Thermo Fisher Scientific, Warrington, UK). Allele scoring was performed using the Genemapper software v. 5.0 (Thermo Fisher Scientific).

### Sampling

Samples from 150 sampling populations in 57 countries were gathered during 2017–2019. Populations were grouped according to subcontinent of origin, i.e. South America, Central America (including the Caribbean), North America, Africa, Asia (including Sunda Islands), Australia (including New Zealand/Polynesia) and Europe (see also Table S4, Additional file 2, for countries of origin). Additionally, populations were categorised as wild or captive (referred to as provenance status). Specifically, all populations reared in captivity (for academic research, commercial farming or hobby purposes) for more than one generation prior to collection were considered captive, irrespective of possible or even intended exchange with local wild populations, for example in semi-open facilities. Field-collected samples are supposed to represent random subsamples of local populations; however, some samples may have comprised biases in time (discrete generations of adults and larvae collected at the same time) or space (pooling of otherwise too few specimens from several spots within a wider vicinity). We targeted 20 individuals per population, yet eight populations included less than 10 and four populations included more than 30 specimens (see Table S3, Additional file 2). For overall grouping and illustration consistency, but also because several providers of commercially used population samples wished to stay anonymous, information on geographic sample origins more detailed than at the subcontinental level are referred to only selectively whenever relevant (particularly for wild populations, see Table S1, Additional file 2).

### Data analyses

Data were analysed with R v. 3.6.1 [100], unless stated otherwise. Potentially matching multilocus genotypes (MLGs) and discriminatory power of the applied markers based on 10,000 re-sampling steps were assessed with the package *poppr* v. 2.8.3 [101, 102]. Locus-, population- and group-specific numbers of alleles were calculated using the packages *adegenet* v. 2.1.1 [103, 104] and *hierfstat* v. 0.04-22 [105]. Indications of significant null allele frequencies (Brookfield 2 method), and allelic richness (*A*_R_), rarefied to the lowest population sample size (i.e. five diploid individuals), were evaluated using the package *PopGenReport* v. 3.0.4 [106, 107]. *A*_R_ was further analysed with linear mixed models [108] using the package *lme4* v. 1.1-21 [109]. Locus was included as a random effect in three independent models including population subcontinental origin, population provenance status and provenance nested within subcontinents, respectively, as fixed effects. Model fits were evaluated by means of likelihood ratio tests, model assumptions were met and significance was tested using post hoc Tukey contrasts of the package *multcomp* v. 1.4-10 [110]. Overall, locus- and population-specific *F*-statistics (*F*_ST_ and *F*_IS_), observed and expected heterozygosity, as well as tests for deviations from Hardy-Weinberg-Equilibrium (HWE) within populations were computed and visualised using *adegent, hierfstat*, *pegas* v. 0.12 [111], *strataG* v. 2.0.2 [112] and *ggplot* v. 3.2.1 [113]. Cross-locus population-specific *F*_IS_ were subjected to weighted linear regression for specific population groups of interest, with weights being set as the inverse of the residual variances of the response to account for lower variances of global wild populations, complemented by significance testing using Tukey contrasts. The software *Arlequin* v. 3.5.2.2 [114] was used for assessing linkage disequilibrium (LD) between locus pairs within populations and respective significance testing based on 1000 permutations. Variance components of pairwise marker linkage within and between populations based on the *D*-statistics according to Ohta [45] were computed using *GENETIX* v. 4.05.2 [115]. Ratios of *D*_ST_/*D*_IS_ were log-transformed for variance stabilisation and subjected to linear mixed models, including locus pair as random effect and population groups of interest (i.e. wild, wild-derived captive and domesticated captive) as fixed effect, and respective post hoc Tukey contrasts between fixed effects.

Alpha levels or confidence limits for assessing locus-specific (or pairwise) deviations from HWE and LD, as well as significant estimates of null allele frequencies, were adjusted based on the number of tests within single populations (not globally across populations, which might have been too conservative). Likewise, confidence limits for tests of population-specific *F*_IS_ were adjusted based on the total number of populations, not accounting for tests across loci within populations.

Analysis of molecular variance (AMOVA) was performed using *poppr* and subjected to significance testing as implemented in the package *ade4* v. 1.7-13 [116–119] based on 99 permutations. Hierarchical evaluations based on original sampling populations were progressively run for continental levels (separate AMOVAs for the entire data, as well as wild and captive populations) and provenance levels (separate AMOVAs for the entire data, as well as for each individual subcontinent), and finally for the complete dataset by nesting provenance within subcontinents.

Population structure was also explored through pairwise *F*_ST_ and corresponding significance testing (based on 10,000 permutations), as provided by *strataG*. For pairwise *F*_ST_ tests, *α* was arbitrarily set to 0.0001 as the maximum conservative adjustment for that number of permutations. The resulting genetic distance matrix was visualised as an unrooted neighbour-joining tree using the package *Ape* [120]. An additional neighbour-joining dendrogram based on Cavalli-Sforza and Edwards chord distances (*D*_CH_, [121]) between populations was constructed using *poppr*.

Isolation by distance (IBD) patterns was evaluated using Mantel tests [122] based on 9999 permutations using the package *vegan* v. 2.5-6 [123]. Specifically, we compared matrices of linearised genetic differentiation (*F*_ST_ / (1 − *F*_ST_), and log-transformed geographic distances (in kilometres) between populations [124]. Based on sampling location coordinates (latitude, longitude; data not shown) obtained from public resources, pairwise geographic distances were generated from spatial points and transformed into Euclidean distances using the packages *sp* v. 1.3-1 [125, 126] and *SciViews* v. 13.1 [127]. In addition to analysing the overall sample, separate analyses were run for all wild and all captive samples, as well as for all subcontinents, each with and without inclusion of captive populations.

We used the *snapclust* function of *adegenet*, which provides a rapidly converging maximum-likelihood solution by combining a geometric approach and the Expectation-Maximisation algorithm, to infer the optimal number of genetic clusters (*K*) in the data by applying KIC goodness-of-fit statistics for model selection [128, 129], see Figure S3 A (Additional file 2). We visualised individuals’ posterior membership probabilities by stacked bar plots using *ggplot*. Discriminant analysis of principal components was applied to depict genetic structure across individual MLGs based on cluster assignment (or sampling populations) as implemented in *adegenet*. This multivariate method focuses on variances between groups while minimising within group variation, and it characterises population subdivision with similar accuracy but faster than common Bayesian clustering algorithms [130]. Retained principal components were cross-validated as detailed in Figures S3 B-C (Additional file 2) to avoid overfitting. To visualise overall genetic structure based on population ‘barycentres’ in a multidimensional space, factorial correspondence analysis (FCA) using *GENETIX* v. 4.05.2 was conducted.

To infer regional hybridisation and introgression based on estimates of ancestry coefficients, specifically suspected populations (or entire clusters) of interest and respectively suggested parental groups were subjected to dedicated analyses of F_1_ hybrid (0.5:0.5) and first generation backcross (0.25:0.75 or 0.75:0.25) detection, using the *snapclust* function as implemented in *adegenet* [128].

Effective population sizes (*N*_e_) were calculated using the LD test, considering a mating system equivalent to lifetime monogamy and conservatively excluding all singleton alleles within populations, in *NeEstimator v.2.1* [131].

The population genetics analyses of microsatellite data from our worldwide BSF samples revealed complex clustering with respect to geography. In several cases, classical population genetic approaches did not allow to distinguish competing hypotheses about the demographic history of related populations. We therefore employed Approximate Bayesian Computation (ABC) based on coalescent simulations as implemented in DIYABC (v.2.1; [132]) to compare the probability of competing demographic models. In total, we analysed seven specific demographic problems, which include the prospective origin of BSF, the colonisation histories of different continents with and without admixture events among independent introductions and the origin of widespread captive populations (see Table S11, Additional file 2). For each of these seven analyses, we built reference tables including various numbers of demographic models with uniform prior distributions for each parameter, and picked representative population samples based on our a priori genetic clustering approach (Table S11, Additional file 2).

To compare observed and simulated data, we used the allelic information of all 15 microsatellite loci (see Table S16, Additional file 2, for motif lengths and ranges) and chose a generalised stepwise mutation model (GSM) with mean mutation rates (*μ*) drawn from a uniform prior distribution ranging from 10^−5^ to 10^−3^. For the coefficient *P* (the parameter of the geometric distribution describing the length variation of microsatellite loci) and mean single-nucleotide indel (SNI) mutation rate, we used uniform prior distributions ranging from 0.1 to 0.99 and from 10^−8^ to 10^−5^, respectively. We further chose a combination of three one-sample summary statistics that included mean number of alleles, mean genetic diversity [133] and mean size variance, as well as seven two-sample summary statistics, including mean number of alleles, mean genetic diversity, mean size variance, shared allelic distance [134], mean index of classification [135], genetic differentiation (*F*_ST_; [136]) and genetic distance ((δμ)^2^; [137]). For each of the seven analyses, we generated one million simulated datasets per demographic model. Using principal component analyses (PCA) and comparisons of observed and simulated summary statistics, we pre-evaluated models and parameter priors to test if these were suitable for subsequent analyses. Then, we computed posterior probabilities of each model using weighted polychotomous logistic regression based on the components of linear discriminant analyses (LDA) from logit-transformed summary statistics of the 1% simulated datasets that were most similar to the observed data. We considered the best-fitting model significant if the 95% confidence intervals did not overlap with the second-best model (see Table S12 and Figure S6, Additional file 2). For each significant model, we further estimated posterior distributions of population genetic parameters using the default settings of DIYABC (Table S13, Additional file 2).

## Supplementary Information

**Additional file 1: Table S1**. Allelic states at 15 novel microsatellite markers for 2862 unique multilocus genotypes of the black soldier fly, *Hermetia illucens*, from 150 wild and captive populations sampled from 57 countries on seven subcontinents.

**Additional file 2: Table S2**. Microsatellite marker characteristics of the novel *Hermetia illucens* population genetics tool kit. **Table S3**. Population genetic characteristics of 150 wild and captive *Hermetia illucens* populations. **Table S4**. Countries of origin of investigated *Hermetia illucens* populations. **Table S5**. Population genetic characteristics according to subcontinental origin and provenance status (wild vs. captive). **Table S6**. Diversity and pairwise differentiation of globally inferred genetic clusters of *Hermetia illucens*. **Table S7**. AMOVA nesting provenance (wild vs. captive) within subcontinents for worldwide *Hermetia illucens* populations. **Table S8**. Model-based estimates of contrasts and significance levels for population-specific allelic richness. **Table S9**. Genetic isolation by distance for selected hierarchical groupings. **Table S10**. Specifications on the global distribution of *Hermetia illucens* genetic clusters presented in Fig. 4. **Table S11**. Details on ABC analyses. **Table S12**. Posterior probabilities of demographic models inferred from ABC analyses. **Table S13**. Estimates of posterior distributions of population genetic parameters inferred from ABC analyses. **Table S14**. Selected group comparisons of inbreeding coefficients *F*_IS_. **Table S15**. Group-specific comparisons of variance component ratios for linkage disequilibrium between (*D*_ST_) and within (*D*_IS_) populations. **Table S16**. Microsatellite properties relevant for ABC analyses. **Figure S1**. Discriminatory power of the novel microsatellite marker set for *Hermetia illucens* genotyping. **Figure S2**. Significant deviations from Hardy-Weinberg equilibrium for individual microsatellite loci tested within populations. **Figure S3**. Computational details on genetic cluster analyses and retaining discriminatory functions for visualisation. **Figure S4**. Global population genetic patterns of *Hermetia illucens* according to provenance (wild vs. captive) nested within subcontinent of origin. **Figure S5**. Neighbour-joining tree based on population pairwise *F*_ST_ across 150 *Hermetia illucens* populations. **Figure S6**. Demographic inference with ABC. **Figure S7**. Detection of hybrids and backcrosses: a west African case of introgression. **Figure S8**. Detection of hybrids and backcrosses: case-specific analyses of central-east African populations and predominantly farmed populations from Asia.

## Data Availability

All data generated or analysed during this study are included in this published article and its supplementary information files, e.g. all genotyping raw data are provided in Supplementary Table S1, Additional file 1, and GenBank accession numbers of sequences of each locus (microsatellite motifs and flanking regions including primers) are available in Supplementary Table S2, Additional file 2.

## References

[1] van Huis A (2013). Potential of insects as food and feed in assuring food security. Annu Rev Entomol..

[2] Makkar HPS, Tran G, Henze V, Ankers P (2014). State-of-the-art on use of insects as animal feed. Anim Feed Sci Tech..

[3] Kupferschmidt K (2015). Buzz Food. Science.

[4] ČiČková H, Newton GL, Lacy RC, Kozánek M (2015). The use of fly larvae for organic waste treatment. Waste Manage..

[5] Alexander P, Brown C, Arneth A, Finnigan J, Moran D, Rounsevell MDA (2017). Losses, inefficiencies and waste in the global food system. Agric Syst..

[6] Muller A, Schader C, El-Hage Scialabba N, Bruggemann J, Isensee A, Erb KH, Smith P, Klocke P, Leiber F, Stolze M (2017). Strategies for feeding the world more sustainably with organic agriculture. Nat Commun..

[7] Pelletier N, Tyedmers P (2010). Forecasting potential global environmental costs of livestock production 2000-2050. P Natl Acad Sci..

[8] Schader C, Muller A, Scialabba Nel H, Hecht J, Isensee A, Erb KH, Smith P, Makkar HP, Klocke P, Leiber F (2015). Impacts of feeding less food-competing feedstuffs to livestock on global food system sustainability. J R Soc Interface..

[9] Cashion T, Tyedmers P, Parker RWR (2017). Global reduction fisheries and their products in the context of sustainable limits. Fish and Fisheries..

[10] Oonincx DGAB, van Itterbeeck J, Heetkamp MJW, van den Brand H, van Loon JJA, van Huis A (2010). An exploration on greenhouse gas and ammonia production by insect species suitable for animal or human consumption. Plos One.

[11] Bosch G, van Zanten HHE, Zamprogna A, Veenenbos M, Meijer NP, van der Fels-Klerx HJ, van Loon JJA (2019). Conversion of organic resources by black soldier fly larvae: legislation, efficiency and environmental impact. J Clean Prod..

[12] Gasco L, Biasato I, Dabbou S, Schiavone A, Gai F (2019). Animals fed insect-based diets: state-of-the-art on digestibility, performance and product quality. Animals..

[13] Smetana S, Schmitt E, Mathys A (2019). Sustainable use of *Hermetia illucens* insect biomass for feed and food: attributional and consequential life cycle assessment. Resour Conservcycl..

[14] Tomberlin JK, van Huis A (2020). Black soldier fly from pest to ‘crown jewel’ of the insects as feed industry: an historical perspective. J Ins Food Feed..

[15] Nguyen T-X, Tomberlin J, Vanlaerhoven S (2015). Ability of black soldier fly (Diptera: Stratiomyidae) larvae to recycle food waste. Environ Entomol..

[16] Jucker C, Erba D, Leonardi MG, Lupi D, Savoldelli S (2017). Assessment of vegetable and fruit substrates as potential rearing media for *Hermetia illucens* (Diptera: Stratiomyidae) Larvae. Environ Entomol..

[17] Ewusie EA, Kwapong PK, Ofosu-Budu G, Sandrock C, Akumah A, Nartey E, Teye-Gaga C, Agyarkwah SK, Adamtey N (2018). Development of black soldier fly, *Hermetia illucens* (Diptera: Stratiomyidae) in selected organic market waste fractions in Accra, Ghana. Asian J Biotechnol Bioresour Technol..

[18] Lalander C, Diener S, Zurbrügg C, Vinnerås B (2019). Effects of feedstock on larval development and process efficiency in waste treatment with black soldier fly (*Hermetia illucens*). J Clean Prod..

[19] Oonincx DGAB, van Huis A, van Loon JJA (2015). Nutrient utilisation by black soldier flies fed with chicken, pig, or cow manure. J Ins Food Feed.

[20] Oonincx DGAB, van Broekhoven S, van Huis A, van Loon JJA (2015). Feed conversion, survival and development, and composition of four insect species on diets composed of food by-products. Plos One.

[21] Rehman KU, Cai MM, Xiao XP, Zheng LY, Wang H, Soomro AA, Zhou YS, Li W, Yu ZN, Zhang JB (2017). Cellulose decomposition and larval biomass production from the co-digestion of dairy manure and chicken manure by mini-livestock (*Hermetia illucens* L.). J Environ Manage..

[22] Barragan-Fonseca KB, Dicke M, van Loon JJA (2017). Nutritional value of the black soldier fly (*Hermetia illucens* L.) and its suitability as animal feed - a review. J Ins Food Feed..

[23] Spranghers T, Ottoboni M, Klootwijk C, Ovyn A, Deboosere S, De Meulenaer B, Michiels J, Eeckhout M, De Clercq P, De Smet S (2017). Nutritional composition of black soldier fly (*Hermetia illucens*) prepupae reared on different organic waste substrates. J Sci Food Agr..

[24] Heuel M, Sandrock C, Leiber F, Mathys A, Gold M, Zurbrügg C, Gangnat IDM, Kreuzer M, Terranova M (2021). Black soldier fly larvae meal and fat can completely replace soybean cake and oil in diets for laying hens. Poult. Sci..

[25] Dabbou S, Gai F, Biasato I, Capucchio MT, Biasibetti E, Dezzutto D, Meneguz M, Placha I, Gasco L, Schiavone A (2018). Black soldier fly defatted meal as a dietary protein source for broiler chickens: effects on growth performance, blood traits, gut morphology and histological features. J Anim Sci Biotechno..

[26] Neumann C, Velten S, Liebert F (2018). N Balance studies emphasize the superior protein quality of pig diets at high inclusion level of algae meal (*Spirulina platensis*) or insect meal (*Hermetia illucens*) when adequate amino acid supplementation is ensured. Animals..

[27] Biasato I, Renna M, Gai F, Dabbou S, Meneguz M, Perona G, Martinez S, Lajusticia ACB, Bergagna S, Sardi L, Capucchio MT, Bressan E, Dama A, Schiavone A, Gasco L (2019). Partially defatted black soldier fly larva meal inclusion in piglet diets: effects on the growth performance, nutrient digestibility, blood profile, gut morphology and histological features. J Anim Sci Biotechno..

[28] Stadtlander T, Stamer A, Buser A, Wohlfahrt J, Leiber F, Sandrock C (2017). *Hermetia illucens* meal as fish meal replacement for rainbow trout on farm. J Ins Food Feed..

[29] Nogales-Merida S, Gobbi P, Jozefiak D, Mazurkiewicz J, Dudek K, Rawski M, Kieronczyk B, Jozefiak A (2019). Insect meals in fish nutrition. Rev Aquacult..

[30] Leong SY, Kutty SRM, Malakahmad A, Tan CK (2016). Feasibility study of biodiesel production using lipids of *Hermetia illucens* larva fed with organic waste. Waste Manage..

[31] Surendra KC, Olivier R, Tomberlin JK, Jha R, Khanal SK (2016). Bioconversion of organic wastes into biodiesel and animal feed via insect farming. Renew Energ..

[32] Jensen K, Kristensen T, Heckmann L-H, Sørensen J, van Huis A, Tomberlin JK (2017). Breeding and maintaining high-quality insects. Insects as food and feed: from production to consumption.

[33] Zhou F, Tomberlin JK, Zheng LY, Yu ZN, Zhang JB (2013). Developmental and waste reduction plasticity of three black soldier fly strains (Diptera: Stratiomyidae) raised on different livestock manures. J Med Entomol.

[34] Sandrock C, Leupi S, Wohlfahrt J, Leiber F, Kreuzer M (2019). Genotype × environment interactions in black soldier fly larvae grown on different feed substrates. 70th Annual Meeting of the European Federation of Animal Science.

[35] Zhan S, Fang G, Cai M, Kou Z, Xu J, Cao Y, Bai L, Zhang Y, Jiang Y, Luo X, Xu J, Xu X, Zheng L, Yu Z, Yang H, Zhang Z, Wang S, Tomberlin JK, Zhang J, Huang Y (2020). Genomic landscape and genetic manipulation of the black soldier fly *Hermetia illucens*, a natural waste recycler. Cell Res..

[36] Generalovic TN, McCarthy SA, Warren IA, Wood JMD, Torrance J, Sims Y, et al. A high-quality, chromosome-level genome assembly of the Black Soldier Fly (*Hermetia Illucens* L.). G3. 2021 (advance article online access). 10.1093/g3journal/jkab085. 10.1093/g3journal/jkab085 PMC8104945 33734373

[37] Lessard BD, Yeates DK, Woodley NE (2019). Revision of the Hermetiinae of Australia (Diptera: Stratiomyidae). Austral Entomol..

[38] Rozkosný R. A biosystematic study of the European Stratiomyidae (Diptera). In: Spencer, KA, editor. Clitellariinae, Hermetiinae, Pachygasterinae and Bibliography. Series Entomol. 1983;2:172-176.

[39] Marshall SA, Woodley NE, Hauser M (2015). The historical spread of the black soldier fly, *Hermetia illucens* (L.) (Diptera, Stratiomyidae, Hermetiinae), and its establishment in Canada. J Ent Soc Ont..

[40] Woodley NE (2001). A World Catalog of the Stratiomyidae (Insecta: Diptera). Int J North Am Dipterists’ Soc..

[41] Hardy DE (1960). Insects of Hawaii. Volume 10, Diptera: Nematocera-Brachycera.

[42] Leclercq M (1997). A propos de *Hermetia illucens* (LINNAEUS, 1758) (“soldier fly”) (Diptera: Stratiomyidae: Hermetiinae). Bull Annls Soc r belge Ent..

[43] Booth DC, Sheppard C (1984). Oviposition of the black soldier fly, *Hermetia illucens* (Diptera, Stratiomyidae) - eggs, masses, timing, and site characteristics. Environ Entomol..

[44] Sheppard DC, Tomberlin JK, Joyce JA, Kiser BC, Sumner SM (2002). Rearing methods for the black soldier fly (Diptera: Stratiomyidae). J Med Entomol..

[45] Ståhls G, Meier R, Sandrock C, Hauser M, Šašić Zorić L, Laiho E, Aracil A, Doderović J, Badenhorst R, Unadirekkul P, Mohd Adom NAB, Wein L, Richards C, Tomberlin JK, Rojo S, Veselić S, Parviainen T (2020). The puzzling mitochondrial phylogeography of the black soldier fly (*Hermetia illucens*), the commercially most important insect protein species. BMC Evol Biol..

[46] Larson G, Burger J (2013). A population genetics view of animal domestication. Trends Genet..

[47] Ohta T (1982). Linkage disequilibrium due to random genetic drift in finite subdivided populations. P Natl Acad Sci..

[48] Excoffier L, Foll M, Petit RJ (2009). Genetic consequences of range expansions. Annu Rev Ecol Evol Syst..

[49] Estoup A, Guillemaud T (2010). Reconstructing routes of invasion using genetic data: why, how and so what?. Mol Ecol..

[50] Lawson Handley LJ, Estoup A, Evans DM, Thomas CE, Lombaert E, Facon B, Aebi A, Roy HE (2011). Ecological genetics of invasive alien species. BioControl..

[51] Lombaert E, Guillemaud T, Thomas CE, Lawson Handley LJ, Li J, Wang S, Pang H, Goryacheva I, Zakharov IA, Jousselin E (2011). Inferring the origin of populations introduced from a genetically structured native range by approximate Bayesian computation: case study of the invasive ladybird *Harmonia axyridis*. Mol Ecol..

[52] Garnas JR, Auger-Rozenberg M-A, Roques A, Bertelsmeier C, Wingfield MJ, Saccaggi DL, Roy HE, Slippers B (2016). Complex patterns of global spread in invasive insects: eco-evolutionary and management consequences. Biol Invasions..

[53] Benelli G, Canale A, Raspi A, Fornaciari G (2014). The death scenario of an Italian Renaissance princess can shed light on a zoological dilemma: did the black soldier fly reach Europe with Columbus?. J Archaeol Sci..

[54] Fraimout A, Debat V, Fellous S, Hufbauer RA, Foucaud J, Pudlo P, Marin J-M, Price DK, Cattel J, Chen X, Deprá M, François Duyck P, Guedot C, Kenis M, Kimura MT, Loeb G, Loiseau A, Martinez-Sañudo I, Pascual M, Polihronakis Richmond M, Shearer P, Singh N, Tamura K, Xuéreb A, Zhang J, Estoup A (2017). Deciphering the routes of invasion of *Drosophila suzukii* by means of ABC random forest. Mol Biol Evol..

[55] Javal M, Lombaert E, Tsykun T, Courtin C, Kerdelhue C, Prospero S, Roques A, Roux G (2019). Deciphering the worldwide invasion of the Asian long-horned beetle: a recurrent invasion process from the native area together with a bridgehead effect. Mol Ecol..

[56] Adrion JR, Kousathanas A, Pascual M, Burrack HJ, Haddad NM, Bergland AO, Machado H, Sackton TB, Schlenke TA, Watada M, Wegmann D, Singh ND (2014). *Drosophila suzukii*: the genetic footprint of a recent, worldwide invasion. Mol Biol Evol..

[57] Goubert C, Minard G, Vieira C, Boulesteix M (2016). Population genetics of the Asian tiger mosquito *Aedes albopictus*, an invasive vector of human diseases. Heredity..

[58] Bertelsmeier C, Keller L (2018). Bridgehead effects and role of adaptive evolution in invasive populations. Trends Ecol Evol..

[59] Estoup A, Ravigné V, Hufbauer R, Vitalis R, Gautier M, Facon B (2016). Is there a genetic paradox of biological invasion?. Annu Rev Ecol Evol Syst..

[60] Rius M, Darling JA (2014). How important is intraspecific genetic admixture to the success of colonising populations?. Trends Ecol Evol..

[61] Wilson JRU, Dormontt EE, Prentis PJ, Lowe AJ, Richardson DM (2009). Something in the way you move: dispersal pathways affect invasion success. Trends Ecol Evol..

[62] Bock DG, Caseys C, Cousens RD, Hahn MA, Heredia SM, Hübner S, Turner KG, Whitney KD, Rieseberg LH (2015). What we still don’t know about invasion genetics. Mol Ecol..

[63] Facon B, Hufbauer Ruth A, Tayeh A, Loiseau A, Lombaert E, Vitalis R, Guillemaud T, Lundgren Jonathan G, Estoup A (2011). Inbreeding depression is purged in the invasive insect *Harmonia axyridis*. Curr Biol..

[64] Rhode C, Badenhorst R, Hull K, Greenwood M, Bester A, Andere A, Picard CJ, Richards C (2020). Genetic and phenotypic consequences of early domestication in black soldier flies (*Hermetia illucens*). Anim Genet..

[65] Slatkin M (2008). Linkage disequilibrium- understanding the evolutionary past and mapping the medical future. Nat Rev Genet..

[66] Gray MM, Granka JM, Bustamante CD, Sutter NB, Boyko AR, Zhu L, Ostrander EA, Wayne RK (2009). Linkage disequilibrium and demographic history of wild and domestic canids. Genetics.

[67] Rossi M, Bitocchi E, Bellucci E, Nanni L, Rau D, Attene G, Papa R (2009). Linkage disequilibrium and population structure in wild and domesticated populations of *Phaseolus vulgaris* L. Evol Appl..

[68] Thornton KR, Jensen JD, Becquet C, Andolfatto P (2007). Progress and prospects in mapping recent selection in the genome. Heredity.

[69] Wiener P, Wilkinson S (2011). Deciphering the genetic basis of animal domestication. Proc R Soc B..

[70] Dale-Kuys RC, Roodt-Wilding R, Rhode C (2020). Genome-wide linkage disequilibrium in South African abalone, *Haliotis midae*, and implications for understanding complex traits. Aquaculture.

[71] Makino T, Rubin CJ, Carneiro M, Axelsson E, Andersson L, Webster MT (2018). Elevated proportions of deleterious genetic variation in domestic animals and plants. Genome Biol Evol..

[72] Harpur BA, SM, Kent CF, Zayed A (2012). Management increases genetic diversity of honey bees via admixture. Mol Ecol..

[73] Burke MK, Dunham JP, Shahrestani P, Thornton KR, Rose MR, Long AD (2010). Genome-wide analysis of a long-term evolution experiment with *Drosophila*. Nature..

[74] Zygouridis NE, Argov Y, Nemny-Lavy E, Augustinos AA, Nestel D, Mathiopoulos KD (2014). Genetic changes during laboratory domestication of an olive fly SIT strain. J Appl Entomol..

[75] Tayeh A, Estoup A, Laugier G, Loiseau A, Turgeon J, Toepfer S, Facon B (2012). Evolution in biocontrol strains: insight from the harlequin ladybird *Harmonia axyridis*. Evol Appl..

[76] Bang A, Courchamp F (2021). Industrial rearing of edible insects could be a major source of new biological invasions. Ecol Lett..

[77] Su W, Michaud JP, Runzhi Z, Fan Z, Shuang L (2009). Seasonal cycles of assortative mating and reproductive behaviour in polymorphic populations of *Harmonia axyridis* in China. Ecol Entomol..

[78] Kawecki TJ, Ebert D (2004). Conceptual issues in local adaptation. Ecol Lett..

[79] Dlugosch KM, Anderson SR, Braasch J, Cang FA, Gillette HD (2015). The devil is in the details: genetic variation in introduced populations and its contributions to invasion. Mol Ecol..

[80] Cooper BS, Ginsberg PS, Turelli M, Matute DR (2017). Wolbachia in the *Drosophila yakuba* complex: pervasive frequency variation and weak cytoplasmic incompatibility, but no apparent effect on reproductive isolation. Genetics..

[81] Layton EM, On J, Perlmutter JI, Bordenstein SR, Shropshire JD (2019). Paternal grandmother age affects the strength of *Wolbachia*-induced cytoplasmic incompatibility in *Drosophila melanogaster*. mBio.

[82] Lehmann P, Lyytinen A, Piiroinen S, Lindström L (2015). Latitudinal differences in diapause related to photoperiodic responses of European Colorado potato beetles (*Leptinotarsa decemlineata*). Evol Ecol..

[83] Ragland GJ, Armbruster PA, Meuti ME (2019). Evolutionary and functional genetics of insect diapause: a call for greater integration. Curr Opin Insect Sci.

[84] Zeender V, Roy J, Wegmann A, Schäfer MA, Gourgoulianni N, Blanckenhorn WU, Rohner PT (2019). Comparative reproductive dormancy differentiation in European black scavenger flies (Diptera: Sepsidae). Oecologia..

[85] Samayoa AC, Hwang SY (2018). Degradation capacity and diapause effects on oviposition of *Hermetia illucens* (Diptera: Stratiomyidae). J Econ Entomol..

[86] Holmes LA, VanLaerhoven SL, Tomberlin JK (2016). Lower temperature threshold of black soldier fly (Diptera: Stratiomyidae) development. J Ins Food Feed..

[87] Spranghers T, Noyez A, Schildermans K, De Clercq P (2017). Cold Hardiness of the black soldier fly (Diptera: Stratiomyidae). J Econ Entomol..

[88] Chia SY, Tanga CM, Khamis FM, Mohamed SA, Salifu D, Sevgan S, Fiaboe KKM, Niassy S, van Loon JJA, Dicke M, Ekesi S (2018). Threshold temperatures and thermal requirements of black soldier fly *Hermetia illucens*: Implications for mass production. Plos One..

[89] Sandrock C, Schirrmeister BE, Vorburger C (2011). Evolution of reproductive mode variation and host associations in a sexual-asexual complex of aphid parasitoids. BMC Evol Biol..

[90] Kapun M, Barrón MG, Staubach F, Obbard DJ, Wiberg RAW, Vieira J, Goubert C, Rota-Stabelli O, Kankare M, Bogaerts-Márquez M, Haudry A, Waidele L, Kozeretska I, Pasyukova EG, Loeschcke V, Pascual M, Vieira CP, Serga S, Montchamp-Moreau C, Abbott J, Gibert P, Porcelli D, Posnien N, Sánchez-Gracia A, Grath S, Sucena É, Bergland AO, Guerreiro MPG, onder BS, Argyridou E, Guio L, Schou MF, Deplancke B, Vieira C, Ritchie MG, Zwaan BJ, Tauber E, Orengo DJ, Puerma E, Aguadé M, Schmidt P, Parsch J, Betancourt AJ, Flatt T, González J (2020). Genomic analysis of European *Drosophila melanogaster* populations reveals longitudinal structure, continent-wide selection, and previously unknown DNA viruses. Mol Biol Evol..

[91] Blanckenhorn WU. Causes and consequences of phenotypic plasticity in body size: the case of the yellow dung fly *Scathophaga stercoraria* (Diptera: Scathophagidae). In: Whitman DW, Ananthakrishnan TN, editors. Phenotypic plasticity of insects: mechanisms and consequences. Enfield: Science Publishers, Inc.; 2009. p. 369-422, doi: 10.1201/b10201-11.

[92] Wynants E, Frooninckx L, Crauwels S, Verreth C, De Smet J, Sandrock C, Wohlfahrt J, Van Schelt J, Depraetere S, Lievens B (2019). Assessing the microbiota of black soldier fly larvae (*Hermetia illucens*) reared on organic waste streams on four different locations at laboratory and large scale. Microb Ecol..

[93] Khamis FM, Ombura FLO, Akutse KS, Subramanian S, Mohamed SA, Fiaboe KKM, Saijuntha W, Van Loon JJA, Dicke M, Dubois T (2020). Insights in the global genetics and gut microbiome of black soldier fly, *Hermetia illucens*: implications for animal feed safety control. Front Microbiol.

[94] Vorburger C, Sandrock C, Gouskov A, Castañeda L, Ferrari J (2009). Genotypic variation and the role of defensive endosymbionts in an all-parthenogenetic host-parasitoid interaction. Evolution..

[95] Wang Y, Kapun M, Waidele L, Kuenzel S, Bergland AO, Staubach F (2020). Common structuring principles of the *Drosophila melanogaster* microbiome on a continental scale and between host and substrate. Environ Microbiol Rep..

[96] Ewusie EA, Kwapong PK, Ofosu-Budu G, Sandrock C, Akumah AM, Nartey EK, Tetegaga C, Agyakwah SK (2019). The black soldier fly, *Hermetia illucens* (Diptera:Stratiomyidae): trapping and culturing of wild colonies in Ghana. Sci Afr..

[97] Suurväli J, Whiteley AR, Zheng Y, Gharbi K, Leptin M, Wiehe T (2020). The laboratory domestication of zebrafish: from diverse populations to inbred substrains. Mol Biol Evol..

[98] Hufbauer RA, Facon B, Ravigné V, Turgeon J, Foucaud J, Lee CE, Rey O, Estoup A (2012). Anthropogenically induced adaptation to invade (AIAI): contemporary adaptation to human-altered habitats within the native range can promote invasions. Evol Appl..

[99] Brede N, Sandrock C, Straile D, Spaak P, Jankowski T, Streit B, Schwenk K (2009). The impact of human-made ecological changes on the genetic architecture of *Daphnia* species. P Natl Acad Sci..

[100] R Core Team (2019). R: a language and environment for statistical computing.

[101] Kamvar ZN, Tabima JF, Grünwald NJ (2014). Poppr: an R package for genetic analysis of populations with clonal, partially clonal, and/or sexual reproduction. PeerJ..

[102] Kamvar ZN, Brooks JC, Grünwald NJ (2015). Novel R tools for analysis of genome-wide population genetic data with emphasis on clonality. Front Genet..

[103] Jombart T (2008). adegenet: a R package for the multivariate analysis of genetic markers. Bioinformatics..

[104] Jombart T, Ahmed I (2011). adegenet 1.3-1: new tools for the analysis of genome-wide SNP data. Bioinformatics..

[105] Goudet J, Jombart T. hierfstat: estimation and tests of hierarchical F-statistics. R package version 0.04-22. 2015. https://CRAN.R-project.org/package=hierfstat. Accessed 28 Feb 2020.

[106] Adamack AT, Gruber B (2014). PopGenReport: simplifying basic population genetic analyses in R. Methods Ecol Evol..

[107] Gruber B, Adamack AT (2015). Landgenreport: a new R function to simplify landscape genetic analysis using resistance surface layers. Mol Ecol Resour..

[108] Soro A, Quezada-Euan JJG, Theodorou P, Moritz RFA, Paxton RJ (2017). The population genetics of two orchid bees suggests high dispersal, low diploid male production and only an effect of island isolation in lowering genetic diverstiy. Conserv Genet..

[109] Bates D, Maechler M, Bolker B, Walker S (2015). Fitting linear mixed-effects models using lme4. J Stat Softw..

[110] Hothorn T, Bretz F, Westfall P (2008). Simultaneous inference in general parametric models. Biom J..

[111] Paradis E (2010). pegas: an R package for population genetics with an integrated-modular approach. Bioinformatics..

[112] Archer FI, Adams PE, Schneiders BB (2017). strataG: an R package for manipulating, summarizing and analysing population genetic data. Mol Ecol Resour..

[113] Wickham H (2016). ggplot2: elegant graphics for data analysis.

[114] Excoffier L, Laval G, Schneider S (2005). Arlequin ver. 3.0: an integrated software package for population genetics data analysis. Evol Bioinform Online..

[115] Belkhir K, Borsa P, Chiki L, Raufaste N, Bonhomme F. GENETIX, logiciel sous WindowsTM pour la génétique des populations. Montpellier: Laboratoire Génome, Populations, Interactions CNRS UMR 5000, Université de Montpellier II. 1996–2004.

[116] Chessel D, Dufour A, Thioulouse J (2004). The ade4 Package - I: One-Table Methods. R News..

[117] Dray S, Dufour A (2007). The ade4 package: implementing the duality diagram for ecologists. J Stat Softw..

[118] Dray S, Dufour A, Chessel D (2007). The ade4 package - II: two-table and K-table methods. R News..

[119] Bougeard S, Dray S (2018). Supervised multiblock analysis in R with the ade4 Package. J Stat Softw..

[120] Paradis E, Schliep K (2018). ape 5.0: an environment for modern phylogenetics and evolutionary analyses in R. Bioinformatics..

[121] Cavalli-Sforza LL, Edwards AW (1967). Phylogenetic analysis. Models and estimation procedures. Am J Hum Genet..

[122] Mantel N (1967). The detection of disease clustering and a generalized regression approach. Cancer Res..

[123] Oksanen J, Blanchet FG, Friendly M, Kindt R, Legendre P, McGlinn D, Minchin PR, O'Hara RB, Simpson GL, Solymos P, et al. vegan: Community Ecology Package. 2019. https://CRAN.R-project.org/package=vegan. Accessed 14 April 2020.

[124] Rousset F (1997). Genetic differentiation and estimation of gene flow from F-statistics under isolation by distance. Genetics..

[125] Pebesma EJ, Bivand RS (2005). Classes and methods for spatial data in R. R News..

[126] Bivand RS, Pebesma E, Gomez-Rubio V (2013). Applied spatial data analysis with R.

[127] Grosjean P (2019). SciViews-R.

[128] Beugin MP, Gayet T, Pontier D, Devillard S, Jombart TA-O (2018). A fast likelihood solution to the genetic clustering problem. Methods Ecol Evol..

[129] Akogul S, Erisoglu M (2016). A comparison of information criteria in clustering based on mixture of multivariate normal distributions. Math Comput Appl..

[130] Jombart T, Devillard S, Balloux F (2010). Discriminant analysis of principal components: a new method for the analysis of genetically structured populations. BMC Genet..

[131] Do C, Waples RS, Peel D, Macbeth GM, Tillett BJ, Ovenden JR (2014). NeEstimator v2: re-implementation of software for the estimation of contemporary effective population size (*N*_e_) from genetic data. Mol Ecol Resour..

[132] Cornuet J-M, Pudlo P, Veyssier J, Dehne-Garcia A, Gautier M, Leblois R, Marin J-M, Estoup A (2014). DIYABC v2.0: a software to make approximate Bayesian computation inferences about population history using single nucleotide polymorphism, DNA sequence and microsatellite data. Bioinformatics..

[133] Nei M (1978). Estimation of average heterozygosity and genetic distance from a small number of individuals. Genetics..

[134] Chakraborty R, Jin L, Pena SDJ, Chakraborty R, Epplen JT, Jeffreys AJ (1993). A unified approach to study hypervariable polymorphisms: Statistical considerations of determining relatedness and population distances. DNA Fingerprinting: State of the Science.

[135] Rannala B, Mountain JL (1997). Detecting immigration by using multilocus genotypes. Proc Natl Acad Sci..

[136] Weir BS, Cockerham CC (1984). Estimating F-statistics for the analysis of population structure. Evolution..

[137] Goldstein DB, Ruiz Linares A, Cavalli-Sforza LL, Feldman MW (1995). Genetic absolute dating based on microsatellites and the origin of modern humans. Proc Natl Acad Sci..

