# Supplementary material for: Global population genetic structure and demographic trajectories of the black soldier fly, Hermetia illucens
Source: BMC Biol. 2021 May 5;19:94. doi: 10.1186/s12915-021-01029-w (PMC8101212; doi:10.1186/s12915-021-01029-w)
Supplement: Supplementary file 2 — Additional file 2: Table S2. Microsatellite marker characteristics of the novel Hermetia illucens population genetics tool kit. Table S3. Population genetic characteristics of 150 wild and captive Hermetia illucens populations. Table S4. Countries of origin of investigated Hermetia illucens populations. Table S5. Population genetic characteristics according to subcontinental origin and provenance status (wild vs. captive). Table S6. Diversity and pairwise differentiation of globally inferred genetic clusters of Hermetia illucens. Table S7. AMOVA nesting provenance (wild vs. captive) within subcontinents for worldwide Hermetia illucens populations. Table S8. Model-based estimates of contrasts and significance levels for population-specific allelic richness. Table S9. Genetic isolation by distance for selected hierarchical groupings. Table S10. Specifications on the global distribution of Hermetia illucens genetic clusters presented in Fig. 4. Table S11. Details on ABC analyses. Table S12. Posterior probabilities of demographic models inferred from ABC analyses. Table S13. Estimates of posterior distributions of population genetic parameters inferred from ABC analyses. Table S14. Selected group comparisons of inbreeding coefficients FIS. Table S15. Group-specific comparisons of variance component ratios for linkage disequilibrium between (DST) and within (DIS) populations. Table S16. Microsatellite properties relevant for ABC analyses. Figure S1. Discriminatory power of the novel microsatellite marker set for Hermetia illucens genotyping. Figure S2. Significant deviations from Hardy-Weinberg equilibrium for individual microsatellite loci tested within populations. Figure S3. Computational details on genetic cluster analyses and retaining discriminatory functions for visualisation. Figure S4. Global population genetic patterns of Hermetia illucens according to provenance (wild vs. captive) nested within subcontinent of origin. Figure S5. Neighbour-joining tree based on [file 12915_2021_1029_MOESM2_ESM.pdf]

Supplementary Information for

Global population genetic structure and demographic trajectories of the black soldier fly, *Hermetia illucens*

Cengiz Kaya, Tomas N. Generalovic, Gunilla Ståhls, Martin Hauser, Ana C. Samayoa, Carlos G. Nunes-Silva, Heather Roxburgh, Jens Wohlfahrt, Ebenezer A. Ewusie, Marc Kenis, Yupa Hanboonsong, Jesus Orozco, Nancy Carrejo, Satoshi Nakamura, Laura Gasco, Santos Rojo, Chrysantus M. Tanga, Rudolf Meier, Clint Rhode, Christine J. Picard, Chris D. Jiggins, Florian Leiber, Jeffery K. Tomberlin, Martin Hasselmann, Wolf U. Blanckenhorn, Martin Kapun, Christoph Sandrock

Corresponding author:
Christoph Sandrock, Research Institute of Organic Agriculture (FiBL),
Department of Livestock Sciences, Ackerstrasse 113, 5070 Frick, Switzerland;
email: christoph.sandrock@fibl.org

Additional file 2 - Table of contents

Table S2. Microsatellite marker characteristics of the novel *Hermetia illucens* population genetics tool kit. 3

Table S3. Population genetic characteristics of 150 wild and captive *Hermetia illucens* populations. 4

Table S4. Countries of origin of investigated *Hermetia illucens* populations. 7

Table S5. Population genetic characteristics according to subcontinental origin and provenance status (wild vs. captive). 8

Table S6. Diversity and pairwise differentiation of globally inferred *Hermetia illucens* genetic clusters. 10

Table S7. AMOVA nesting provenance (wild vs. captive) within subcontinents for worldwide *Hermetia illucens* populations. 12

Table S8. Model-based estimates of contrasts and significance levels for population-specific allelic richness. 13

Table S9. Genetic isolation by distance for selected hierarchical groupings. 15

Table S10. Specifications on the global distribution of *Hermetia illucens* genetic clusters presented in Figure 4. 16

Table S11. Details on ABC analyses. 18

Table S12. Posterior probabilities of demographic models inferred from ABC analyses. 20

Table S13. Estimates of posterior distributions of population genetic parameters inferred from ABC analyses. 23

Table S14. Selected group comparisons of inbreeding coefficients F_{IS}. 27

Table S15. Group-specific comparisons of variance component ratios for linkage disequilibrium between (D_{ST}) and within (D_{IS}) populations. 27

Table S16. Microsatellite properties relevant for ABC analyses. 28

Figure S1: Discriminatory power of the novel microsatellite marker set for *Hermetia illucens* genotyping. 29

Figure S2. Significant deviations from Hardy-Weinberg equilibrium of individual microsatellite loci tested within populations. 30

Figure S3. Computational details on genetic cluster analyses and retaining discriminatory functions for visualisation. 31

Figure S4. Global population genetic patterns of *Hermetia illucens* according to provenance (wild vs. captive) nested within subcontinent of origin. 32

Figure S5. Neighbour-joining tree based on population pairwise F_{ST} across 150 *Hermetia illucens* populations. 34

Figure S6. Demographic inference with ABC. 35

Figure S7. Detection of hybrids and backcrosses: a west African case of introgression. 37

Figure S8. Detection of hybrids and backcrosses: case-specific analyses of central-east African populations and predominantly farmed populations from Asia. 38

Table S2. Microsatellite marker characteristics of the novel *Hermetia illucens* population genetics tool kit.

GenBank Accession numbers and primer sequences (F: forward; R: reverse) for the 15 new loci, including superscript numbers for individual dyes (^{1}: FAM; ^{2}: ATTO532; ^{3}: ATTO550; ^{4}: ATTO565) used for forward primer labelling. Total primer concentrations in final PCR reaction volumes are specified (the same for forward and reverse), as are effective concentrations of labelled primers. Repeat motifs, including number of arrays for the sequenced specimen, are listed for each locus, together with documented ranges of fragment lengths in the entire dataset. Effective multiplexed combinations (MP 1 - 3) are indicated but can easily be customised due to identical cycling conditions. Standard quality assurance measures ensure the repeatability of unambiguous allele scoring across different laboratories.

Locus	GenBank accesion number	Primer sequences 5'- 3', dyes and concentrations in PCRs	Repeat array	Size range (bp)	MP
Hi_1-1	MT882382	F^{3}: AGGTCCAGACGGCATTACTC<br>R: GCACGCGCGGTCTATATTC<br>total 0.3 μM; labelled 0.03 μM	$(CTA)_8$	73-141	1
Hi_1-2	MT882383	F^{1}: TTGCATGGTAAGCCTTTGCG<br>R: AACGCGCTACGTACAAATGG<br>total 0.9 μM; labelled 0.9 μM	$(AT)_{13}$	126-206	1
Hi_1-3	MT882384	F^{4}: AGCTGTAGCTTCTCCCCAC<br>R: ACAGCAACATTTCGACACCG<br>total 0.3 μM; labelled 0.03 μM	$(CA)_{13}$	109-315	1
Hi_1-4	MT882385	F^{2}: TTCCGCTTGGAGGATTTTGC<br>R: GCAAATTTTACCACTTCCAAAGTAAAG<br>total 0.2 μM; labelled 0.2 μM	$(TA)_{14}$	142-299	1
Hi_1-5	MT882386	F^{3}: CGCGCTTCCGACATGAAC<br>R: CCCGAATTTCATACGTCGGC<br>total 0.3 μM; labelled 0.03 μM	$(TAC)_8$	181-232	1
Hi_2-1	MT882387	F^{2}: TATCTGGGGCGTTGTTGTTG<br>R: AGTATGATTCAGTGTAGGCAAGG<br>total 0.3 μM; labelled 0.012 μM	$(TGT)_7$	95-123	2
Hi_2-2	MT882388	F^{3}: TCGGTCTTCAGGATCGTCTC<br>R: CAGCCAGTCTCCAATGGTTC<br>total 0.6 μM; labelled 0.2 μM	$(TA)_{18}$	142-254	2
Hi_2-3	MT882389	F^{1}: CAGCCTGGAGGACAGATGAG<br>R: GACTGCTTCTGACGCTTCTAC<br>total 0.3 μM; labelled 0.3 μM	$(TAC)_{10}$	188-230	2
Hi_2-4	MT882390	F^{4}: CGCGACCGTCAACTTCATAG<br>R: AACGGGTTGTCGTATGTGTG<br>total 0.6 μM; labelled 0.6 μM	$(TA)_{14}$	188-224	2
Hi_2-5	MT882391	F^{2}: TGATACCCAGAAGCCCCATC<br>R: CTGTCGCACGCTACAACTTC<br>total 0.25 μM; labelled 0.025 μM	$(CAT)_7$	204-225	2
Hi_3-1	MT882392	F^{1}: CTTATTGGGACGGCCACTTG<br>R: TGCCCTTTGTACACAACTTACG<br>total 0.2 μM; labelled 0.2 μM	$(ACT)_8$	72-174	3
Hi_3-2	MT882393	F^{4}: GCGAGCCGAGAGTGAAAATC<br>R: AGGTCTAGTGGGATGCCAAG<br>total 0.3 μM; labelled 0.03 μM	$(AG)_{12}$	153-200	3
Hi_3-3	MT882394	F^{2}: TGGGGTAGATTGTAGCACACC<br>R: GTGGCAGTTAAAGGACCTCG<br>total 0.3 μM; labelled 0.03 μM	$(AT)_{13}$	161-220	3
Hi_3-4	MT882395	F^{3}: ACAAATGACGTGCCTGATTG<br>R: TGAATATACGACTGCCTGCG<br>total 0.9 μM; labelled 0.09 μM	$(AT)_{15}$	126-214	3
Hi_3-5	MT882396	F^{1}: TCTTTCTAGCCGGAACTTTGG<br>R: GACTTAATCGGCTGGAGCAC<br>total 0.9 μM; labelled 0.9 μM	$(AT)_{12}$	223-241	3

Table S3. Population genetic characteristics of 150 wild and captive *Hermetia illucens* populations.

Populations are ordered according to subcontinents of origin (AF: Africa; AS: Asia; AU: Australia, including Polynesia; CA: Central America, including the Caribbean; EU: Europe; NA: North America; SA: South America). Population provenance is indicated, i.e. wild-sourced (w) versus captive (c), as are the numbers of successfully genotyped specimens (N_I) per population. No*A*: total number of alleles detected within populations; A_R: mean allelic richness per locus, not rarefied; A_{RR}: mean allelic richness per locus rarefied to 5 diploid individual; sum A_R: sum of allelic richness across loci, rarefied to 5 diploid individuals; A_U: proportion of unique alleles, i.e. alleles detected only once; A_P: private alleles, i.e. number of alleles exclusively detected in respective populations; H_O: observed heterozygosity; H_E: expected heterozygosity; F_{IS}: inbreeding coefficient across loci within populations, with significant homozygote excess across loci indicated in bold (the adjusted α-level was set to 0.00033 to account for 150 tested populations, while no global adjustment was applied); *Ne*: inferred effective population size based on the linkage disequilibrium method.

Population	Provenance	N_I	No*A*	A_R	A_{RR}	sum A_R	A_U	A_P	H_O	H_E	F_{IS}	N_e
AF-01	c	50	55	3.67	2.63	39.39	0.07		0.528	0.516	-0.013	147.3
AF-02	w	20	62	4.13	2.99	44.86	0.15	2	0.520	0.549	0.079	56.6
AF-03	w	20	44	2.93	2.49	37.28	0.03		0.440	0.471	0.092	13.2
AF-04	c	20	71	4.73	3.54	53.05	0.15		0.583	0.657	**0.137**	36.8
AF-05	w	20	59	3.93	2.82	42.34	0.13		0.443	0.492	**0.124**	54.7
AF-06	w	20	67	4.47	3.11	46.68	0.18		0.467	0.542	**0.165**	88.5
AF-07	c	21	63	4.20	3.14	47.16	0.07		0.546	0.582	0.085	84.2
AF-08	c	21	70	4.67	3.47	52.02	0.13		0.559	0.648	**0.161**	100.4
AF-09	c	20	42	2.80	2.35	35.25	0.07	1	0.430	0.459	0.089	18.2
AF-10	w	21	64	4.27	3.24	48.59	0.12		0.543	0.583	0.093	140.7
AF-11	w	21	70	4.67	3.30	49.53	0.19		0.546	0.579	0.08	8.1
AF-12	c	20	55	3.67	2.91	43.59	0.07		0.587	0.565	-0.012	43.9
AF-13	w	20	76	5.07	3.53	52.93	0.14		0.553	0.591	0.089	91.6
AF-14	w	20	54	3.60	2.85	42.73	0.05		0.510	0.504	0.014	13.8
AF-15	w	20	41	2.73	2.54	38.04	0.01		0.573	0.512	-0.095	9.9
AF-16	w	9	47	3.13	2.89	43.39	0.03		0.659	0.562	-0.115	50.7
AF-17	w	20	67	4.47	3.46	51.94	0.08		0.577	0.632	**0.113**	91.6
AF-18	w	20	76	5.07	3.75	56.19	0.10		0.587	0.674	**0.155**	65.7
AF-19	w	10	56	3.73	3.08	46.22	0.14	1	0.653	0.586	-0.062	34.2
AF-20	w	20	66	4.40	3.20	47.94	0.15		0.557	0.577	0.062	109.7
AF-21	w	20	55	3.67	2.84	42.61	0.09		0.493	0.531	0.096	∞
AF-22	c	6	46	3.07	2.89	43.36	0.07		0.644	0.535	-0.115	11.4
AF-23	w	20	63	4.20	2.99	44.81	0.11	1	0.543	0.501	-0.06	39.3
AF-24	w	7	42	2.80	2.64	39.67	0.03		0.676	0.559	-0.136	12.8
AF-25	w	20	72	4.80	3.61	54.21	0.09		0.623	0.658	0.079	50.4
AF-26	w	12	59	3.93	3.13	47.01	0.15		0.633	0.598	-0.015	48.5
AF-27	w	10	56	3.73	3.19	47.90	0.12		0.680	0.606	-0.069	115.9
AF-28	w	14	74	4.93	3.71	55.64	0.11	1	0.710	0.636	-0.079	80.6
AF-29	w	20	77	5.13	3.49	52.29	0.20		0.637	0.602	-0.033	53.7
AS-01	c	50	58	3.87	2.75	41.28	0.07	1	0.432	0.494	**0.135**	193.3
AS-02	c	21	40	2.67	2.17	32.54	0.07		0.371	0.386	0.061	∞
AS-03	c	21	60	4.00	2.98	44.70	0.11		0.454	0.540	**0.183**	104.3
AS-04	c	20	67	4.47	3.41	51.17	0.08		0.540	0.607	**0.135**	326.8
AS-05	c	20	73	4.87	3.53	52.88	0.16	1	0.533	0.620	**0.165**	359.4
AS-06	c	20	66	4.40	3.21	48.09	0.16		0.497	0.572	**0.157**	115.5
AS-07	w	12	53	3.53	2.88	43.18	0.13		0.500	0.485	0.012	89.4
AS-08	c	21	46	3.07	2.34	35.14	0.15	1	0.352	0.391	0.123	92.7
AS-09	c	20	63	4.20	3.11	46.62	0.13		0.477	0.545	**0.15**	135.2

AS-10	c	20	55	3.67	2.91	43.70	0.07		0.483	0.537	**0.126**	102.9
AS-11	w	8	49	3.27	2.94	44.13	0.10		0.617	0.553	-0.05	79.7
AS-12	c	11	67	4.47	3.46	51.93	0.25		0.570	0.633	**0.147**	1308.7
AS-13	w	20	75	5.00	3.52	52.87	0.18		0.497	0.637	**0.244**	77.1
AS-14	c	20	79	5.27	3.56	53.33	0.18		0.557	0.623	**0.132**	68.4
AS-15	c	20	68	4.53	3.36	50.47	0.13		0.540	0.608	**0.138**	22.8
AS-16	c	20	72	4.80	3.46	51.87	0.16		0.567	0.634	**0.131**	44.4
AS-17	w	20	51	3.40	2.48	37.19	0.10		0.407	0.422	0.062	108.1
AS-18	w	20	50	3.33	2.58	38.66	0.07		0.417	0.454	0.108	146.9
AS-19	c	20	47	3.13	2.49	37.32	0.14	2	0.393	0.444	**0.139**	111.6
AS-20	c	20	65	4.33	3.21	48.08	0.11		0.533	0.582	**0.109**	144.5
AS-21	c	20	72	4.80	3.42	51.37	0.21		0.493	0.610	**0.215**	∞
AS-22	c	20	74	4.93	3.56	53.45	0.13		0.577	0.642	**0.128**	268.6
AS-23	c	20	55	3.67	2.55	38.22	0.25		0.370	0.432	**0.169**	402.1
AS-24	c	20	79	5.27	3.51	52.64	0.25		0.577	0.631	**0.111**	289.5
AS-25	w	20	45	3.00	2.37	35.51	0.11		0.360	0.437	**0.2**	42.9
AS-26	w	19	42	2.80	2.25	33.74	0.10		0.386	0.393	0.046	63
AS-27	w	5	32	2.13	2.04	30.56	0.07		0.347	0.307	-0.02	5.3
AS-28	w	11	41	2.73	2.43	36.42	0.04	3	0.473	0.426	-0.061	3.6
AS-29	c	22	35	2.33	2.14	32.16	0.03		0.467	0.417	-0.097	23.7
AU-01	c	20	62	4.13	2.88	43.24	0.16	3	0.450	0.511	**0.144**	∞
AU-02	c	20	56	3.73	2.62	39.23	0.16	2	0.407	0.445	0.112	78.8
AU-03	c	20	57	3.80	2.54	38.16	0.25	1	0.440	0.417	-0.029	15
AU-04	c	20	47	3.13	2.56	38.34	0.07		0.453	0.455	0.028	∞
AU-05	w	18	53	3.53	2.52	37.86	0.16		0.437	0.423	-0.004	122.3
AU-06	c	50	67	4.47	3.06	45.84	0.11		0.551	0.557	0.021	267.4
AU-07	c	20	66	4.40	2.94	44.04	0.24	5	0.467	0.544	**0.167**	192.9
AU-08	w	20	55	3.67	2.77	41.62	0.14	1	0.430	0.523	**0.202**	127
AU-09	c	22	65	4.33	3.30	49.47	0.09		0.545	0.606	**0.123**	95.9
AU-10	c	20	53	3.53	2.41	36.16	0.18		0.377	0.397	0.078	114.3
AU-11	c	20	69	4.60	3.31	49.67	0.14		0.487	0.568	**0.168**	179.2
CA-01	w	15	80	5.33	3.64	54.67	0.21		0.582	0.593	0.052	67.5
CA-02	w	15	84	5.60	3.88	58.21	0.16	1	0.573	0.619	**0.108**	1091.3
CA-03	w	16	86	5.73	3.70	55.46	0.26		0.567	0.591	0.073	∞
CA-04	w	15	75	5.00	3.47	52.00	0.16	1	0.556	0.562	0.046	58.6
CA-05	w	15	79	5.27	3.48	52.16	0.21		0.556	0.551	0.026	195.6
CA-06	w	15	79	5.27	3.58	53.73	0.21		0.578	0.596	0.065	625.6
CA-07	w	20	92	6.13	3.68	55.24	0.28	1	0.547	0.609	**0.128**	243.1
CA-08	w	16	80	5.33	3.56	53.36	0.26	1	0.617	0.621	0.04	∞
CA-09	w	14	93	6.20	4.04	60.53	0.25	1	0.595	0.632	0.095	75.1
CA-10	c	20	65	4.33	3.15	47.32	0.15	2	0.527	0.554	0.074	277.2
CA-11	w	21	55	3.67	2.78	41.72	0.07		0.546	0.511	-0.044	11.1
CA-12	w	20	36	2.40	1.85	27.68	0.16		0.247	0.279	0.141	10.8
CA-13	w	20	93	6.20	3.89	58.28	0.17	1	0.497	0.603	**0.201**	78.5
CA-14	w	20	89	5.93	3.45	51.81	0.30	2	0.497	0.565	**0.146**	∞
CA-15	w	20	67	4.47	3.28	49.19	0.08	2	0.560	0.582	0.063	26.6
CA-16	w	10	65	4.33	3.43	51.46	0.13		0.633	0.599	-0.005	230.1
EU-01	c	21	57	3.80	3.15	47.25	0.04		0.511	0.610	**0.186**	137.5
EU-02	c	21	54	3.60	2.75	41.26	0.14		0.508	0.515	0.037	209.7
EU-03	c	21	51	3.40	2.68	40.23	0.08		0.505	0.516	0.045	∞
EU-04	c	21	60	4.00	2.93	43.99	0.12		0.527	0.531	0.032	508.8
EU-05	c	20	62	4.13	3.10	46.46	0.17	1	0.477	0.576	**0.197**	28.6
EU-06	c	20	65	4.33	3.40	50.94	0.10		0.560	0.642	**0.152**	142.7
EU-07	w	20	56	3.73	2.61	39.14	0.19		0.457	0.467	0.048	63.2
EU-08	c	17	51	3.40	2.82	42.24	0.06		0.478	0.562	**0.178**	40.9
EU-09	c	20	60	4.00	3.23	48.49	0.12		0.553	0.630	**0.147**	∞
EU-10	c	20	53	3.53	2.91	43.66	0.09	1	0.523	0.552	0.077	90.2
EU-11	c	20	49	3.27	2.67	40.10	0.11	1	0.523	0.498	-0.026	25.2
EU-12	c	50	60	4.00	2.90	43.51	0.11		0.496	0.563	**0.129**	109.5
EU-13	c	20	53	3.53	2.84	42.64	0.11		0.533	0.565	0.081	1201.3
EU-14	c	20	56	3.73	2.94	44.17	0.10		0.473	0.549	**0.162**	∞

EU-15	c	20	53	3.53	2.86	42.96	0.11	1	0.497	0.552	0.125	58.5
EU-16	c	15	51	3.40	2.65	39.75	0.13		0.556	0.484	-0.113	194.5
EU-17	c	21	77	5.13	3.40	50.98	0.27		0.606	0.629	0.06	28.6
EU-18	c	20	56	3.73	2.83	42.49	0.19	2	0.487	0.542	**0.128**	33.9
EU-19	c	20	44	2.93	2.57	38.49	0.06		0.493	0.529	0.094	22.4
EU-20	c	20	41	2.73	2.24	33.61	0.11		0.460	0.427	-0.052	30.9
EU-21	c	18	47	3.13	2.62	39.26	0.06	1	0.526	0.480	-0.068	32.4
EU-22	c	20	47	3.13	2.51	37.68	0.07		0.407	0.464	**0.148**	21.9
EU-23	c	20	52	3.47	2.77	41.56	0.13		0.480	0.544	**0.143**	119.3
EU-24	w	12	46	3.07	2.59	38.87	0.05		0.483	0.463	-0.001	∞
EU-25	c	20	88	5.87	3.73	55.92	0.23	2	0.563	0.647	**0.154**	507.4
EU-26	c	20	69	4.60	3.38	50.67	0.13		0.563	0.609	**0.101**	91.5
EU-27	w	14	78	5.20	3.64	54.66	0.22		0.576	0.604	0.083	60.8
EU-28	w	9	55	3.67	3.25	48.72	0.06	2	0.689	0.599	-0.093	23.3
EU-29	c	20	55	3.67	2.84	42.54	0.13		0.503	0.550	0.11	∞
EU-30	c	20	58	3.87	2.94	44.14	0.17		0.580	0.576	0.019	675.9
EU-31	c	20	60	4.00	3.08	46.25	0.10	1	0.593	0.590	0.019	32.3
EU-32	c	25	31	2.07	1.96	29.39	0.00		0.325	0.341	0.067	12.1
EU-33	w	15	44	2.93	2.53	38.00	0.07		0.511	0.477	-0.037	492.5
EU-34	c	18	64	4.27	3.24	48.62	0.10		0.604	0.590	0.005	∞
EU-35	c	18	57	3.80	3.07	46.08	0.14	1	0.581	0.576	0.019	64
EU-36	c	14	46	3.07	2.57	38.62	0.10		0.490	0.465	-0.018	79.7
EU-37	w	6	27	1.80	1.76	26.47	0.00		0.411	0.281	-0.386	∞
EU-38	w	20	44	2.93	2.60	38.98	0.00	1	0.443	0.491	0.122	2
NA-01	c	20	54	3.60	2.66	39.97	0.21	1	0.450	0.508	**0.14**	359.8
NA-02	w	14	60	4.00	3.16	47.47	0.12		0.581	0.564	0.007	44.9
NA-03	c	20	67	4.47	3.21	48.14	0.12		0.520	0.566	0.107	39.9
NA-04	c	20	61	4.07	3.09	46.41	0.13	1	0.520	0.564	0.104	653.4
NA-05	c	20	61	4.07	2.91	43.62	0.24		0.547	0.554	0.039	170.7
NA-06	c	20	59	3.93	2.83	42.48	0.23		0.487	0.532	0.11	698.7
NA-07	w	20	85	5.67	3.86	57.91	0.14		0.533	0.665	**0.222**	292
NA-08	c	20	59	3.93	2.93	43.90	0.15		0.513	0.553	0.097	280.4
NA-09	c	20	64	4.27	3.04	45.53	0.15	1	0.520	0.557	0.092	1695.9
NA-10	c	20	55	3.67	2.78	41.72	0.14		0.560	0.538	-0.016	49.7
NA-11	w	21	75	5.00	3.44	51.65	0.15		0.584	0.598	0.047	256.5
NA-12	c	20	55	3.67	2.99	44.80	0.10		0.583	0.558	-0.019	69.1
NA-13	c	20	57	3.80	2.82	42.35	0.17		0.503	0.527	0.07	748.4
NA-14	w	20	76	5.07	3.35	50.32	0.15		0.583	0.583	0.025	94.2
NA-15	c	20	51	3.40	2.58	38.65	0.10		0.430	0.475	0.12	54.1
SA-01	w	20	107	7.13	4.08	61.21	0.33	5	0.540	0.666	**0.213**	712.4
SA-02	c	20	106	7.07	4.01	60.14	0.37	4	0.533	0.641	**0.192**	153.6
SA-03	w	20	113	7.53	4.51	67.61	0.16	5	0.717	0.709	0.014	483.1
SA-04	c	20	87	5.80	3.77	56.50	0.27	3	0.570	0.649	**0.146**	63.3
SA-05	c	20	71	4.73	3.42	51.28	0.14		0.533	0.603	**0.14**	452.4
SA-06	c	10	50	3.33	2.62	39.27	0.29	1	0.333	0.434	**0.281**	40.9
SA-07	w	10	32	2.13	1.88	28.14	0.11		0.340	0.281	-0.158	20.6
SA-08	w	5	52	3.47	3.06	45.87	0.40		0.640	0.501	-0.171	∞
SA-09	w	20	107	7.13	4.11	61.72	0.34	2	0.497	0.644	**0.253**	262.6
SA-10	w	20	75	5.00	3.29	49.33	0.20	1	0.543	0.563	0.06	18.8
SA-11	w	21	72	4.80	3.17	47.50	0.21	3	0.565	0.565	0.025	20.1
SA-12	c	20	75	5.00	3.13	46.91	0.27	3	0.493	0.527	0.09	64.7

Table S4. Countries of origin of investigated *Hermetia illucens* populations.

Countries are listed alphabetically within subcontinents. Numbers of sampled countries per subcontinent are indicated, as well as sampled states, provinces or territories within countries (where informative). Numbers in brackets specify numbers of samples from any given country, highlighted in italics if including wild populations. For more details see Table S1, Additional file 1 (in several cases more detailed information could not be disclosed for captive populations because several commercial providers wished to stay anonymous).

**Subcontinent**	**Countries**	**No. countries**
Africa	Cote d'Ivoire [*1*], Benin [*2*], Burkina-Faso [*1*], Ghana [*5*], Guinea-Bissau [*1*], Kenya [*7*], Malawi [*4*], South Africa [*2*], Tanzania [*1*], Tunisia [2], Uganda [*1*], Zambia [*1*], Zimbabwe [*1*]	13
Asia (including Sundaland)	Cambodia [*1*], China (5 provinces: Guangdong, Henan, Hubei, Shaanxi, Shanghai [5]), Indonesia [*2*], Japan [1], Malaysia [*4*], Philippines [1], Singapore [3], South Korea [1], Thailand [*5*], Taiwan [5], Vietnam [1]	11
Australia (including Polynesia)	Australia (5 territories: ACT, NSW, QLD, WA, VIC [*8*]), New Zealand [*2*], Hawaii (USA, [1])	2 (3)
Central America (including the Caribbean)	Costa Rica [*1*], Guatemala [*1*], Honduras [1], Mexico (4 states: CS, NL, OA, VE [*9*]), Panama [*3*], Puerto Rico [*1*]	6
Europe	Austria [1], Belgium [3], Croatia [*1*], Denmark [1], France [*4*], Germany [1], Greece [1], Italy [*7*], Ireland [1], Netherlands [2], Poland [1], Portugal [1], Russia [1], Sweden [1], Spain [2], Switzerland [*6*], United Kingdom [4]	17
North America	Canada [1], USA (13 states: CA, FL, IL, KY, LA, MO, NJ, OH, OK, OR, PA, TX, WA [*14*], plus HI, see above)	2
South America	Bolivia [*2*], Brazil (4 states: AM, GO, PA, SP [*5*]), Colombia [*2*], Ecuador [1], Paraguay [*1*], Peru [*1*]	6

Table S5. Population genetic characteristics according to subcontinental origin and provenance status (wild vs. captive).

Pops: number of populations per group;
Ind: number of individuals across populations per group;
No*A*: total number of different alleles detected across populations;
A_P: private alleles uniquely present within populations of a given group (referring to the overall sample: alleles uniquely detected in a single population);
N_A/Pop: mean number of alleles per population (not rarefied) and respective standard deviations;
sum A_R: sum of allelic richness across loci, rarefied to 5 diploid individuals per population, and respective standard deviations across populations;
A_R: mean allelic richness per locus, rarefied to 5 diploid individuals per population, and respective standard deviations across populations;
H_O: mean observed heterozygosity and respective standard deviations across populations;
H_E: mean expected heterozygosity and respective standard deviations across populations;
Ne: group averages of inferred effective population size based on the linkage disequilibrium method, treating infinite population sizes (Table S3) conservatively as 1,700 individuals (considering the highest precisely calculated value reached 1,696);
F_{ST}: overall genetic differentiation across populations within groups (significance indicated in bold);
NA: not applicable.

Subcontinent	Provenance	# Pops	# Ind	No*A*	A_P	N_A/Pop	sd	sum A_R	sd	A_R	sd	H_O	sd	H_E	sd	N_e	F_{ST}
ALL	all	150	2862	368	82	61.83	15.48	45.58	7.40	3.04	0.49	0.51	0.09	0.54	0.08		**0.239**
	wild	66	1096	315	38	64.23	18.95	46.84	8.82	3.12	0.59	0.53	0.11	0.54	0.10	296.3	**0.234**
	captive	84	1766	295	44	60.02	12.02	44.64	6.00	2.98	0.40	0.50	0.09	0.54	0.07	353.3	**0.216**
Africa	all	29	542	186	6	60.31	11.03	46.43	5.65	3.10	0.38	0.55	0.09	0.57	0.06		**0.224**
	wild	22	384	173	5	61.23	11.08	46.94	5.43	3.13	0.36	0.56	0.11	0.57	0.05	133.2	**0.215**
	captive	7	158	105	1	57.43	11.21	44.83	6.45	2.99	0.43	0.54	0.12	0.57	0.07	63.2	**0.099**
Asia	all	29	561	177	8	57.90	13.62	43.77	7.67	2.92	0.51	0.47	0.12	0.52	0.10		**0.207**
	wild	9	135	119	3	48.67	11.80	39.14	6.68	2.61	0.45	0.44	0.18	0.46	0.09	68.4	**0.188**
	captive	20	426	160	5	62.05	12.50	45.85	7.29	3.06	0.49	0.48	0.12	0.55	0.09	375.7	**0.191**
Australia	all	11	250	150	12	59.09	7.09	42.15	4.71	2.81	0.31	0.47	0.17	0.49	0.07		**0.183**
	wild	2	38	77	1	54.00	1.41	39.74	2.66	2.65	0.18	0.43	0.15	0.47	0.07	124.7	**0.235**
	captive	9	212	147	11	60.22	7.40	42.68	5.01	2.85	0.33	0.48	0.18	0.50	0.07	482.6	**0.180**
Central America	all	16	272	212	12	76.13	15.45	51.43	7.78	3.43	0.52	0.53	0.16	0.57	0.08		**0.152**
	wild	15	252	206	10	76.87	15.70	51.70	7.97	3.45	0.53	0.53	0.16	0.57	0.09	520.9	**0.153**
	captive	1	20	65	2	65.00	NA	47.32	NA	3.15	NA	0.53	0.28	0.55	NA	36.3	NA
Europe	all	38	736	184	14	55.26	12.02	43.09	6.17	2.87	0.41	0.51	0.11	0.54	0.08		**0.188**
	wild	7	96	123	3	50.00	15.61	40.69	8.92	2.71	0.59	0.50	0.20	0.48	0.11	577.4	**0.232**
	captive	31	640	147	11	56.41	11.06	43.61	5.45	2.91	0.36	0.51	0.13	0.55	0.07	419.4	**0.139**
North America	all	15	295	149	3	62.60	9.45	45.66	4.96	3.04	0.33	0.53	0.15	0.56	0.04		**0.118**
	wild	4	75	127	0	74.00	10.36	51.84	4.41	3.46	0.29	0.57	0.13	0.60	0.04	171.9	**0.103**
	captive	11	220	103	3	58.45	4.68	43.41	2.78	2.89	0.19	0.51	0.17	0.54	0.03	438.2	**0.068**
South America	all	12	206	239	27	78.92	26.01	51.29	10.98	3.42	0.73	0.54	0.15	0.57	0.12		**0.135**
	wild	7	116	190	16	80.50	33.76	52.01	14.49	3.47	0.97	0.56	0.14	0.56	0.16	459.7	**0.131**
	captive	5	90	177	11	77.33	18.51	50.57	7.35	3.37	0.49	0.52	0.17	0.57	0.08	155.0	**0.139**

Table S6. Diversity and pairwise differentiation of globally inferred *Hermetia illucens* genetic clusters.

A) Number of individuals assigned to each of the inferred 16 clusters (see Figure 1 and Table 2), total as well as locus-specific allele numbers within clusters (see also Table 1), and sum of allelic richness A_R across loci (rarefied to 20 diploid individuals). Cluster-specific private alleles (independent of sampling populations) are indicated in italics in parentheses for individual loci. B) Pairwise F_{ST} between inferred clusters (all comparisons $p < 0.001$); see also Figure S5 for pairwise genetic differentiation among populations sampled.

A)

	**Inferred cluster**															
	**1**	**2**	**3**	**4**	**5**	**6**	**7**	**8**	**9**	**10**	**11**	**12**	**13**	**14**	**15**	**16**
**# Individuals**	504	290	203	75	234	283	79	109	65	63	167	159	135	115	187	194
**# Alleles**	145	136	108	90	140	143	84	103	96	92	119	217	133	142	187	167
**Hi_1-1**	12	16	11	8	16	18	6	9	11	8	13 (*1*)	22 (*1*)	16	14	20	18
**Hi_1-2**	13 (*4*)	9	9	7	11	16	9 (*2*)	14 (*2*)	8 (*1*)	8	12	19 (*2*)	13 (*1*)	12 (*1*)	17	15
**Hi_1-3**	11 (*1*)	8	5	5	8	6 (*1*)	2	3	4	4	5	17 (*7*)	10 (*1*)	6 (*1*)	10 (*2*)	6
**Hi_1-4**	8	7	10 (*2*)	8	10	8 (*1*)	6	8 (*1*)	9 (*3*)	5	7	15 (*3*)	10 (*1*)	6 (*1*)	10	11 (*1*)
**Hi_1-5**	7	7	5	4	8	8	4	5	3	4 (*1*)	6	10 (*1*)	9 (*1*)	6	9	9
**Hi_2-1**	5	6	3	4	6	6 (*1*)	4	6	5 (*1*)	5	6	9	6	5	8	9
**Hi_2-2**	13 (*2*)	11	8	6	12	13 (*1*)	7 (*1*)	10	7	9	7	18 (*4*)	8	10	10	10
**Hi_2-3**	10	8	7	7	10 (*1*)	8	5	6	5	6	7	11 (*2*)	8 (*1*)	10 (*1*)	10	12
**Hi_2-4**	10	7	5	8	11	9	3	5	5	7	9	8 (*1*)	10	17 (*1*)	26 (*3*)	15
**Hi_2-5**	4	5	4	3	4	4	1	2	3	2	5	6	4 (*1*)	4	5	6
**Hi_3-1**	12	15	8 (*1*)	5	12 (*1*)	12 (*1*)	7	5	9	1	6	14 (*2*)	4	7 (*2*)	6	9 (*1*)
**Hi_3-2**	9	11	8 (*1*)	5	7	9 (*1*)	5	6	6	7	12	15 (*1*)	11	13	18 (*4*)	15 (*1*)
**Hi_3-3**	12 (*1*)	10	9	6	9	8	7	5	7	6	6	15 (*1*)	8	14	19 (*2*)	12
**Hi_3-4**	12	8	9	8	9	12 (*1*)	13	11	9 (*1*)	13	11	25 (*4*)	9 (*1*)	12	11	13
**Hi_3-5**	7	8	7	6	7	6	5	8	5	7	7	13	7	6 (*2*)	8	7
**Sum A_R**	64.82	71.47	65.63	66.17	84.09	75.13	58.63	75.35	71.22	72.49	81.73	120.70	89.08	93.78	111.89	103.02

B)

	1	2	3	4	5	6	7	8	9	10	11	12	13	14	15	16
**1**																
**2**	0.047															
**3**	0.066	0.101														
**4**	0.172	0.135	0.199													
**5**	0.110	0.093	0.131	0.096												
**6**	0.250	0.238	0.273	0.289	0.116											
**7**	0.328	0.298	0.359	0.377	0.221	0.167										
**8**	0.255	0.245	0.268	0.288	0.130	0.089	0.146									
**9**	0.237	0.220	0.265	0.286	0.156	0.122	0.209	0.133								
**10**	0.286	0.254	0.308	0.293	0.166	0.204	0.246	0.126	0.204							
**11**	0.219	0.192	0.240	0.258	0.150	0.164	0.226	0.142	0.178	0.146						
**12**	0.209	0.200	0.220	0.201	0.131	0.173	0.245	0.128	0.149	0.138	0.134					
**13**	0.269	0.247	0.280	0.260	0.193	0.237	0.310	0.193	0.212	0.221	0.195	0.100				
**14**	0.175	0.178	0.193	0.171	0.105	0.191	0.289	0.176	0.178	0.196	0.157	0.083	0.150			
**15**	0.168	0.157	0.219	0.196	0.109	0.166	0.256	0.179	0.159	0.187	0.098	0.125	0.200	0.104		
**16**	0.084	0.075	0.125	0.168	0.072	0.156	0.240	0.171	0.140	0.182	0.092	0.128	0.187	0.100	0.054	

Table S7. AMOVA nesting provenance (wild vs. captive) within subcontinents for worldwide *Hermetia illucens* populations.

Genetic variance explained according to provenance (wild or captive) nested within subcontinents. Significant variance components ($p < 0.01$) are highlighted in bold.

More details on patterns according to analyses that specifically test for subcontinental origin within different provenances, as well as provenances within different subcontinents, are given in Table 3 and the main text. There, for instance, the substantial structure revealed particularly for Europe reflects independent introductions of genetically distinct captive populations to Europe (notably from North America and Asia) that are contrasted by highly diverse wild populations found in different European regions (see Table 3b).

Source of variance	Proportion of variance (%)
Between subcontinents	**2.4**
Between provenances within subcontinents	**8.9**
Between populations within provenances	**14.3**
Between individuals within populations	**6.3**
Within individuals	**68.1**

Table S8. Model-based estimates of contrasts and significance levels for population-specific allelic richness.

Three separate linear mixed models on allelic richness were performed, including locus as random effect and the following fixed effects: A) provenance status (wild vs. captive), B) subcontinent of origin, and C) provenance nested within subcontinent (see Table panels). Model assumptions were fulfilled, so model-based estimates are reported on the response scale for post-hoc Tukey contrasts in a), b) and c).

Rarefaction was applied throughout based on the lowest number of individuals across populations (5 diploid specimen). For the most complex model (c), besides all pairwise contrasts for exclusively captive and exclusively wild-sourced populations within subcontinents, pairwise contrasts of wild versus captive populations are only reported for inter-continental comparisons (shaded in grey). Raw data are in Table S3. *** $p < 0.0001$; ** $p < 0.01$; * $p < 0.05$; . $p < 0.1$.

Overall model statistics:

Mixed model	Fixed effect	DFs	*F*
A (cf. panel a below)	Population provenance	1, 2235	16.436***
B (cf. panel b below)	Subcontinent	6, 2235	22.032***
C (cf. panel c below)	Provenance nested within subcontinent	13, 2235	14.612***

Pairwise post-hoc contrasts:

a)	Fixed effect: population provenance	Estimate
	wild vs. captive (global)	0.152***

b)	Fixed effect: subcontinents			Estimate
	Asia	vs.	Africa	-0.178*
	Australia	vs.	Africa	-0.286**
	Central America	vs.	Africa	0.333***
	Europe	vs.	Africa	-0.223***
	North America	vs.	Africa	-0.052
	South America	vs.	Africa	0.324***
	Australia	vs.	Asia	-0.108
	Central America	vs.	Asia	0.511***
	Europe	vs.	Asia	-0.045
	North America	vs.	Asia	0.126
	South America	vs.	Asia	0.502***
	Central America	vs.	Australia	0.619***
	Europe	vs.	Australia	0.063
	North America	vs.	Australia	0.234
	South America	vs.	Australia	0.610***
	Europe	vs.	Central America	-0.556***
	North America	vs.	Central America	-0.384***
	South America	vs.	Central America	-0.009
	North America	vs.	Europe	0.172
	South America	vs.	Europe	0.547***
	South America	vs.	North America	0.375***

c)	Fixed effect: population provenance nested within subcontinents			Estimate
	captive Asia	vs.	captive Africa	0.068
	captive Australia	vs.	captive Africa	-0.143
	captive Central America	vs.	captive Africa	0.166
	captive Europe	vs.	captive Africa	-0.099
	captive North America	vs.	captive Africa	-0.094
	captive South America	vs.	captive Africa	0.382.
	captive Australia	vs.	captive Asia	-0.211
	captive Central America	vs.	captive Asia	0.098
	captive Europe	vs.	captive Asia	-0.166
	captive North America	vs.	captive Asia	-0.162
	captive South America	vs.	captive Asia	0.315.
	captive Central America	vs.	captive Australia	0.309
	captive Europe	vs.	captive Australia	0.045
	captive North America	vs.	captive Australia	0.049
	captive South America	vs.	captive Australia	0.526***
	captive Europe	vs.	captive Central America	-0.264
	captive North America	vs.	captive Central America	-0.260
	captive South America	vs.	captive Central America	0.217
	captive North America	vs.	captive Europe	0.004
	captive South America	vs.	captive Europe	0.481***
	captive South America	vs.	captive North America	0.477**
	wild Africa	vs.	captive Africa	0.141
	wild Asia	vs.	captive Asia	-0.447***
	wild Australia	vs.	captive Australia	-0.196
	wild Central America	vs.	captive Central America	0.292
	wild Europe	vs.	captive Europe	-0.177
	wild North America	vs.	captive North America	0.562***
	wild South America	vs.	captive South America	0.096
	wild Asia	vs.	wild Africa	-0.520***
	wild Australia	vs.	wild Africa	-0.480
	wild Central America	vs.	wild Africa	0.317***
	wild Europe	vs.	wild Africa	-0.417***
	wild North America	vs.	wild Africa	0.326
	wild South America	vs.	wild Africa	0.337*
	wild Australia	vs.	wild Asia	0.040
	wild Central America	vs.	wild Asia	0.837***
	wild Europe	vs.	wild Asia	0.104
	wild North America	vs.	wild Asia	0.847***
	wild South America	vs.	wild Asia	0.858***
	wild Central America	vs.	wild Australia	0.797***
	wild Europe	vs.	wild Australia	0.063
	wild North America	vs.	wild Australia	0.806**
	wild South America	vs.	wild Australia	0.818***
	wild Europe vs. wild	vs.	wild Central America	-0.734***
	wild North America	vs.	wild Central America	0.009
	wild South America	vs.	wild Central America	0.020
	wild North America	vs.	wild Europe	0.743***
	wild South America	vs.	wild Europe	0.754***
	wild South America	vs.	wild North America	0.011

Table S9. Genetic isolation by distance for selected hierarchical groupings.

Mantel-test-inferred statistics based on Pearson's product-moment correlation. Pairwise genetic (F_{ST} / (1 - F_{ST})) and geographic (log kilometres) distances were analysed for hierarchical groupings of interest according to subcontinental origin and/or provenance status. Significance, based on 9999 permutations, is indicated in bold.

**Analysed group**	**# Populations**	**Isolation by distance**
Global sample, wild and captive	150	***r* = 0.214**
Global sample, wild	66	***r* = 0.317**
Global sample, captive	84	***r* = 0.339**
All Americas, wild and captive	43	***r* = 0.350**
All Americas, wild	26	***r* = 0.349**
South America, wild and captive	12	*r* = 0.219
South America, wild	7	***r* = 0.149**
Central America, wild	16	***r* = 0.466**
Central America, wild and captive	15	***r* = 0.476**
North America, wild and captive	15	***r* = 0.239**
North America, wild	4	*r* = 0.032
Africa, wild and captive	29	***r* = 0.411**
Africa, wild	22	***r* = 0.497**
Asia, wild and captive	29	***r* = 0.21**
Asia, wild	9	***r* = 0.403**
Australia, wild and captive	11	***r* = 0.416**
Australia, wild	2	not applicable
Asia and Australia, wild and captive	40	***r* = 0.236**
Asia wild and Australia wild and captive	12	***r* = 0.413**
Europe, wild and captive	38	*r* = 0.019
Europe, wild	7	***r* = 0.389**

Table S10. Specifications on the global distribution of *Hermetia illucens* genetic clusters presented in Figure 4.

Summary of the major geographic regions of interest. Listed are sampled countries (alpha-3 code, ISO 3166-1) and states/territories (where relevant), provenances (wild vs. captive), and numbers of individuals and populations as represented by the pie charts depicted in Figure 4. Details about inferred underlying demography for each major geographic region as identified by roman numbers in Figure 1 are described in the sub-table below. More information about several commercial captive samples (beyond continental scales) could not be disclosed. For additional details see also Tables S1, S4, S6 and S11-13, as well as Figures S4-8.

No.	Subcontinent	Major region	Countries (*state/territory*)	Provenance	# individuals (# populations)
I	Africa	whole	GHA, KEN, TUN, ZAF	captive	158 (*7*)
II	Africa	west	CIV, BEN, BFA, GHA, GNB	wild	156 (*9*)
III	Africa	central-east	KEN, UGA	wild	87 (*5*)
IV	Africa	south-east	MWI, TZA, ZAF, ZMB, ZWE	wild	141 (*8*)
V	Asia	whole	CHN, IDN, JPN, KOR, MYS, PHL, SGP, TWN, VNM	captive	426 (*20*)
VI	Asia	south-east continental	KHM, THA	wild	95 (*6*)
VII	Asia	south-east insular	IDN, MYS	wild	40 (*3*)
VIII	Australia	west	AUS (*WA*)	wild & captive	78 (*4*)
IX	Australia	east	AUS (*ACT, NSW, QLD, VIC*)	captive	110 (*4*)
X	Australia	Polynesia	NZL	wild & captive	40 (*2*)
XI	Australia	Polynesia	USA (*HI*)	captive	22 (*1*)
XII	Central America	north	MEX (*NL*)	wild	20 (*1*)
XIII	Central America	central	CRI, GTM, HND, MEX (*CS, OA, VE*)	wild & captive	175 (*11*)
XIV	Central America	south	PAN	wild	57 (*3*)
XV	Central America	Caribbean	PRI	wild	20 (*1*)
XVI	Europe	whole	AUT, BEL, CHE, DNK, FRA, DEU, ESP, GBR, GRC, ITA, IRL, NLD, POL, PRT, RUS, SWE	captive	640 (*31*)
XVII	Europe	west	CHE (*BS, BL*), FRA (*ARA*), ITA (*EMI, LAZ*)	wild	62 (*5*)
XVIII	Europe	south	ITA (*PUG*)	wild	14 (*1*)
XIX	Europe	south-east	HRV	wild	20 (*1*)
XX	North America	whole	CAN, USA	captive	220 (*11*)
XXI	North America	west	USA (*CA*)	wild	34 (*2*)
XXII	North America	south-east	USA (*FL, LA*)	wild	41 (*2*)
XXIV	South America	central-east	BOL, BRA, PRY	wild & captive	161 (*8*)
XXIII	South America	north-west	COL, ECU, PER	wild & captive	45 (*4*)

I:	Dominated by introduced domesticated strains from North America (XX) and Europe (XVI), yet also comprising captive stocks derived from naturalised wild populations of cluster 16 and introgressants of cluster 11, respectively (see III); pure locally naturalised clusters 11 and 13 were virtually absent across African captive populations.
II:	Mostly cluster 13, going back to a historic introduction from a single source in central-east South America (XXIV); genetically homogenous across entire western Africa, except for few indications of introgression from recently introduced domesticated strains.
III:	Admixture between south-east African cluster 11 (IV) and domesticated captive populations plus a separate single founder population of cluster 16, either from North America directly (XXI or XXII) or via Mediterranean Europe (XVIII or XIX).
IV:	Pure cluster 11, going back to a secondary admixture event between previously established western African cluster 13 (II) and early Polynesian colonisers (primarily admixed group XI); genetically homogenous across entire south-eastern Africa.
V:	Asian captive populations comprise a large fraction of regionally naturalised origins (VI), but mostly more recently derived hybrids and introgressants (cluster 5 and 4, respectively) due to rare yet highly influential introductions of domesticated strains from North America (XX).
VI:	Pure cluster 6, directly deriving from Polynesian primary founders of the Australasian range expansion (XI); genetically homogenous naturalised lineage across eastern and south-eastern continental and insular Asia.
VII:	Largely cluster 6 (VI), yet Sunda Islands are characterised by widespread introgression into locally naturalised populations from commonly farmed domesticated strains of North American origin (XX).
VIII:	Pure cluster 7, derived from admixture between Asian naturalised cluster 6 (VII) and eastern Australian naturalised cluster 8 (IX), thus representing the youngest distinct group of the Australasian serial colonisation; genetically homogenous across western Australia.
IX:	Pure cluster 8, diverged from cluster 9 (X) that previously colonised nearby Pacific Islands; genetically homogenous across eastern Australia.
X:	Pure cluster 9, diverged from Asian naturalised cluster 6 (VI) upon colonising Australasia from northern Polynesian areas (XI) in a counter-clockwise manner; Pacific Islands thus feature differentiated start and endpoints of this range expansion.
XI:	Admixed cluster memberships originating from independent introductions from western North America (XXI) and north-western South America (XXIII); constituted the primarily admixed Polynesian bridgehead population that mediated serial colonisations across entire Australasia and secondary admixture events worldwide.
XII:	Northern Mexico appears to be the region where North American cluster 16 displaces Central American cluster 15 (see also XIII).
XIII:	Cluster 15 dominates Central America where it derived from cluster 14 (XIV) during northward range expansions (north of the Panamanian isthmus); found across provenances.
XIV:	Cluster 14 (XXIII) that further expanded its range into southern Central America without notable genetic changes (Panama only).
XV:	Pure cluster 9; inferred admixed ancestry primarily involving south-eastern North American and central-eastern South American lineages, plus limited gene flow from Central America.
XVI:	North American introductions of domesticated strains (comparatively diverse cluster 2 around 2005 and more recent ones, notably cluster 1) and a regional breeding program (cluster 3), plus recent introductions of captive stocks from Africa (indistinguishable) and Asian hybrids (cluster 5); absence of pure or introgressed European naturalised clusters in captivity.
XVII:	Unique cluster 10, going back to an admixture event between independent south-eastern African (IV) and eastern Australian (IX) introductions; genetically homogenous across western and central Europe.
XVIII:	Largely cluster 15, indicating a unique introduction from central regions of Central America, plus gene flow from central Europe (XVII), as well as south-eastern Europe (XIX) and/or central-eastern Africa (III).
XIX:	Clusters 14 and 16 (lacking individual admixture) point to a unique introduction from southern Central America (XIV) or north-western South America (XXIII) to south-eastern Europe, plus an independent introduction of North American wild populations (XXI or XXII) and/or gene flow with central-eastern African populations (III).
XX:	Exclusively captive populations largely composed of cluster 1 going back to wild founders from Georgia/USA; origin of most captive populations worldwide, showing striking signatures of domestication; neither pure wild-type genetics nor notable gene flow from these were detected in captivity.
XXI:	Pure cluster 16, at the terminus of the northwards range expansion across the Americas.
XXII:	Largely cluster 16 (see XXI), yet with characteristic signatures of introgression from other American regions owing to gene flow via the Caribbean.
XXIII:	Cluster 14, derived from central-eastern South American cluster 12 (XXIV) upon range expansions beyond the Andes into north-western South America, where it is genetically homogenous and found across provenances.
XXIV:	Ancestral indigenous cluster 12 comprising the genetic hot spots of the species; widespread and genetically homogenous throughout central-eastern South America and found across provenances.

Table S11. Details on ABC analyses.

Table showing analysis ID and description, the number of samples used for each analysis, the number of tested models and corresponding simulations, and the range of uniform prior distributions for each of the model parameters. If more than a single population was available from a given region/genetic group of interest, populations included in the analyses were selected based on the following criteria: (1) membership of all individuals to a genetic cluster of interest (Fig. 1, Table 2), (2) preference of wild populations from a given cluster/region, (3) preference of populations with comparatively high allelic richness (Table S3) that group basal in a given cluster within the same characteristic clade (Fig. 3, Figure S5), and (4) whenever available, knowledge on complementary mitochondrial data based on a separate study (Ståhls et al. 2020). Individual analyses regarding non-native colonisation routes were largely built on available documentation records (see Introduction and references cited in this context). Individual analyses are presented in a sequential order as increasing information content was incorporated to build models for subsequent analyses focussing on supposedly successive colonisation events across non-native ranges (if indicated). See Tables S12 and S13 for further details.

Individual analyses addressed key questions and focal genetic groups including sample sets as follows:

Analysis A): Indigenous American range expansions – Which is the ancestral / most recently derived group? N1: North American cluster 16; N2: Central American cluster 15; N3: north-west South American / southern Central American cluster 14; N4: South American cluster 12.

Analysis B): Origin of North American domesticated populations – Is there a specific American source? N1: exclusively captive cluster 1 (North American); N2: south-eastern North American (cluster 16); N3: western North American (cluster 16); N4: Central American cluster 15; N5: South American cluster 12.

Analysis C): Origin of distinct Caribbean populations – Is there a single American source or are these the result of admixture? N1: South American cluster 12; N2: Caribbean cluster 9; N3: south-eastern North American wild (cluster 16); N4: Central American cluster 15; N5: north-west South American / southern Central American cluster 14.

Analysis D): Colonisation of Australasia – Which origin and which is the most recently derived group? N1: Hawaii (admixed); N2: Asian cluster 6; N3: Polynesian cluster 9; N4: eastern Australian cluster 8; N5: western Australian cluster 7.

Analysis E): Origin of Polynesian populations – Is there a single American source or are these the result of admixture? N1: South American cluster 12; N2: Hawaii (admixed); N3: Polynesian cluster 9; N4: North American cluster 16; N5: Central American cluster 15; N6: north-west South American / southern Central American cluster 14.

Analysis F): Origin of southeast African cluster 11 – Is there a single source or are these the result of admixture? N1: South American cluster 12; N2: North American cluster 16; N3: Hawaii (admixed); N4: west African cluster 13; N5: south-east African cluster 11.

Analysis G): Origin of western European cluster 10 – Is there a single source or are these the result of admixture? N1: west African cluster 13; N2: Mediterranean European (admixed); N3: Hawaii (admixed); N4: eastern Australian cluster 8; N5: south-east African cluster 11; N6: western European cluster 10.

Analysis ID	Description	# of samples	# of models	# simulations	Range of uniform prior distributions												
					**N1**	**N2**	**N3**	**N4**	**N5**	**N6**	**t1**	**t2**	**t3**	**t4**	**t5**	**r1**	r2
A	Americas	4	3	3 000 000	10;10000	10;10000	10;10000	10;10000			10;5000	10;5000	10;5000				
B	North American captive	4	4	4 000 000	10;10000	10;10000	10;10000	10;10000	10;10000		10;1000	10;5000	10;5000			0.001;0.999	
C	Caribbean	5	8	8 000 000	10;10000	10;10000	10;10000	10;10000	10;10000		10;10000	10;10000	10;10000	10;10000		0.001;0.999	
D	Australasia	5	16	16 000 000	10;10000	10;10000	10;10000	10;10000	10;10000		10;1000	10;3000	10;5000	10;10000		0.001;0.999	
E	Polynesia	6	8	8 000 000	10;10000	10;10000	10;10000	10;10000	10;10000	10;10000	10;1000	10;5000	10;10000	10;10000	10;10000	0.001;0.999	
F	Southeast Africa	5	10	10 000 000	10;10000	10;10000	10;10000	10;10000	10;10000		10;10000	10;10000	10;10000	10;10000		0.001;0.999	
G	Western Europe	6	10	10 000 000	10;10000	10;10000	10;10000	10;10000	10;10000	10;10000	10;1000	10;3000	10;5000	10;10000	10;10000	0.001;0.999	0.001;0.999

Table S12. Posterior probabilities of demographic models inferred from ABC analyses.

Posterior probabilities and 95% confidence intervals for demographic models compared in seven separate analyses (sub-tables A-G). Significantly best-fitting models are highlighted with asterisks.

Analysis	Model	Posterior probability	CI-	CI+
A) Americas	1*	0.8501	0.0541	0.054
	2	0.1467	0.0535	0.0534
	3	0.0033	0.0033	0.0086
B) North American captive	1*	0.4726	0.008	0.0079
	2	0.1062	0.0046	0.0046
	3	0.1642	0.0059	0.0059
	4	0.2571	0.0071	0.007
C) Caribbean	1*	0.4451	0.088	0.0881
	2	0.0231	0.0231	0.0901
	3	0.186	0.0709	0.0708
	4	0.1331	0.0756	0.0756
	5	0.0475	0.0475	0.0846
	6	0.0712	0.0712	0.1441
	7	0.067	0.067	0.0805
	8	0.027	0.027	0.0897
D) Australasia	1	0.0001	0.0001	0.0389
	2	0	0	0.0389
	3	0	0	0.0389
	4	0	0	0.0389
	5	0	0	0.0389
	6	0	0	0.0389
	7	0	0	0.0389
	8	0.0001	0.0001	0.0388
	9	0.001	0.001	0.0386
	10*	0.7665	0.1288	0.1288
	11	0	0	0.0389
	12	0	0	0.0389
	13	0	0	0.0389
	14	0.0001	0.0001	0.0388
	15	0.2322	0.1287	0.1286
	16	0	0	0.0389

E) Polynesia	1	0.241	0.0145	0.0146
	2	0.0173	0.0057	0.0057
	3	0.0526	0.0057	0.0057
	4	0.0146	0.006	0.006
	5*	0.495	0.0168	0.0168
	6	0.0019	0.0019	0.0063
	7	0.0113	0.0063	0.0062
	8	0.1663	0.0108	0.0107
F) South-east Africa	1	0.1239	0.0612	0.0612
	2	0.0629	0.0629	0.0699
	3	0.0076	0.0076	0.0732
	4	0.0093	0.0093	0.0735
	5*	0.5002	0.0685	0.0686
	6	0.2599	0.0897	0.0897
	7	0.0066	0.0066	0.0737
	8	0.0261	0.0261	0.0724
	9	0.0009	0.0009	0.0743
	10	0.0026	0.0026	0.0742
G) Western Europe	1*	0.7471	0.0208	0.0208
	2	0.0949	0.0572	0.0573
	3	0.0934	0.0756	0.0755
	4	0.0188	0.0188	0.0641
	5	0.0276	0.0276	0.0628
	6	0.0041	0.0041	0.0648
	7	0.0005	0.0005	0.0651
	8	0.0114	0.0114	0.0643
	9	0.0018	0.0018	0.0651
	10	0.0004	0.0004	0.0651

For some analyses moderate but non-significant support was also received for particular competing models, besides the significantly best supported model. Specific additional details and interpretations of these selected cases are provided in the following:

B) North American captive: A posterior probability of 0.47 may indicate that the significantly best fitting model, i.e. direct descent from south-east North American wild populations, does not capture all demographic events that shaped the genetic relationship among the populations. This may be influenced by specific breeding-mediated effects that are difficult to model relative to included wild populations. However, inspecting the still moderately supported alternative model revealed that the source of uncertainty in these ABC analyses was the genetic relationship of south-east North American wild populations to other American clusters, rather than their direct ancestry of North American captive strains. This is presumably due to occasional gene flow into south-east North America via the Caribbean (see below). Overall, the significantly best-explaining model and a moderately supported alternative model both favoured south-eastern North America as the direct source of North American captive populations. In contrast, models that considered a different American origin for the North American captive strains were hardly supported.

C) Caribbean: A posterior probability of 0.46 may indicate that the significantly best fitting model, i.e. admixture between populations from central-eastern South America and south-eastern North America, does not capture all demographic events that shaped the genetic relationship among the populations. In agreement with this, two models accounting for gene flow between central-eastern South America and central regions of Central America in the Caribbean, as well as between the latter and south-eastern North America, also received lower non-significant support. This suggests multiple introductions from all three areas into the Caribbean, whereas Central American ancestors appear to have left comparatively limited genetic footprints.

D) Australasia: The support of the significantly best-fitting model was high. Nevertheless, the only competing model that received additional moderate (but non-significant) support is notable in that it considered southern Polynesian populations (New Zealand) as basal and northern Polynesian populations (Hawaii) as the oldest descendent, while the remaining topology was the same as in the selected model. This provides strong support of the chronology of the serial colonisation of the Australasian region overall: starting in Polynesia and successively reaching Asia first, then eastern Australia, and finally western Australia. However, in addition to the major counter-clockwise colonisation of entire Australasia, the possibility that there might have been some translocations also in a clockwise manner specifically across the Pacific Islands may not be excluded.

E) Polynesia: A posterior probability of 0.5 may indicate that the significantly best fitting model, i.e. admixture between populations from north-western South America and western North America, does not capture all demographic events that shaped the genetic relationship among the populations. Indeed, the model that considered admixture between central-east South American and western North American origins also received non-zero, but non-significant support. This may indicate multiple independent historic introductions from South America to Polynesia in addition to (presumably later) introductions from North America.

F) South-east Africa: In addition to the significantly best fitting model of an admixture scenario between west African and Polynesian populations, the model that considered a common ancestor of west African and central-east South American populations contributing to admixture with Polynesian origins as the source of cluster 11 also received non-zero yet non-significant support. This overall strongly supports a west African ancestry, and may indicate that present west African populations have diverged genetically since their original introduction. This may have taken place in the course of a geographic shift from a potentially more southwards region colonised first within Africa, given that South African records of BSF pre-date those from west Africa.

Table S13. Estimates of posterior distributions of population genetic parameters inferred from ABC analyses.

Summary statistics for the posterior distributions of population genetic parameters and the mutation model for the microsatellite markers. Sub-tables A-G correspond to the seven separate analyses and sample selections (N1-N6) as described in Table S11. Additional parameters for the shown best fitting models are denoted as follows: t1-t5: individual splits going back in time (estimated generation times), see also Figure S6; r: admixture rate between two indicated sources, see also Figure S6; μ: mean mutation rate across markers; P: geometric distribution describing the length variation of microsatellite loci; sni: mean single nucleotide indel mutation rate.

Analysis	Parameter	mean	median	mode	q025	q050	q250	q750	q950	q975
A) Americas	N1	2020	1480	1040	310	419	903	2470	5800	7430
	N2	5210	5120	5100	1810	2210	3820	6500	8700	9240
	N3	5150	5030	4770	1780	2190	3780	6360	8720	9300
	N4	6530	6610	6610	2900	3470	5330	7820	9370	9660
	t1	602	547	416	121	171	364	773	1210	1380
	t2	1130	1020	870	339	409	723	1390	2220	2590
	t3	2400	2260	1850	682	853	1550	3120	4470	4730
	μ	0.000241	0.000214	0.000171	0.00012	0.000129	0.000169	0.00028	0.000448	0.000547
	P	0.268	0.28	0.3	0.168	0.193	0.253	0.296	0.3	0.3
	sni	2.71E-06	1.46E-06	2.08E-08	2.56E-08	4.31E-08	3.11E-07	4.56E-06	8.77E-06	9.47E-06
B) North American captive	N1	1560	1240	877	292	372	781	1860	4030	5330
	N2	7470	7690	7920	3870	4500	6560	8670	9590	9760
	N3	4590	4430	4150	1340	1900	3250	5720	8090	8780
	N4	7830	8010	8330	4740	5360	7110	8780	9660	9790
	t1	448	428	268	89	122	265	605	871	916
	t2	1440	1360	1220	530	634	1030	1760	2520	2770
	t3	2230	2070	1710	676	831	1530	2820	4140	4390
	μ	0.00016	0.000147	0.000114	0.000072	0.0000805	0.000113	0.000187	0.000297	0.000346
	P	0.654	0.656	0.684	0.288	0.355	0.516	0.8	0.945	0.97
	sni	1.64E-06	5.41E-07	1.82E-08	1.6E-08	1.95E-08	1.06E-07	2.29E-06	7.35E-06	8.51E-06
C) Caribbean	N1	9360	9470	9680	8080	8490	9160	9690	9890	9960
	N2	283	242	175	72.4	93.7	170	335	524	660
	N3	6800	6910	7140	3100	3810	5730	8020	9330	9580
	N4	8250	8460	8980	5550	6020	7670	9030	9700	9850
	N5	7580	7770	8260	4350	4950	6790	8590	9560	9760
	t1	456	383	236	101	131	250	573	989	1240
	t2	854	744	694	282	335	519	1040	1770	2110
	t3	1230	1000	737	332	392	678	1480	2890	3800
	t4	3350	2750	1880	713	876	1710	4540	7710	8490
	r	0.49	0.492	0.546	0.0684	0.113	0.299	0.664	0.889	0.93
	μ	0.000134	0.000126	0.000114	0.000101	0.000103	0.000113	0.000145	0.000196	0.000226
	P	0.293	0.3	0.3	0.239	0.255	0.294	0.3	0.3	0.3

	sni	2.78E-06	1.7E-06	3.91E-08	3.91E-08	6.5E-08	4.63E-07	4.48E-06	8.55E-06	9.28E-06
D) Australasia	N1	2920	2480	2070	712	881	1670	3710	6590	7690
	N2	2430	2150	1900	744	895	1560	2900	4910	6550
	N3	3720	3340	3060	1040	1290	2320	4730	7710	8750
	N4	6450	6550	6940	2780	3340	5220	7800	9290	9570
	N5	952	545	279	105	135	303	1010	3300	4890
	r	0.776	0.807	0.846	0.364	0.481	0.717	0.874	0.951	0.968
	t1	520	521	463	93	136	338	699	902	947
	t2	171	124	89.8	35.9	43.6	80.6	190	443	624
	t3	2100	1960	1570	667	850	1430	2630	3950	4370
	t4	4140	3810	2790	1270	1500	2680	5260	8050	8910
	μ	0.00016	0.000145	0.000122	0.000103	0.000105	0.000122	0.000179	0.000268	0.000307
	P	0.233	0.24	0.288	0.129	0.145	0.202	0.271	0.295	0.299
	sni	1.09E-06	3.2E-07	1.62E-08	1.33E-08	1.63E-08	7.56E-08	1.31E-06	4.93 E-06	6.48E-06
E) Polynesia	N1	8510	8680	8820	6280	6760	8040	9180	9730	9860
	N2	997	682	500	197	240	445	1080	2800	4360
	N3	3480	3070	2300	862	1080	2100	4420	7600	8610
	N4	4240	3940	3840	1440	1740	2890	5260	7900	8820
	N5	7650	7820	8320	4560	5160	6820	8630	9600	9770
	N6	7320	7470	7870	4090	4700	6430	8350	9540	9750
	t1	849	884	922	485	587	798	940	986	993
	t2	636	528	425	187	224	374	747	1360	1820
	t3	1390	1270	955	553	644	958	1660	2540	2950
	t4	2400	2150	1590	871	1030	1600	2890	4660	5490
	t5	3900	3410	2620	1050	1310	2310	5040	8320	9110
	r	0.675	0.707	0.789	0.198	0.298	0.567	0.816	0.934	0.96
	μ	0.000155	0.000144	0.000128	0.0000762	0.0000837	0.000115	0.000181	0.00026	0.000301
	P	0.601	0.598	0.536	0.249	0.294	0.464	0.739	0.916	0.949
	sni	1.03E-06	2.89E-07	1.22E-08	1.23E-08	1.47E-08	6.06E-08	1.22E-06	4.82E-06	6.3E-06

F) Southeast Africa	N1	8940	9100	9280	7020	7480	8600	9460	9860	9930
	N2	8080	8280	8600	5100	5750	7400	8970	9720	9850
	N3	3560	3220	2850	1080	1300	2270	4450	7330	8360
	N4	2770	2430	2340	772	964	1700	3370	6050	7360
	N5	2710	2310	1750	591	770	1520	3390	6290	7660
	t1	1760	1640	1300	513	674	1190	2220	3220	3630
	t2	2480	2350	1950	1000	1170	1790	3010	4300	4810
	t3	3040	2780	2420	1120	1300	2050	3710	5800	6650
	t4	5950	5900	5790	2290	2700	4430	7440	9370	9680
	r	0.189	0.162	0.151	0.0231	0.0371	0.102	0.241	0.422	0.539
	μ	0.000138	0.000126	0.000113	0.0000672	0.0000739	0.0001	0.000162	0.00024	0.00028
	P	0.464	0.438	0.306	0.173	0.205	0.322	0.583	0.821	0.878
	sni	8.2E-07	2.15E-07	1.0E-08	1.13E-08	1.34E-08	4.65E-08	9.27E-07	3.78E-06	5.33E-06
G) Western Europe	N1	3240	2980	2630	1070	1310	2210	3930	6200	7500
	N2	6680	6730	7000	3540	4020	5650	7760	9220	9560
	N3	2900	2550	2130	853	1050	1810	3510	6280	7600
	N4	1430	1230	1060	485	577	914	1630	2800	3910
	N5	3110	2740	2000	926	1120	1950	3790	6700	8020
	N6	2370	1880	1480	386	526	1170	2910	6330	7740
	t1	690	726	789	198	290	568	850	959	977
	r1	0.426	0.422	0.402	0.111	0.162	0.322	0.523	0.697	0.776
	t2	2020	2050	2150	951	1130	1660	2400	2820	2890
	r2	0.119	0.093	0.0672	0.014	0.0224	0.0574	0.148	0.297	0.395
	t3	4150	4220	4370	2870	3130	3830	4560	4890	4940
	t4	4100	3920	3620	1790	2110	3100	4900	6780	7420
	t5	6230	6160	5430	2660	3130	4760	7730	9450	9690
	μ	0.000198	0.000183	0.000159	0.000111	0.000118	0.000149	0.000229	0.000332	0.000373
	P	0.223	0.229	0.264	0.122	0.134	0.189	0.262	0.292	0.296
	sni	2.9E-06	7.26E-08	1.0E-08	1.02E-08	1.08E-08	2.13E-08	2.79E-07	1.32E-06	1.92E-06

Table S14. Selected group comparisons of inbreeding coefficients F_{IS}.

Weighted linear regression statistics (see Methods for details) of cross-locus inbreeding coefficients F_{IS} for three groups of populations (see Table S3), specified according to captive populations belonging to clusters 1 - 4 (domesticated), global wild populations (wild), and wild-derived captive populations grouping in clusters 5 - 16 (captive wild-derived).

The overall model indicated that inbreeding coefficients significantly differed among the three groups ($F_2 = 16.43$, $p < 0.001$). Estimates of post-hoc Tukey contrasts are reported on the response scale.

Group comparison	Estimate	*P*
Captive wild-derived vs. Domesticated	0.0611	0.0002
Wild vs. Domesticated	-0.0248	0.3542
Wild vs. Captive wild-derived	-0.0858	< 0.0001

Table S15. Group-specific comparisons of variance component ratios for linkage disequilibrium between (D_{ST}) and within (D_{IS}) populations.

Summary statistics of linear mixed effect models inspecting ratios of the variance components for linkage disequilibrium (LD) between (D_{ST}) and within (D_{IS}) populations, accounting for locus pair as a random effect and classified according to captive populations belonging to clusters 1 - 4 (domesticated), wild-derived captive populations assigned to other clusters (captive wild-derived) and global wild populations (wild).

We investigated three models, including different subsets of loci by the groupings specified above: a) all 105 pairs of loci ($F_{2,210} = 3.48$, $p = 0.033$); b) seven pairs of loci repeatedly exhibiting LD across populations assigned to clusters 1 - 4 ($F_{2,14} = 11.17$, $p = 0.001$); and c) all remaining 98 locus pairs ($F_{2,196} = 1.98$, $p = 0.141$). Estimates of post-hoc Tukey contrasts are reported for the log-transformed responses (see Methods for details).

a)

Group comparison	Estimate	*P*
Captive wild-derived vs. Domesticated	0.0737	0.403
Wild vs. Domesticated	0.1511	0.023
Wild vs. Captive wild-derived	0.0775	0.367

b)

Group comparison	Estimate	*P*
Captive wild-derived vs. Domesticated	0.3064	0.039
Wild vs. Domesticated	0.5929	< 0.001
Wild vs. Captive wild-derived	0.2866	0.058

c)

Group comparison	Estimate	*P*
Captive wild-derived vs. Domesticated	0.0571	0.609
Wild vs. Domesticated	0.1196	0.115
Wild vs. Captive wild-derived	0.0625	0.551

Table S16. Microsatellite properties relevant for ABC analyses.

Locus IDs and motif lengths as in Table S2, plus the maximum expected range of motif copies as used for the simulations.

Locus name	Motif length	Max. expected range
MP1_1	3	40
MP1_2	2	40
MP1_3	2	110
MP1_4	2	70
MP1_5	3	40
MP2_1	3	40
MP2_2	2	60
MP2_3	3	40
MP2_4	2	40
MP2_5	3	40
MP3_1	3	60
MP3_2	2	40
MP3_3	2	40
MP3_4	2	40
MP3_5	2	40

Figure S1: Discriminatory power of the novel microsatellite marker set for *Hermetia illucens* genotyping.

Based on the present dataset of 2,862 unique black soldier fly multilocus genotypes, boxplots of the numbers of distinct multilocus genotypes (y-axis) for increasing numbers of any arbitrarily combined loci (x-axis) were calculated. Accordingly, eight arbitrarily chosen loci of the novel microsatellite set resulted in a median discriminatory power of > 97% for distinguishing unique individual multilocus genotypes in the present dataset.

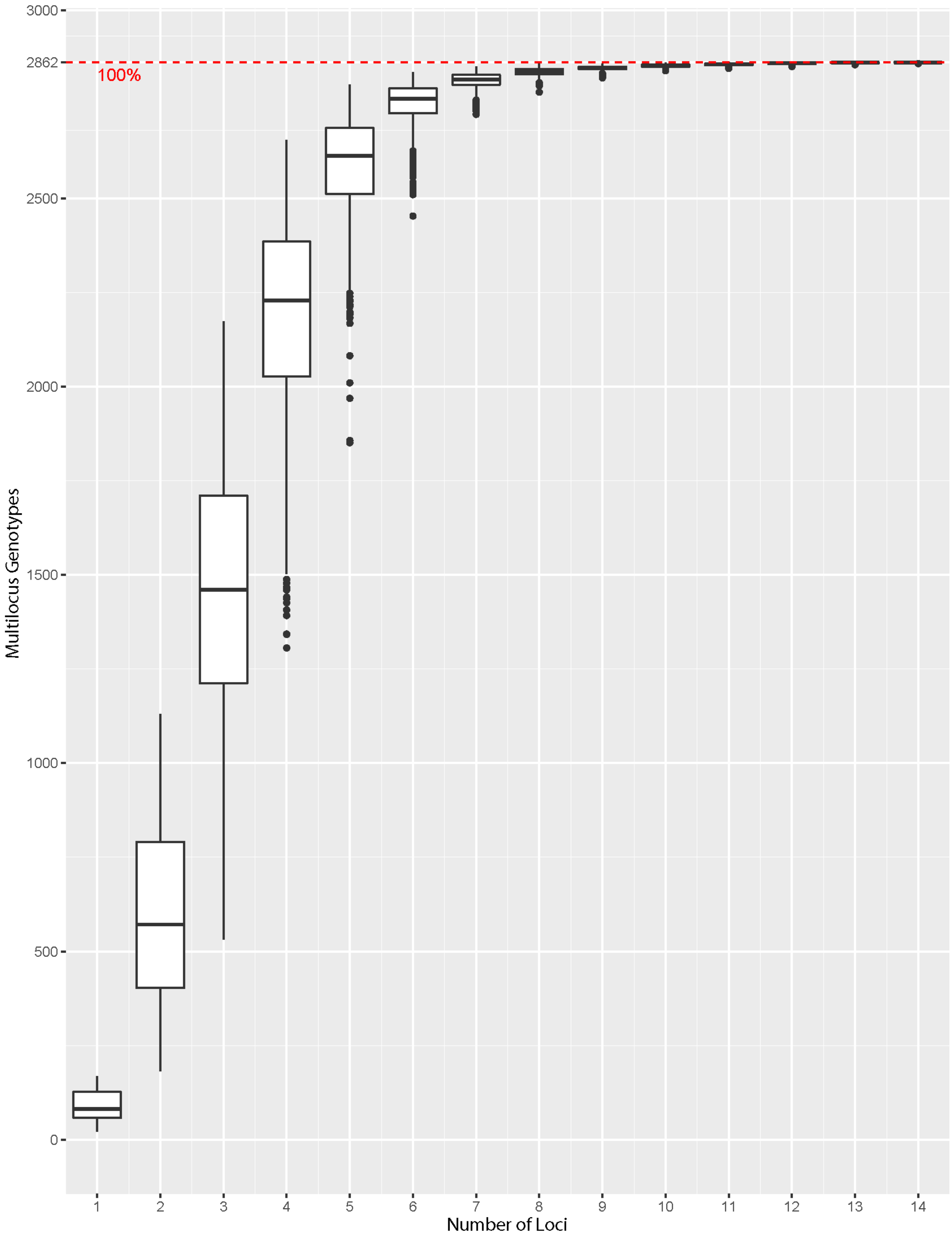

Figure S2. Significant deviations from Hardy-Weinberg equilibrium of individual microsatellite loci tested within populations.

All 150 black soldier fly populations are shown individually (rows; see Table S3) for all 15 loci (columns). *P*-values were adjusted by accounting for 15 simultaneous tests within populations, i.e. with an α-level of $p = 0.0033$, instead of a global adjustment (which might have been too conservative). Non-significant values are highlighted in light blue, significant values are highlighted in dark-blue.

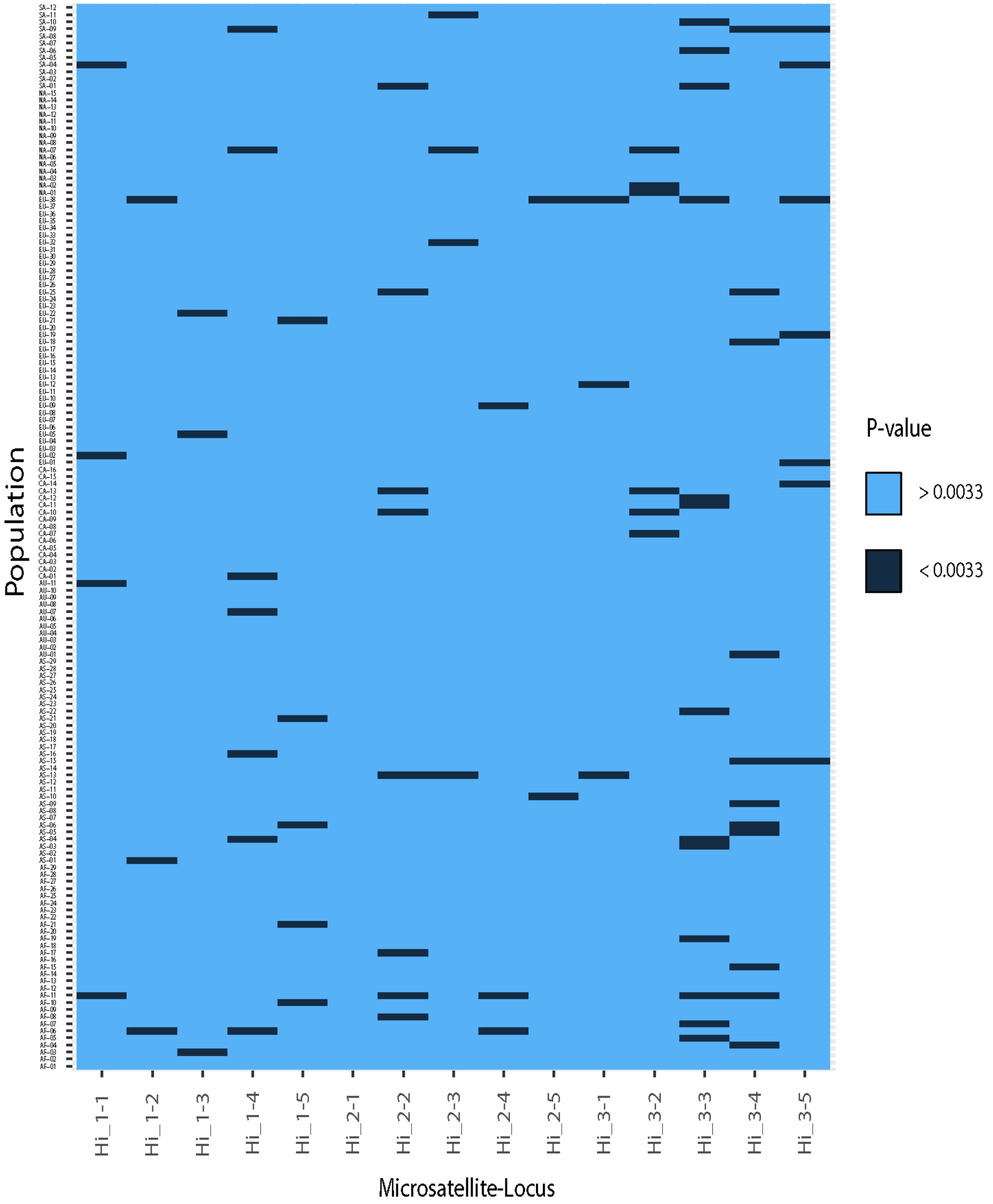

Figure S3. Computational details on genetic cluster analyses and retaining discriminatory functions for visualisation.

A) Inference of the optimal number of clusters of worldwide *Hermetia illucens* populations using the *snapclust* function of the *adegenet* package based on Kullback Information Criterion (KIC) goodness-of-fit statistics for model selection (convergence was verified and only the informative range of *K* between 1-40 is shown). B) Evaluation of the optimal number of discriminatory functions to retain in discriminant analyses of principal components (DAPC) of the 16 genetically distinct clusters (Figure 1) based on the *optim.a.score* function of the *adegenet* package to interpolate optimally performing alpha-scores (here: 65 PCs). Further, we assessed PCs using the *xvalDapc* function of the *adegenet* package, as exemplarily depicted in panels C) and D). First, a larger range in looser intervals was inspected (6 runs with 30 replicates each) and then individual PCs were inspected within a more plausible range of 20-150 (six runs with 50 replicates each). Here, the optimal number of PCs identified ranged from 68-78, and lowest mean square errors (MSE) and values of highest mean assignment success (HMS) across runs both identified 68 as the optimal number of PCs retained in the analysis shown in Figure 1 (MSE: 0.088; HMS: 0.914).

A

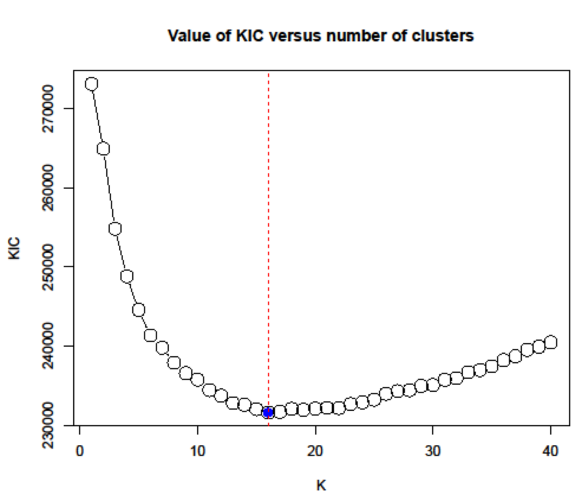

B

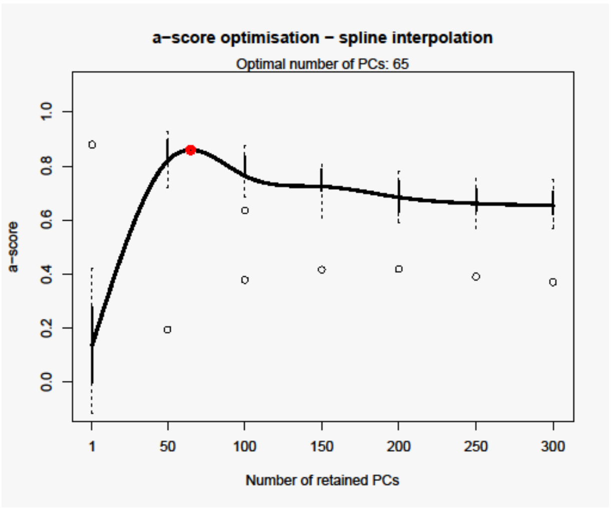

C

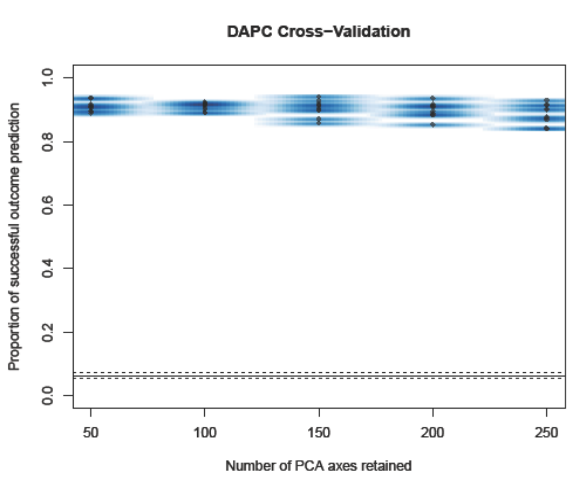

D

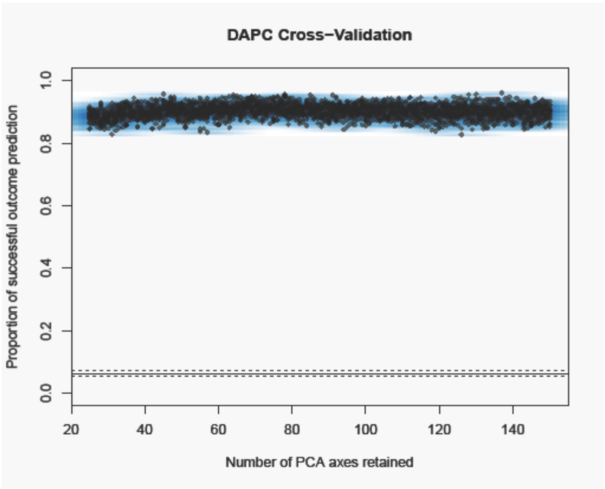

Figure S4. Global population genetic patterns of *Hermetia illucens* according to provenance (wild vs. captive) nested within subcontinent of origin.

Discriminant Analysis of Principal Components based on all 2,862 black soldier fly multilocus genotypes. Individual genotypes were analysed within original population samples (see Table S3). Captive origins are labelled as asterisks, while wild-sourced origins are labelled as dots. Colour allocation is based on original subcontinent origins, following Figures 1, 2 & 3 (see also Table S4). Panel A shows axes 1 and 2, and panel B shows axes 2 and 3. Optimal number of principal components retained were cross-validated, and the relative explained variance for the first three axes is indicated (see smaller integrated boxes).

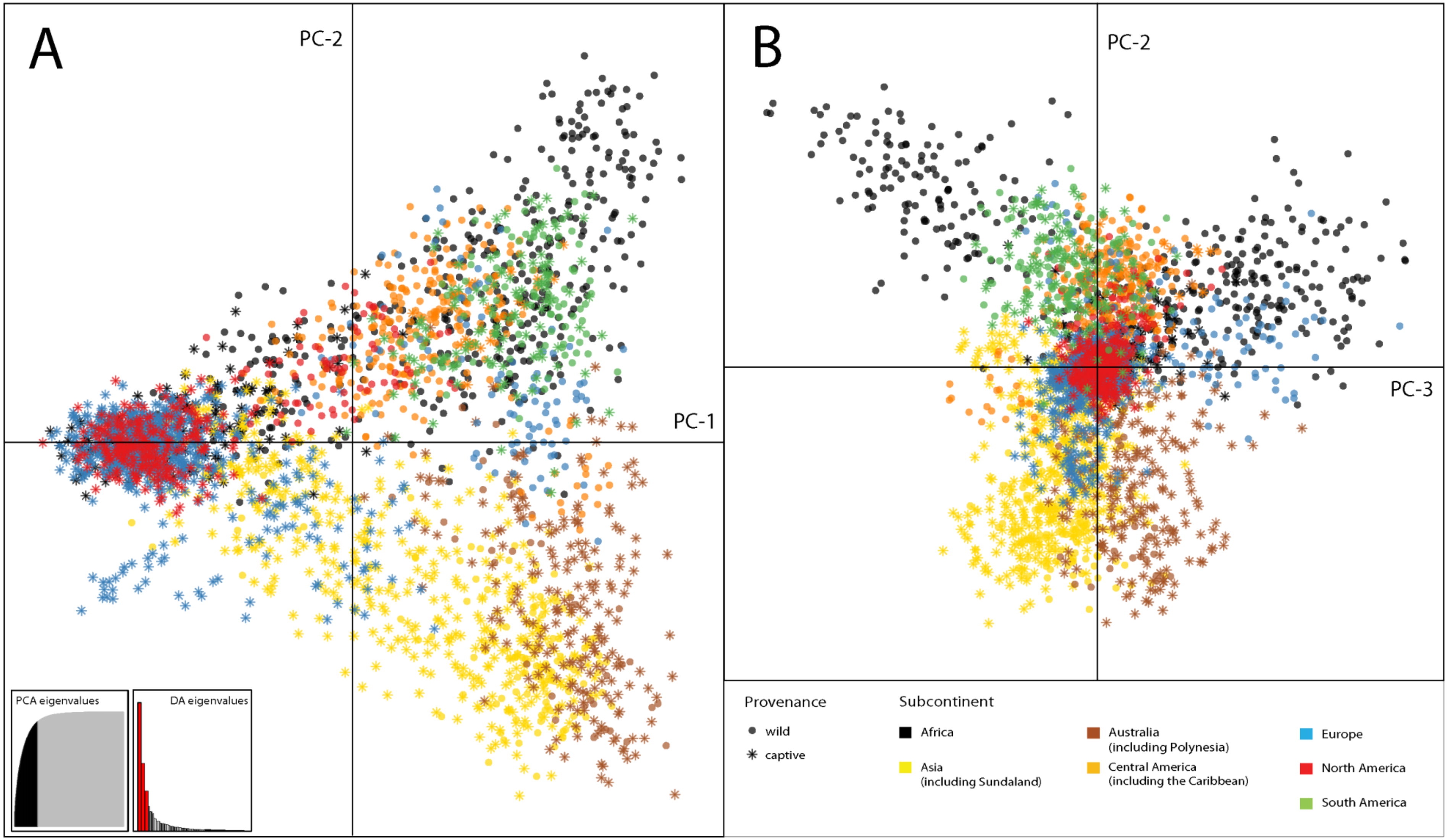

A
PC-2
PC-1
PCA eigenvalues
DA eigenvalues
B
PC-2
PC-3
Provenance
wild
captive
Subcontinent
Africa
Asia (including Sundaland)
Australia (including Polynesia)
Central America (including the Caribbean)
Europe
North America
South America

Figure S5. Neighbour-joining tree based on population pairwise F_{ST} across 150 *Hermetia illucens* populations.

Population provenance is indicated by symbols on each tip label, while colours indicate subcontinental origins as specified in the legend (congruent with Fig. 2 and 3). Labelled clades refer as follows to the groups that were mainly identified by cluster analysis (Fig. 1, Table 2):

- i) Exclusively captive clusters 1 - 3 (domesticated North American origin);
- ii) Exclusively (but one) captive clusters 4 - 5 (hybrids and introgressants between North American domesticated origins and Asian naturalised wild populations of cluster 6);
- iii) Clusters 15 and 16 (indigenous Central and North American populations);
- iv) Clusters 12, 13 and 14 (indigenous South American and southern Central American, as well as naturalised west African populations);
- v) Cluster 11 (south-east African naturalised populations);
- vi) Cluster 6 (naturalised Asian populations, mixed provenances);
- vii) Clusters 7, 8, 9 and 10 (Australian and western European naturalised populations and Caribbean).

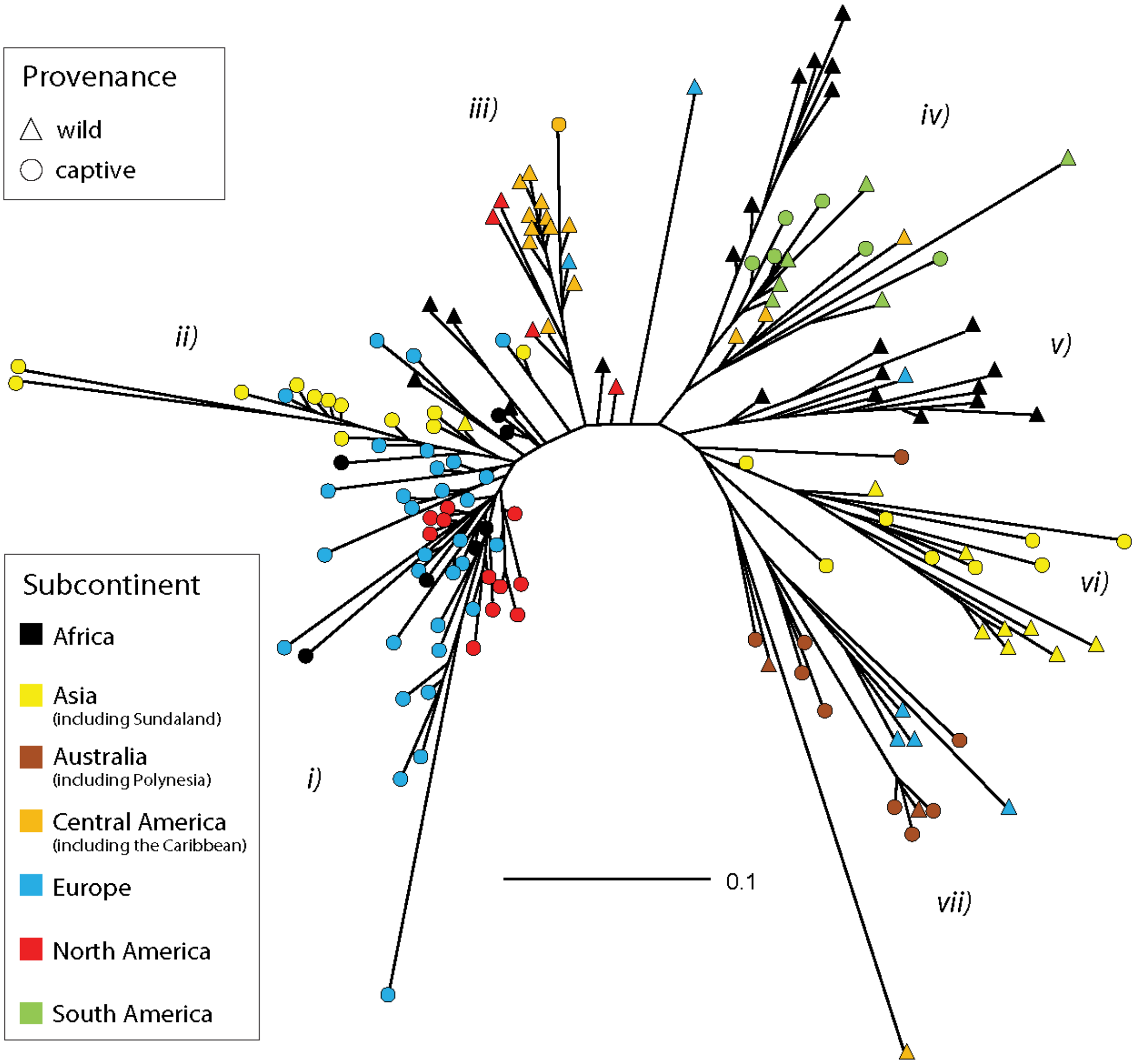

Figure S6. Demographic inference with ABC.

Graphical results of competing demographic models of seven independent analyses are depicted in panels A-G (see Tables S11-S13 for details of addressed models and further results). In each panel, barplots on the left show posterior probabilities with error bars denoting 95% confidence intervals. The significantly best-fitting model is highlighted with an asterisk. Plots on the right show the topologies of the best-fitting model for each of the seven independent ABC analyses. Abbreviations in population-labels refer to subcontinents (AF: Africa; AS: Asia; AU: Australia; CA: Central America; EU: Europe; NA: North America; SA: South America), and specific genetic clusters (1 - 16) are highlighted by coloured numbers based on Figure 1.

A) Americas

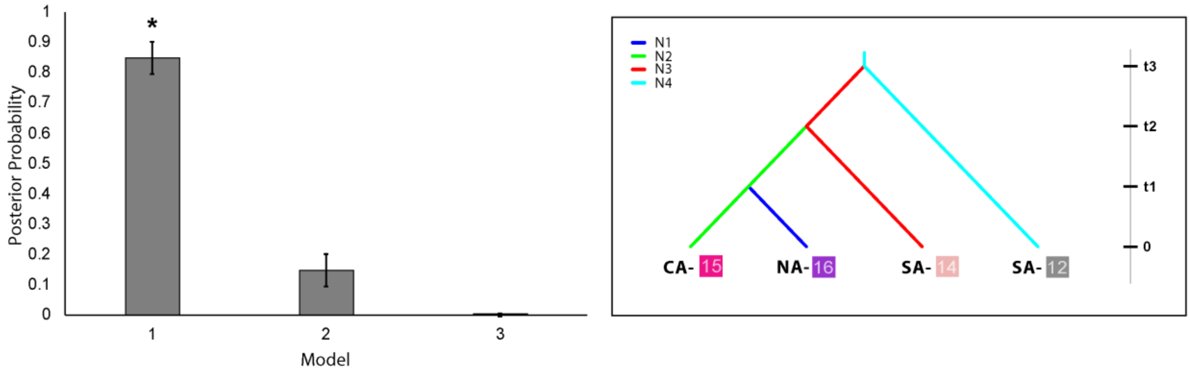

B) North American captive

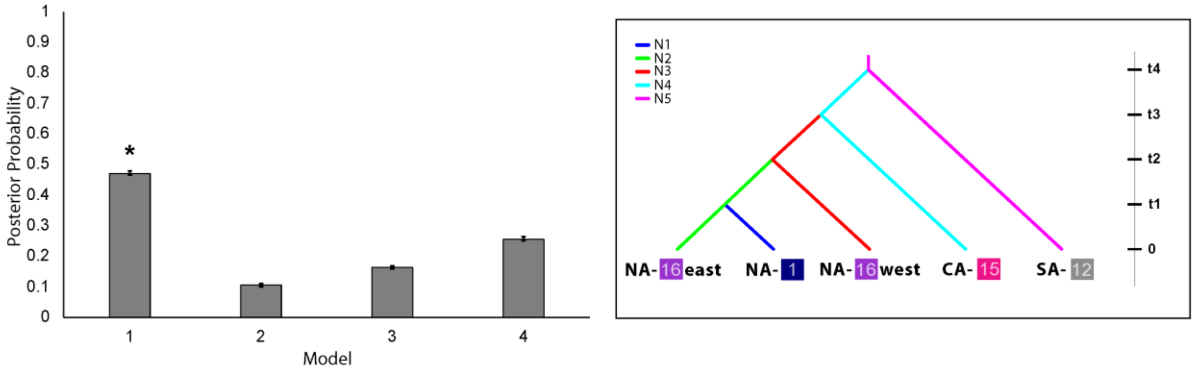

C) Caribbean

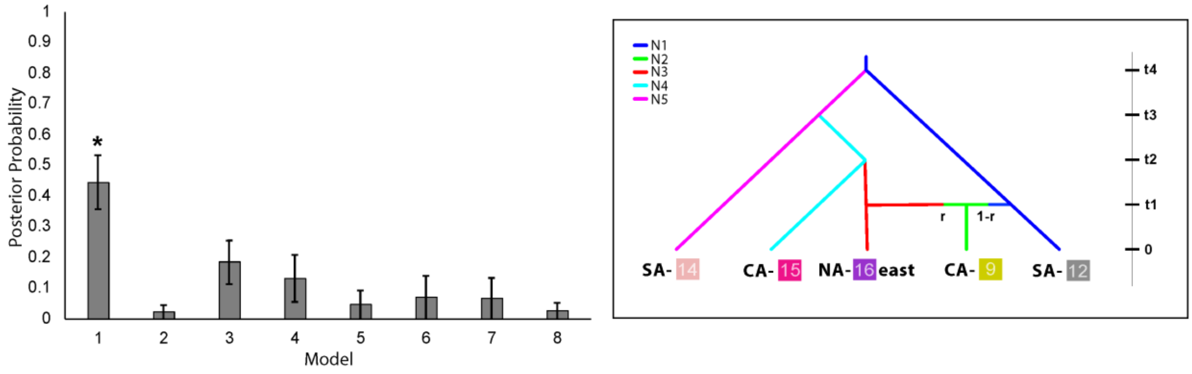

D) Australasia

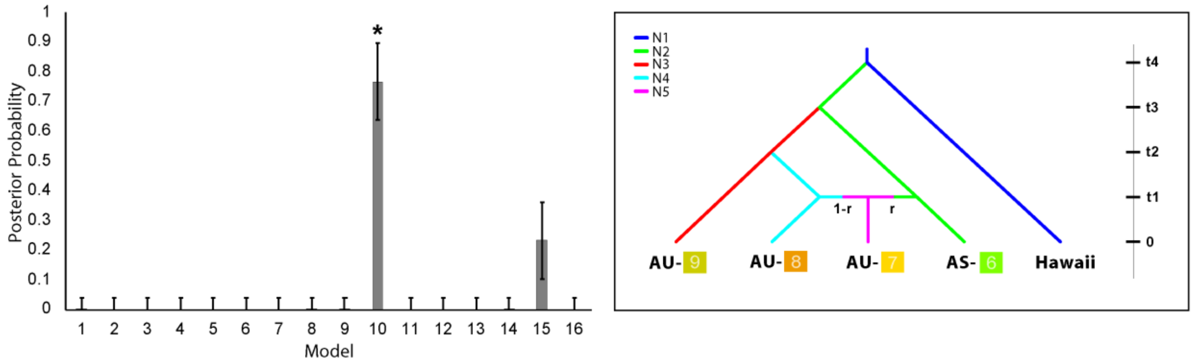

E) Polynesia

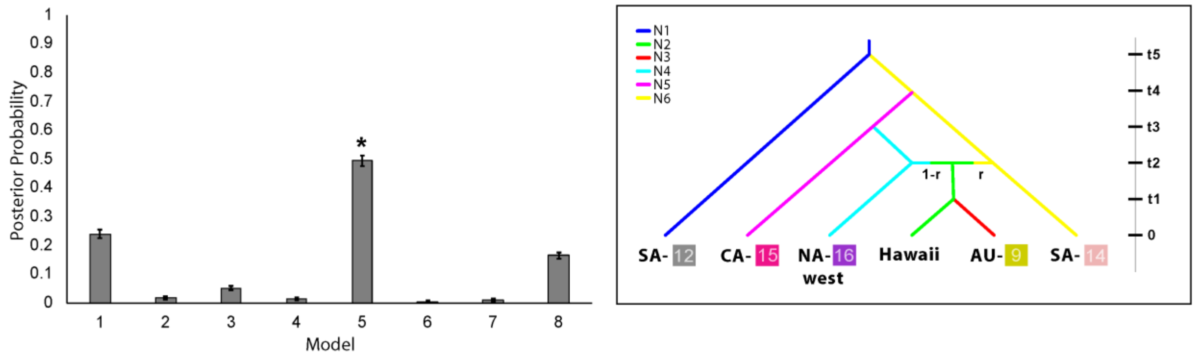

F) south-east Africa

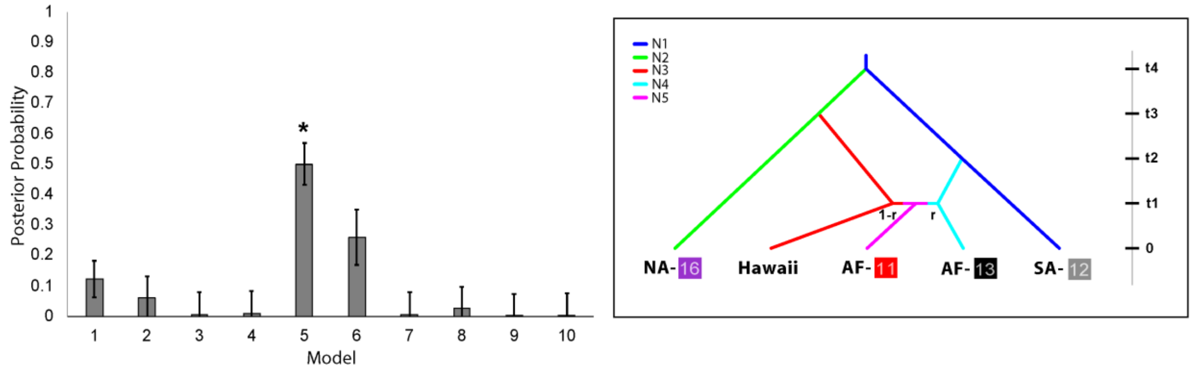

G) western Europe

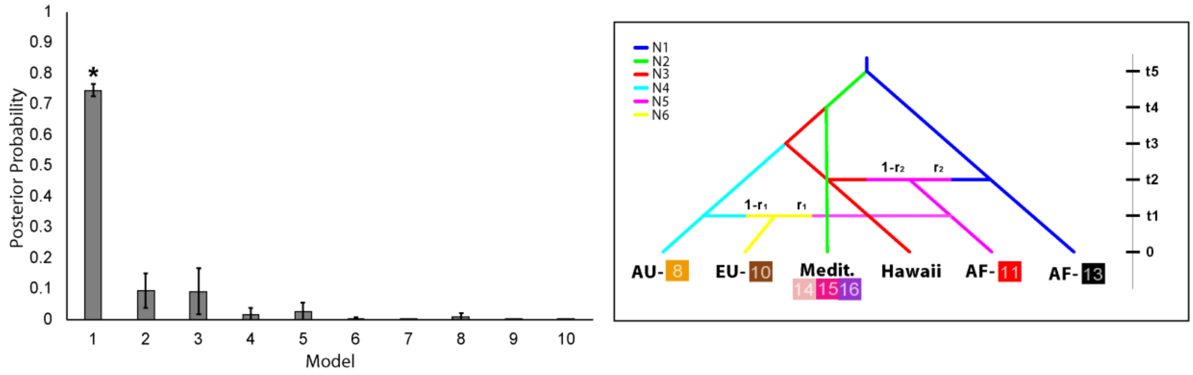

Figure S7. Detection of hybrids and backcrosses: a west African case of introgression.

Inferences of hybrids and backcrosses based on ancestry coefficients for two west African wild populations that were distinct from the regionally naturalised cluster 13 and exhibited admixture between cluster 2 and 16 according to Figures 1 & 3. Captive populations of cluster 2 were introduced to a farming facility in their vicinity two years prior to sampling. Thus, the two wild populations suspected to be admixed were compared to their two traceable European sources and to the two geographically closest wild populations assigned to the west African naturalised cluster 13 (as most probable parental groups). Independent of previous cluster allocations for each sampled population (based on the majority of individuals), all individuals were re-assigned to either parental group, F_1 hybrids (balanced admixture) or backcrosses with either parental group (0.75 : 0.25 admixture).

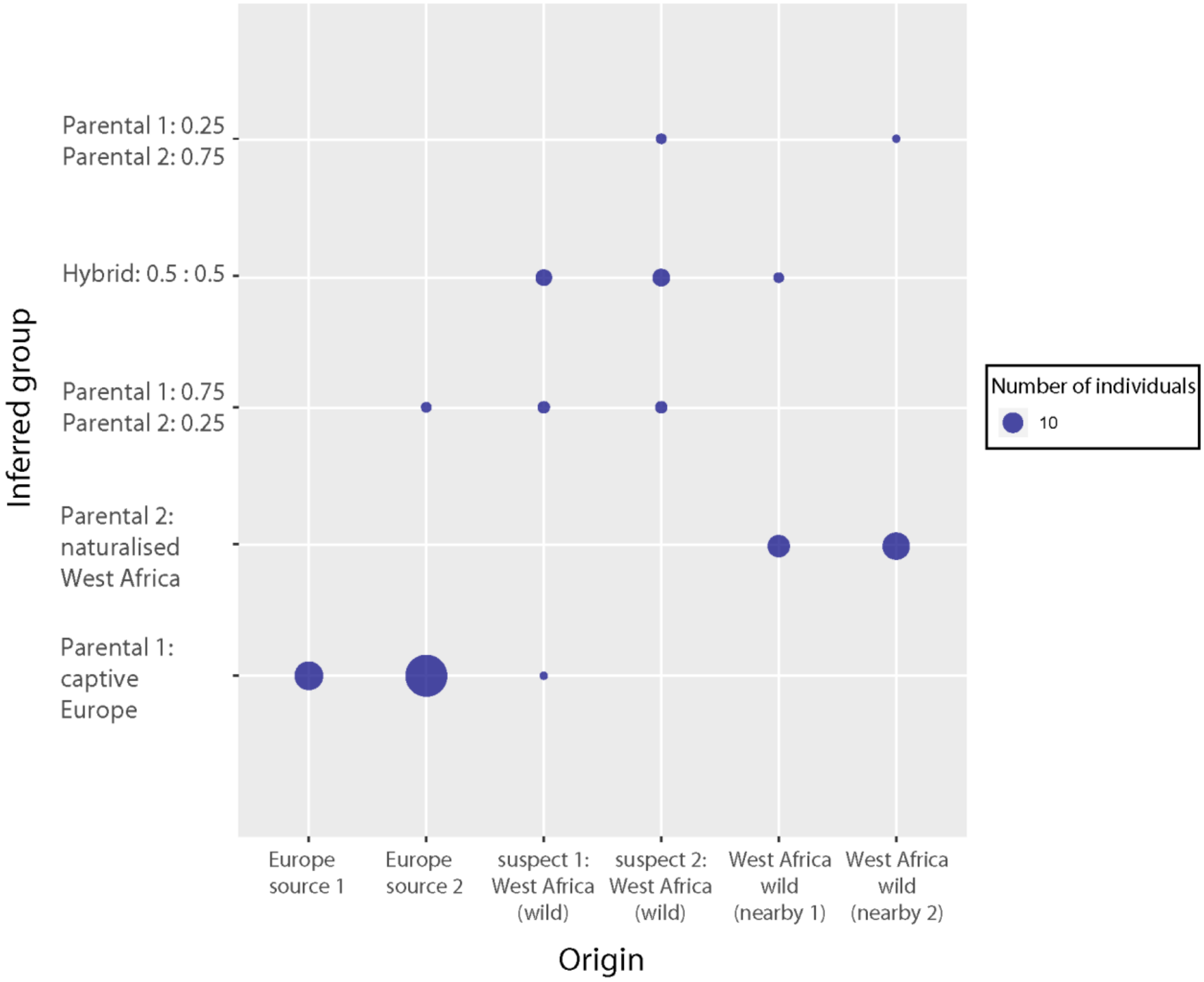

Figure S8. Detection of hybrids and backcrosses: case-specific analyses of central-east African populations and predominantly farmed populations from Asia.

Inferences on hybrids and backcrosses based on ancestry coefficients for potentially introgressed groups of interest relative to recently introduced domesticated clusters 1, 2 and 3 (Figure 1). A: all African populations allocated to cluster 16 (by the majority of individuals), including south-east African naturalised cluster 11 as the most probable second parental group; B: entire clusters 4 and 5 (all but one population reared in captivity), including the Asian naturalised cluster 6 as the most probable second parental group.

Independently of previous cluster allocations for each sampled population, all individuals were re-assigned to either parental group, F_1 hybrids (balanced admixture) or backcrosses towards either parental group (0.75 : 0.25 admixture). Since cluster membership of original populations was based on the majority of respective individuals, some individuals from populations of supposedly parental ancestry were re-allocated to admixed groups in this analysis. This was particularly pronounced in European captive populations, where recent introgression from both African and Asian origins appears to be frequent; see Figure 1C and main text in the paper.

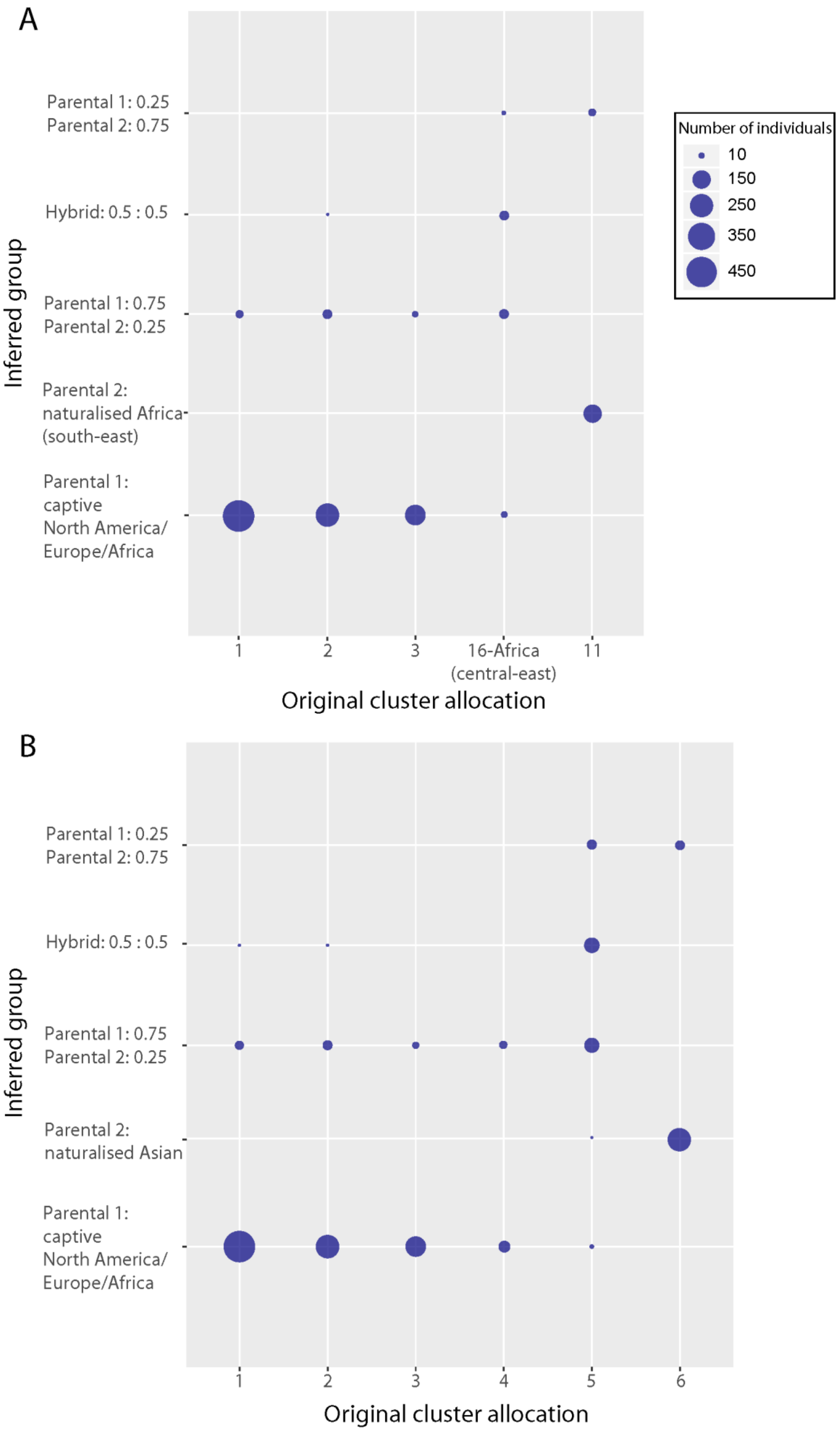
A
Parental 1: 0.25
Parental 2: 0.75
Hybrid: 0.5 : 0.5
Parental 1: 0.75
Parental 2: 0.25
Parental 2: naturalised Africa (south-east)
Parental 1: captive North America/ Europe/Africa
Inferred group
1
2
3
16-Africa (central-east)
11
Original cluster allocation
Number of individuals
10
150
250
350
450
B
Parental 1: 0.25
Parental 2: 0.75
Hybrid: 0.5 : 0.5
Parental 1: 0.75
Parental 2: 0.25
Parental 2: naturalised Asian
Parental 1: captive North America/ Europe/Africa
Inferred group
1
2
3
4
5
6
Original cluster allocation
